# Physical activity and sedentary time of youth in structured settings: a systematic review and meta-analysis

**DOI:** 10.1186/s12966-020-01054-y

**Published:** 2020-12-04

**Authors:** Rafael M. Tassitano, R. Glenn Weaver, Maria Cecília M. Tenório, Keith Brazendale, Michael W. Beets

**Affiliations:** 1 Department of Physical Education, Federal Rural University of Pernambuco, SN Dom Manoel de Medeiros St, Recife, PE 52171-900 Brazil; 2 University of South Carolina, Columbia, SC USA; 3 University of Central Florida, Orlando, FL USA

**Keywords:** Physical Activity, Sedentary time, Youth, Structured settings

## Abstract

**Background:**

Structured settings, such as school, childcare, afterschool programs, summer camps, and physical activity/sport programs are crucial to promoting physical activity (PA) opportunities and reducing sedentary (ST) for children and adolescents. However, little is known about how much PA and ST children and adolescents accumulate in structured settings. The aim of this study is to conduct a systematic review and meta-analysis of the absolute amount of time youth spend physically active and sedentary in different structured settings (Prospero number: 42018111804).

**Methods:**

Observational and experimental/quasi-experimental studies (baseline data only) with full-text available, written in English and published in a peer-reviewed journal, reporting the total amount of objectively measured PA (light, moderate, vigorous, and/or total physical activity) and/or time spent ST during structured settings among youth (3 to 18 years) were eligible. Adjusted meta-analysis was conducted to estimate the pooled mean of time spent in PA and ST, by settings and sex.

**Results:**

A total of 187 studies (childcare *n*=60; school *n*=91; afterschool programs *n*=14; summer camp *n*=4; and Physical activity/ sport programs *n*=18) from 30 countries (47.9% United States), representing 74,870 youth (mean age 8.6 years old) were included. Overall, there was a high variation between studies in outcomes and settings. The meta-analyses revealed, on average, youth spend 221.8 minutes (36.7 min/hour) in ST and 32.1 minutes (5.1 min/hour) in MVPA during childcare hours, and 223.9 minutes (36.7min/hour) in ST and 27.8 min (4.4 min/hour) in MVPA at school. Relatively, youth are engaged in more MVPA in afterschool programs (11.7 min/hour), PA/ sport programs (20.9 min/hour), and summer camps (6.4 min/hour), when compared to childcare and school.

**Conclusion:**

Total PA accumulated during childcare and MVPA accumulated during schools hours were close to recommendations, despite high proportion of ST. Afterschool programs, summer camp and PA/ sport programs are important settings that can contribute to daily PA and reduced ST. Ensuring all youth have access to these structured settings may be an important step forward for public health.

**Supplementary Information:**

The online version contains supplementary material available at 10.1186/s12966-020-01054-y.

## Introduction

Health benefits related to regular engagement in physical activity (PA) and reducing sedentary time (ST) during childhood and adolescence are well documented [1–4]. Despite that, most youth around the world do not meet recommendations for PA [5–7], and significant declines in PA have been observed as youth age [8–10], and as a consequence ST increases [8, 9, 11]. Thus, promoting regular opportunities for daily PA engagement and reduced ST during childhood and adolescence continues to be a significant public health challenge.

Structured settings (i.e. school, childcare, afterschool programs, summer camps, and PA/sport programs) are defined as pre-planned, segmented, and adult supervised environments [12], and are the focus of most studies concerned with increasing PA in youth [13–18]. The focus on structured settings makes sense for several reasons. First, almost all children and adolescents spend a large proportion of their waking time in these setting on most days and months of the year [19, 20]. Second, structured settings typically have the infrastructure and resources in place to promote PA and reduce ST. Finally, structured settings have broad reach with most children attending one or more structured setting most days of the week (e.g., day/childcare, schools).

However, little is known about how much PA and ST children accumulate in structured settings. While systematic reviews of PA and ST in childcare [21–23], school [15], and afterschool [16] have been published, none have identified the amount of time youth spend in PA and/or ST while attending these settings. Other reviews have focused on the time children are activity during specific segments of the school day, such as physical education class [24, 25], school playtime [26] and recess [27].

Understanding the amount of PA and ST children accumulate while attending structured settings is a crucial first step for designing more effective interventions and can help to identify which structured settings should be targeted for intervention in order to promote PA and reduce ST. Past systematic reviews are limited because they focus on total daily PA or ST [15, 21–23] without identifying the contribution of the structured setting to these estimates of PA and/or ST, were limited to specific behavior or intensity of PA [15], were largely based on subjective measures of PA and/or ST [16], and/or did not meta-analytically synthesize the findings [21–23]. In light of these limitations, the aim of this study is to conduct a systematic review and meta-analysis to summarize the amount of PA (i.e., minutes of light, moderate, vigorous, MVPA and total PA) and ST that youth accumulate while attending structured settings.

## Methods

The present systematic review and meta-analysis is registered in International Prospective Register of Ongoing Systematic Reviews (PROSPERO) under the number CRD 42018111804 and reported in accordance with the Preferred Reporting Items for Systematic Reviews and Meta-Analysis (PRISMA) statement [28] (Additional file 1) and Meta-analysis of Observational Studies in Epidemiology guidelines (MOOSE) [29]. All authors agreed to the protocol before starting the search.

Observational and experimental/quasi-experimental studies (only baseline data) with available full-text, written in English and published in a peer-reviewed journal were included. Studies reporting the total amount in minutes of any PA intensity (i.e. light, moderate, vigorous or combined) and /or ST measured by objective wearable device (i.e., accelerometer, and heart rate monitor) during a structured setting among youth aged 3 to 18 years were eligible. Studies with children or adolescents in clinical settings, with disabilities, and /or institutionalized were excluded. A structured setting was defined as a context that provides a pre-planned, segmented and adult supervised component(s) (i.e. childcare, school, afterschool program, summer camp, PA/ sport program) [12]. A context-specific definition of each structured setting is presented below:

*Childcare:* Structured, adult supervised setting that cares for children (i.e., typically 3-5 years) as a service for working parents, and operates at a school, home, or center during weekdays, and provides a variety of different pre-planned, segmented activities for children.

*School:* Formal educational institution regulated by educational policies and agencies with compulsory activities during segmented times (typically on weekdays) throughout an academic year (typically 9 months a year).

*Afterschool program:* Community-based program that takes place immediately after regular school day and available daily throughout the academic year (Monday through Friday); and provides a combination of scheduled activities, which commonly include a snack, homework assistance/tutoring, enrichment activities, and opportunities for children to be physically active [30].

*Physical activity and/or sport programs:* Pre-planned, segmented, and adult supervised program with a singular focus on a specific PA or sport (i.e. soccer, dance, baseball, netball, flag football) delivered in a single day session or multiple day sessions during a week throughout the year. Programs typically consist of practices and formal competitions.

*Summer day camp:* No residential or sleepover programs that serve school-aged children as a service for working parents operating during summer vacation from school and provide a variety of pre-planned, segmented activities such as PA/sports, art, and/or academics [31].

The first author (RMT) conducted the search from October 2018 to February 2019 using four electronic databases: (1) MEDLINE via PubMed, (2) SCOPUS, (3) Web of Science, and (4) Cochrane. Four groups of search terms (Outcome, Structured Setting, Measure, Population) were combined using Boolean operators (Additional file 2).

The search results were imported into EndNote X7 (Thompson Reuters, San Francisco, CA, USA), and conducted the following steps: 1) All duplicate studies were removed. 2) Titles and abstracts were screened by two independent reviewers (RMT, MCMT) to identify potential articles based on the review question. 3) Studies that did not meet the eligibility criteria were removed. 4) Full text papers of potentially eligible studies were assessed. 5) The references of all included studies were reviewed to identify additional studies. 6) Consensus on all full-text papers excluded was reached via weekly group discussion with all authors (RMT, RGW, MCMT, KB, and MWB). Information about the article (title, year of publication, and authors), data collection (country, and global region), structured settings (childcare, school, afterschool program, summer camp, sport program), sample information (sample, sex, age, race, and socioeconomic status), protocol measure (manufactures, and data reduction procedures), PA (light, moderate, vigorous and total PA), and ST were extracted and entered into a custom Excel spreadsheet created for this study. For studies that provided other metric (i.e. min/ hour or percent) and wear time during attendance, the total amount in minutes was calculated. Studies using only a proxy reporting procedure only (i.e. the length of setting attendance) were excluded. If necessary, the authors of included studies were contacted by e-mail to provide necessary additional information.

For analysis, when studies reported mean age the nearest year was extracted. Where age was not reported, grade level was used to infer the age [32]. Where necessary standard deviations were calculated from confidence intervals (95%CI), standard errors (SE), etc. based on Cochrane handbook guidelines [33]. When standard deviation, 95%CI, or SE were not reported, the standard deviation (SD) was estimated by predicting the sample-weighted coefficient of variance for each outcome of all studies in each setting [32] and computing the standard deviation using this estimate.

The risk of bias was assessed using a tool (Additional file 3) developed for the systematic review, and was created based on the moderators used in the meta-regression, and previous systematic reviewers [24, 25]. The tool consisted of 11 item covering study (i.e. design), sampling, structured setting, objective-measure protocol, and report outcomes, and was created based on the covariates used in the adjusted model of the meta-analysis. Each criteria was evaluated by two independent reviewers and scored as “presented and adequately described” (yes = 2), “not clear described or presented” (yes, partially = 1), or “not reported” (no = 0), and the final scored ranged between 0 to 22. A third reviewer was consulted if there is no consensus between the first two reviewers.

### Statistical analyses

Studies that presented mean and standard deviation of PA and/or ST reported minutes were included in the Meta-Analysis. Studies that reported the outcome through other metrics (e.g. percent (%), total minute by weekday in structured setting) were included in the analyses if they provided enough information to calculate the daily minutes accumulated in the setting of interest. Data were distilled separately for each structured setting.

The meta-analyses were performed in R (http://cran.r-project.org) using the robumeta, metafor and dplyr packages. Adjusted pooled means were calculated to estimate the absolute (minutes) and relative (min/hour) amount of ST and PA that children/ adolescents accumulated in each structured setting using random-effects models. Minute by hour of PA and ST was estimated by dividing the total wear time by the mean PA and ST estimate. Sex, age, sample size, study design, global region, accelerometer brand, weartime, cut-point, and risk of bias were used as covariates in the models for all outcomes and SS when appropriate and possible. The I^2^ index was used to identify the heterogeneity considering values of 25%, 50% and 75% to represent low, moderate or high, respectively [33]. Additionally, due to the variability between studies meta-regression analyses were conducted to verify each potential moderators by outcome and structured settings.

## Results

**Fig 1:**
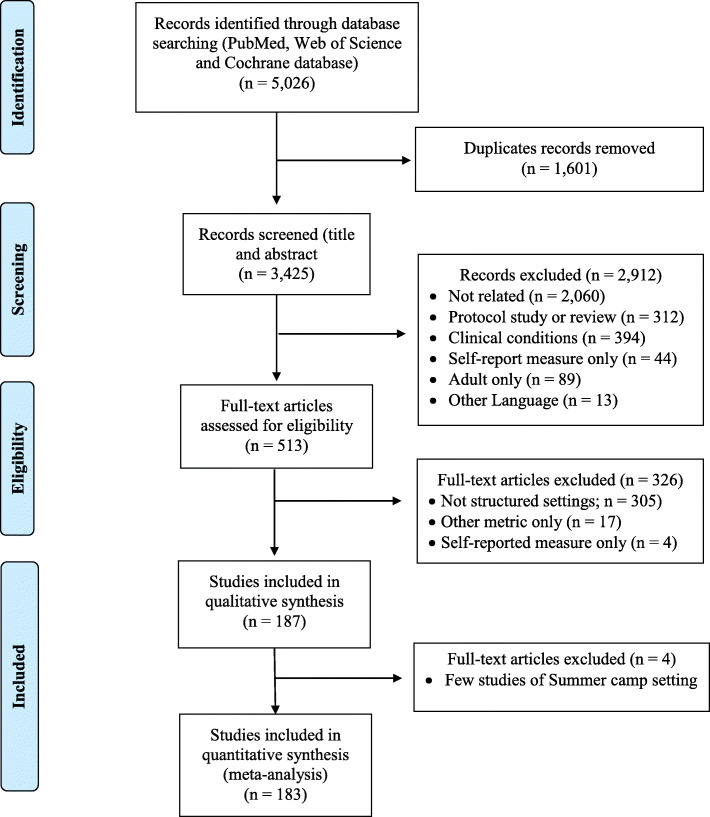
PRISMA flow diagram of study selection process

A total of 5,026 records were found, and after excluding duplicate articles and those that did not meet the inclusion criteria, 187 studies were included in the qualitative synthesis. For the meta-analysis the summer camp setting were excluded due the few included studies, and all other studies from childcare, school, afterschool and physical activity/sports programs were included for the analyses (see Figure 1). Considering the structured settings, 48.7% of the studies were conducted in schools (*n*=91), 32.1% in childcare (*n*=60), 9.6% in sport programs (*n*=18), 7.5% in afterschool programs (*n*=14), and 2.1% in summer camps (*n*=4). A summary of the descriptive characteristics (i.e. author, year of publication, global region, study design, sample size, sex, age, device brand, cut-point, and risk of bias) by structured setting are presented in the Table 1. The descriptive characteristics of all included studies are presented in Table 1s. General information about the measure of PA and ST (i.e. measure protocol, sample size, and average of valid wear-time during setting) and outcomes (e.g. mean and SD of ST, LPA, MPA, VPA, MVPA and TPA) are presented in Table 2s. The heterogeneity (I^2^) of structured settings and outcomes ranged between 54.4% to > 90%. Adjusted pooled mean (absolute and relative) estimates are presented in Table 2, considering the PA level and ST accumulated during attendance by structured settings and sex. The meta-regression analyses are presented in Tables 3s, 4s, 5s, 6s.

**Fig 2:**
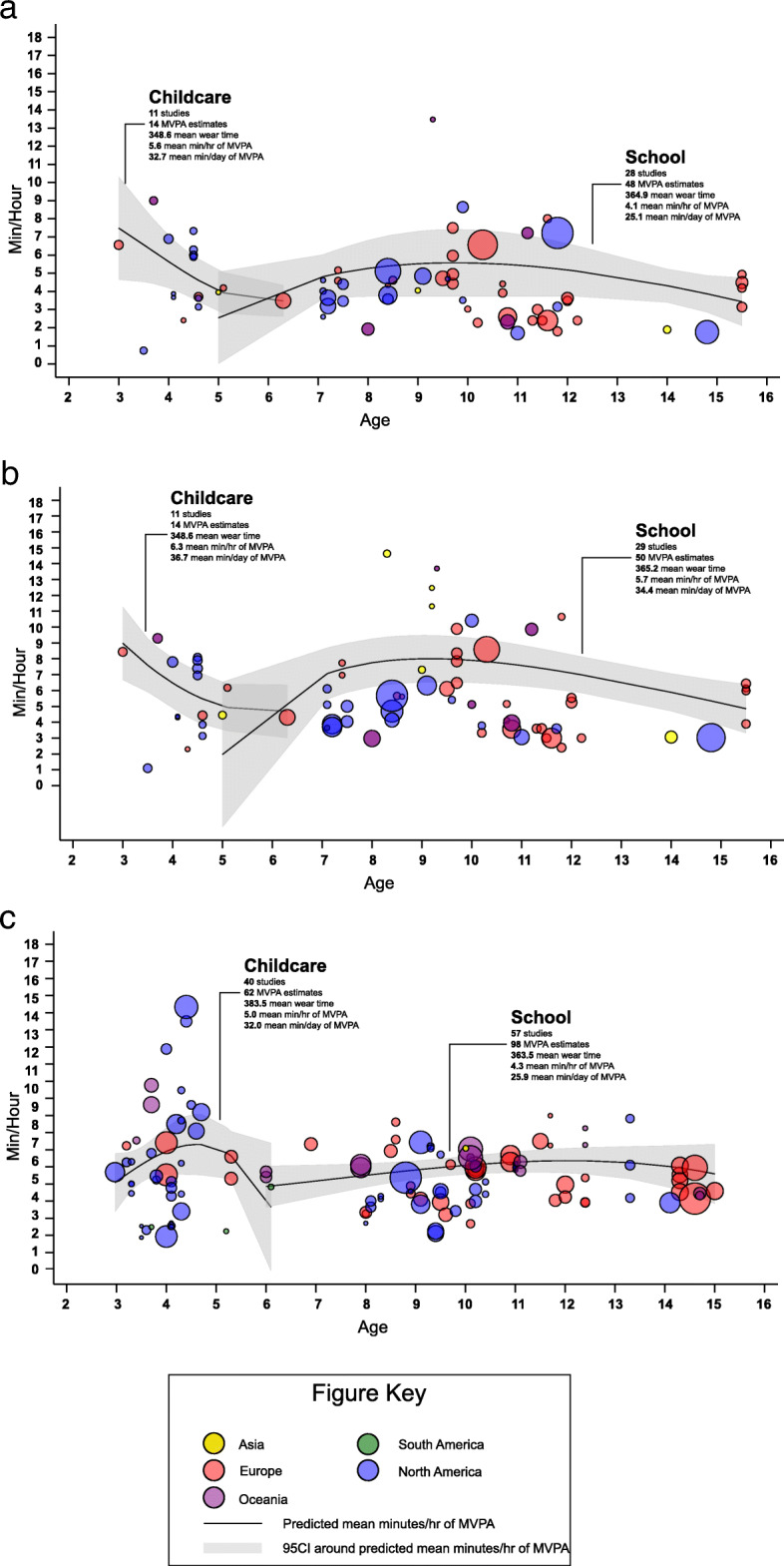
**a**-**c**. Scatter plot and estimated mean min/hour of moderate to vigorous physical activity by age during childcare and school. **a** = Girls – studies that provide girl specific estimates of MVPA. **b** = Boys – studies that provide boy specific estimates of MVPA. **c** = Total – studies that combine boys and girls together estimates of MVPA

**Table 1:** Descriptive table of global region, study design, sample size, sex, age, device brand, cutpoints, and risk of bias by structured settings

	Childcare (*n* = 60)		School (*n* = 91)		Afterschool program (*n* = 14)		Summer Camp (*n* = 4)		Physical activity/ Sports program (*n* = 18)	
	n	(%)	n	(%)	n	(%)	n	(%)	n	(%)
**Global Region**										
North America	42^a^	(70.0)	35^a^	(38.4)	13	(92.9)	4	(100)	12	(66.7)
Europe	10^a^	(16.6)	40	(43.9)	--	--	--	--	4	(22.2)
Oceania	7^a^	(11.7)	10	(11.1)	1	(7.1)	-	-	2	(11.1)
South America	1	(1.7)	1	(1.1)	-	-	-	-	-	-
Asia	--	--	5	(5.5)	--	--	--	--	--	--
Africa	--	--	--	--	--	--	--	--	--	--
**Study design**										
Cross-sectional	46	(76.7)	60	(65.9)	10	(71.5)	4	(100)	17	(94.4)
RCT	11	(18.3)	11	(12.1)	2	(14.3)	--	--	1	(5.6)
Longitudinal	3	(5.0)	7	(7.7)	1	(7.1)	--	--	--	--
Intervention	--	--	8	(8.8)	--	--	--	--	--	--
Quasi-experimental	--	--	4	(4.4)	1	(7.1)	--	--	--	--
Natural-experiment	--	--	1	(1.1)	--	--	--	--	--	--
**Sample size (*n*)**	14,763		42,463		12,021		3,330		2,293	
< 30	--	--	--	--	--	--	--	--	1	(5.6)
30 – 200	31	(51.6)	33	(36.3)	5	(35.7)	3	(75.0)	13	(72.2)
201 – 350	15	(25.0)	19	(20.9)	2	(14.3)	0	(0)	3	(16.6)
351 – 600	11	(18.4)	19	(20.9)	2	(14.3)	0	(0)	0	(0)
> 600	3	(5.0)	20	(21.9)	5	(35.7)	1	(25.0)	1	(5.6)
**Sex**						-				
Boys	7,765	(52.6)	18,514	(43.6)	5,998	(49.9)	1,825	(54.8)	1,321	(57.6)
Girls	6,998	(47.4)	23,949	(56.4)	6,023	(50.1)	1,505	(45.2)	972	(42.4)
**Age (mean)**	4.2	(0.7)	10.1	(2.1)	8.3	(1.3)	8.8	(1.4)	11.6	(2.0)
**Device brand**										
Actical	14^b^	(23.0)	2	(2.1)	-	-	-	-	1	(5.6)
Actigraph	42	(68.9)	80^b^	(83.5)	14	(100)	4	(100)	16	(88.8)
Actiheart	1	(1.6)	--	--	--	--	--	--	--	--
Actipal	2^b^	(3.3)	1^b^	(1.0)	--	--	--	--	--	--
Actitrainer	1	(1.6)	5^b^	(5.3)	--	--	--	--	--	--
RT3	1	(1.6)	1	(1.0)	--	--	--	--	--	--
SWM	--	--	1	(1.0)	--	--	--	--	--	--
NL-100	--	--	1	(1.0)	--	--	--	--	--	--
HJA-3501T	--	--	1	(1.0)	--	--	--	--	--	--
Polar	--	--	1	(1.0)	--	--	--	--	1	(5.6)
Geneactiv	--	--	2	(2.1)	--	--	--	--	--	--
RT3	1	(1.6)	1	(1.0)	--	--	--	--	--	--
**Cut-points** ^c^										
Freedson	--	--	8	(8.5)	3	(15.8)	2	(25.0)	8	(40.0)
Trost	1	(1.6)	5	(5.3)	1	(5.3)	--	--	--	--
Evenson	5	(8.1)	43	(45.7)	7	(36.8)	1	(12.5)	9	(45.0
Matthews	--	--	4	(4.3)	4	(21.1)	--	--	--	--
Pate	17	(27.4)	--	--	1	(5.3)	--	--	--	--
Pfeiffer	11	(17.7)	--	--	--	--	--	--	--	--
Puyau	1	(1.6)	5	(5.3)	2	(10.5)	1	(12.5)	2	(10.0)
Van Cauwenberghe	7	(11.3)	--	--	--	--	--	--	--	--
Treuth	--	--	4	(4.3)	--	--	1	(12.5)	1	(5.0)
Sirard	14	(22.6)	--	--	--	--	--	--	--	--
Troiano	--	--	1	(1.1)	--	--	1	(12.5)	--	--
Other	6	(9.7)	24	(25.5)	1	(5.3)	2	(25.0)	--	--
**Risk of bias (mean)**	19.1	(1.7)	18.1	(2.3)	20.5	(1.7)	18.6	(1.6)	19.7	(1.4)
**Risk of bias**										
≤ 18	20	(33.3)	45	(49.4)	2	(14.3)	8	(13.3)	1	(25.0)
19 - 20	27	(45.0)	36	(39.6)	3	(21.4)	7	(11.7)	2	(50.0)
≥ 21	13	(21.7)	10	(11.0)	9	(64.3)	3	(5.0)	1	(25.0)

^a^ Study with sample from two different countries.

^b^ Two different devices were used in the same study.

^c^ Based on studies that reported at least one cut-point.

**Fig 3:**
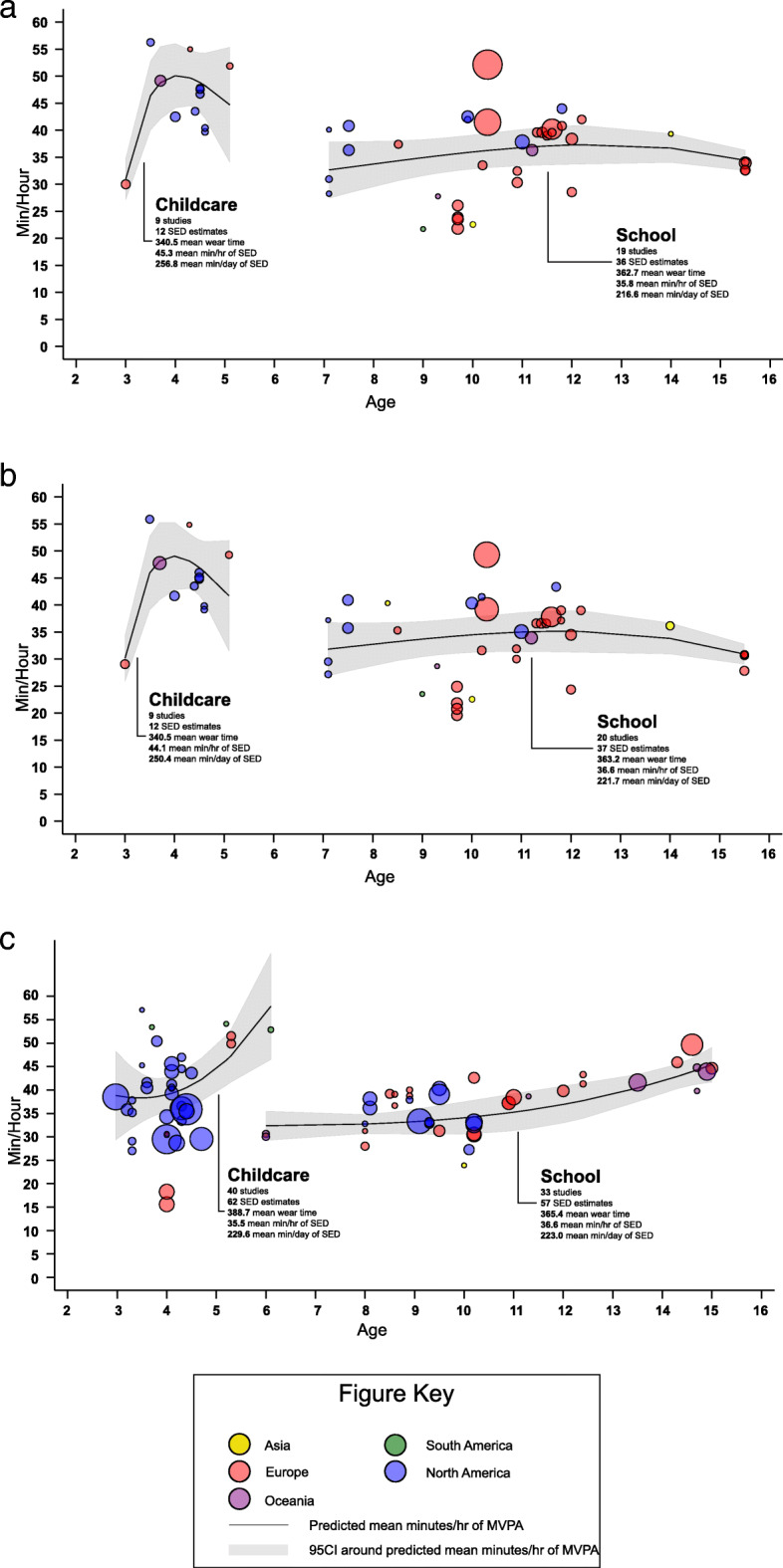
**a**-**c**. Scatter plot and estimated mean min/hour of sedentary time by age during childcare and school. **a** = Girls – studies that provide girl specific estimates of ST. **b** = Boys – studies that provide boy specific estimates of ST. **c** = Total – studies that combine boys and girls together estimates of ST

**Table 2:** Summary of adjusted pooled mean in minutes and min/hour estimates of physical activity and sedentary time by structured settings

Settings	Outcomes	k	e	Adjusted pooled mean (min)	SE	Lower	Upper	Adjusted pooled mean (min/h)	SE	Lower	Upper
Childcare	Sedentary time	44	86	221.8	9.7	196.1	247.5	36.7	2.0	32.5	40.9
	Light physical activity	30	64	82.0	5.0	70.8	93.2	13.5	1.0	11.1	15.4
	Moderate physical activity	13	24	19.7	4.1	8.1	31.1	3.6	0.8	1.4	5.9
	Vigorous physical activity	17	36	12.6	2.2	7.4	17.8	2.6	0.5	1.5	3.8
	Moderate to vigorous physical activity (All)	46	90	32.1	2.3	27.4	36.8	5.1	0.3	4.4	5.9
	Moderate to vigorous physical activity (Pate)	15	32	49.3	3.1	42.0	56.6	7.9	0.6	6.5	9.3
	Moderate to vigorous physical activity (Pfeiffer)	9	19	24.2	2.4	12.9	33.6	3.5	0.4	1.9	5.3
	Moderate to vigorous physical activity (Sirard)	11	22	32.0	8.0	19.5	47.4	5.5	0.9	3.0	7.9
	Moderate to vigorous physical activity (Other)	11	17	28.8	3.0	19.1	38.5	5.1	0.5	3.8	6.4
	Total physical activity	28	42	100.6	5.3	87.6	126.5	13.5	1.0	11.3	15.6
School	Sedentary time	44	130	223.9	4.7	214.	233.6	36.7	0.7	35.1	38.3
	Light physical activity	29	59	114.6	6.1	99.9	129.4	18.8	1.1	16.6	21.6
	Moderate physical activity	16	40	23.3	5.7	9.6	36.9	3.8	0.9	1.6	6.1
	Vigorous physical activity	19	48	10.1	1.2	7.5	12.8	1.6	0.2	1.2	2.1
	Moderate to vigorous physical activity (All)	75	198	27.8	1.5	24.6	31.0	4.4	0.2	3.9	4.9
	Moderate to vigorous physical activity (Evenson)	39	89	24.2	1.2	21.6	26.7	3.9	0.2	3.5	4.3
	Moderate to vigorous physical activity (Other)	36	109	29.9	2.7	24.0	35.6	4.8	0.4	3.9	5.7
	Total physical activity	--	--	--	--	--	--	--	--	--	--
Afterschool	Sedentary time	8	28	54.5	4.4	40.3	68.7	25.9	2.0	19.5	32.3
	Light physical activity	9	21	43.3	3.3	32.6	54.0	21.5	1.7	16.0	26.9
	Moderate physical activity	6	13	10.1	1.6	3.0	17.2	5.1	0.9	1.1	9.1
	Vigorous physical activity	8	22	8.5	1.6	3.4	13.6	4.0	0.8	1.4	6.7
	Moderate to vigorous physical activity	14	31	23.5	1.9	19.1	27.9	11.7	1.1	9.2	14.1
	Total physical activity	--	--	--	--	--	--	--	--	--	--
Sport / Physical Activity programs	Sedentary time	16	38	11.7	1.5	8.3	15.2	11.4	1.7	7.5	15.3
	Light physical activity	13	34	25.6	2.2	20.4	30.8	26.3	2.1	21.5	31.2
	Moderate physical activity	12	33	12.8	1.3	10.6	17.0	12.3	1.4	9.8	16.7
	Vigorous physical activity	13	33	8.5	2.2	4.4	14.6	7.3	1.9	3.9	12.7
	Moderate to vigorous physical activity (All)	18	40	18.9	2.4	13.6	24.2	20.9	2.3	15.9	25.9
	Moderate to vigorous physical activity (Evenson)	9	16	18.8	3.5	11.1	26.4	18.5	2.7	12.4	24.6
	Moderate to vigorous physical activity (Freedson)	7	18	22.4	5.0	11.4	33.5	21.0	3.8	12.7	29.4
	Total physical activity	--	--	--	--	--	--	--	--	--	--

### Childcare

A total of 60 studies [11, 13, 34–91] conducted in 12 different countries from four global regions (i.e. North America, Europe, Oceania and South America) representing 14,763 children (mean age = 4.2 years old) were identified and included. The most common design was cross-sectional [13, 34–78] and randomized controlled trials (RCT) [79–89]. The mean wear time was 371.4 minutes (approx. 6:11 hours) during childcare hours. For the majority of the studies (*n*=33) [35, 36, 41, 42, 47–50, 54, 56, 58, 59, 61–69, 73, 75–78, 81, 86, 88–90] PA and/or ST were measured during attendance at childcare only, while 17 measured all waking time [34, 37, 38, 46, 52, 53, 55, 57, 59, 70, 71, 74, 79, 80, 85, 91], 3 studies measured PA and/or ST using 24 hour protocols [39, 45, 51], and 7 did not specifically report wear protocol [13, 38, 40, 43, 82–84]. A total of 75% of the studies used Actigraph accelerometers and 23% used the Actical accelerometers. A variety of cut-points were used with the most frequently used being Pate (2004, 2006) [34, 37, 40, 50–52, 55, 57, 59, 62, 64, 67, 78, 82–86], Sirard (2001, 2005) [13, 36, 38, 43, 49, 53, 56, 60, 61, 65, 68], Pfeiffer (2006) [39, 41, 66, 69–71], Van Cauwenberghe (2011) [36, 38, 58, 63, 72, 74, 88], and Evenson (2008) [44, 52, 54, 73, 78].

The meta-analyses indicated that children spend about 221.8 minutes or 36.7 min/hour of their time in ST and 32.1 minutes or 5.1 min/hour in MVPA (all cut-points) during attendance. Difference on estimates were found comparing the cut-points, where higher estimates was found on Pate cut-point (49.3 Minutes or 7.9 min/hour) and the lowest on Pfeiffer cut-point (24.2 minutes or 3.5 min/hour) (Table 2). The meta-regression analysis have indicated that 9 of 12 models were significant and the explained variance ranged between 18.2% (ST min/hour estimate) to 60.6% (ST minute estimate). The direction and magnitude varied between outcomes and variables (Table 3s.).

Figures 2a-c and Fig. 3a-c indicate the estimated ST and MVPA min/hour by age from studies that provide the mean wear time during childcare. Estimates of MVPA min/hour decreased as children age in studies that provide girl and boy specific estimates. For boys MVPA decreased from 9 min/hour at age 3 to 5 min/hour at age 5, while for girls MVPA decreased from 7 min/hour to 4 min/hour. However, an inverted “U” pattern was observed for studies that combined estimates of MVPA for boys and girls. For studies that provide ST estimates for girls and boys separately, increases in ST from 3 to 4 years old and then a leveling off between 4 and 5 years old was observed. Studies that combine boys and girls show that ST increased steadily as children aged.

### School

A total of 91 studies [91–181] conducted in 29 countries from all global regions representing 42,463 youth (mean age = 10.1 ± 2.1) were included. Although all global regions are represented, only one study from South America [92] and five from Asia were found [93–97], while Europe [91, 97–134] (45.9%) and North America [135–177] are the most represented regions. Similar to childcare, cross-sectional, and RCT [112, 124, 144, 145, 157, 167, 168, 174–177] were the most common design. The mean wear time was 362.8 minutes (approx.:6:04 hours) during school hours. In 50 studies, the measure of PA and/or ST occurred during all waking time [91, 95, 96, 98, 99, 103–111, 113, 115–118, 120–126, 128, 130–134, 140, 141, 143, 150, 151, 155, 160, 161, 163, 168, 171, 176, 178–180] while in 20 studies during school hours were measured [92, 93, 97, 114, 136–139, 152, 153, 156, 159, 164–167, 172, 174], 11 studies measured using 24-hour protocols [101, 119, 127, 154, 159, 169, 170, 175, 181], and 11 did not specifically report wear protocols [94, 102, 104, 112, 135, 142, 145, 148, 149, 157, 173]. A total of 86.9% of the studies used Actigraph accelerometers, and the most often used cut-points were Evenson (2008) [92, 96, 98–100, 103, 114, 115, 121, 123, 124, 126, 129, 130, 136–139, 141–143, 153, 154, 156, 157, 159, 164–167, 173–177], Freedson (1998, 2005) [93, 94, 140, 144, 146, 151, 152, 168], Puyau (2002, 2004) [105, 108, 117, 145, 178, 180], and Treuth (2004) [122, 128, 162, 170].

The meta-analyses indicated that youth spend about 223.9 minutes or 36.7 min/hour of their time in ST and 27.8 minutes or 4.4 min/hour in MVPA (all cut-points) or 24.8 minutes and 3.9 min/hour considering Evenson cut-point during attendance. The meta-regression analysis have indicated that 4 of 10 models were significant and the explained variance ranged between 6.0% (MVPA min/hour estimate) to 43.8% (ST minute estimate). The direction and magnitude varied between outcomes (Table 4s.).

Figures 2a-c and Fig. 3a-c indicate the estimated hourly ST and MVPA by age from studies that provide the mean wear time during school. Studies that provide boys and girls estimates separately demonstrated a similar pattern of MVPA accumulation across ages. At age 5 boys and girls spent approximately 2 min/hour in MVPA during school, this increased to approximately 5 min/hour (girls) and 7 min/hour (boys) by age 8. Between 8 and 16 years a slight decreased in MVPA min/hour was observed. Alternatively, for studies that combined boys and girls MVPA estimates MVPA min/hour was relatively stable from 6 to 15 years. For ST the flat line were observed in studies that provide estimates for each sex, while and increasing of min/hour in ST (30 minutes to 40 minutes) when aging among studies that combine sex.

### Afterschool programs

The 14 included studies [17, 149, 156, 182–193] were conducted in only two countries, with the majority taking place in United States and the one other study conducted in Australia. The total sample size for all studies combined was 12,021 children (range 82 to 2,053, mean age = 8.3 years). Five studies had more than 600 children [17, 182–185]. Cross-sectional studies represented the most common design [17, 156, 183, 184, 186–192], and Actigraph was the only accelerometer brand used to measure PA and ST. The majority of the studies addressed the outcomes during afterschool hours only, while one considered all waking hours in addition to activity and sedentary during afterschool hours. The mean valid wear time during attendance was 125.7 minutes per day (approx. 2:05 hours). A variety of cut-points were used with the most frequent being Freedson (2005) [189, 191, 193], Evenson (2008), and Matthews (2008) [17, 183, 185, 188].

The meta-analyses estimates indicated that youth spend about 54.5 minutes or 25.9 min/hour of their time in ST and 23.5 minutes or 11.7 min/hour in MVPA during attendance. Only 3 of 10 models of meta-regression were significant and explained 10.6% (ST min/hour estimate) to 52.7% (LPA minute estimate) of the variance (Table 5s).

### Summer camp

All studies (*n* = 4) were conducted in the United States [31, 194–196], and three of four were published between 2017 and 2018 [31, 195, 196], using cross-sectional designs [31, 194–196] or nested quasi-experimental [31], and Actigraph accelerometers [31, 194–196]. The total sample size for all studies combined was 3,330 children (range 132 to 3,389, mean age = 8.8 years). All four included studies reported MVPA during summer camp hours, while only one reported ST [31] and one reported VPA [196]. The mean valid wear time during attendance was 409.0 minutes per day (approx. 6:49 hours). One study estimated MVPA using five different cut-points and the means ranged between 18.8 minutes to 50.4 minutes, or 3.3 min/hour to 9.0 min/hour [194].

### Physical activity / Sport programs

A total of 18 studies [151, 197–213] from five different countries in three regions (i.e. North America, Europe and Oceania) were identified and included, representing 2,293 participants. The total valid wear time was 67.8 minutes (1:07 hour). Only one study used RCT design [197], and all other were cross-sectional [151, 198–213]. Actigraph was the lone accelerometer brand, and the most often used cut-points were Evenson (2008) [198–200, 204, 206, 208, 210], and Freedson (2005) [151, 201, 203–205, 207, 211, 213]. The meta-analyses estimates indicated that youth spend about 11.7 minutes or 11.4 min/hour of their time in ST and 18.9 minutes or 20.9 min/hour in MVPA (all cut-point) during attendance. The meta-regression analyses revealed that 8 of 10 models were significant and explained 26.4% (ST min/hour estimate) to 68.1% (LPA minute estimate) of the variance (Table 6s).

## Discussion

The purpose of this systematic review and meta-analyses was to estimate the amount of PA and ST that youth accumulate during different structured settings (i.e., childcare, school, afterschool programs, summer camps and sports programs). The present study provides absolute (i.e. minutes) and relative (i.e. min/hour) estimates of PA and ST from a large sample of studies that represent different countries and regions across the world. A key finding of this study is that all structured settings provided substantial amounts of physical activity during attendance. These findings highlight the important contribution structured settings have on youth accumulation of health-enhancing physical activity.

Historically school-based settings have been the preferred environment for promoting PA and reducing ST opportunities for children and adolescents [213–215]. As expected, the majority of the studies were conducted in school and childcare, in high income-countries, and reported MVPA. However, in recent years, the number of studies focusing on other structured settings (i.e. afterschool, summer camps and sport programs), and reporting ST and additional metrics of PA beyond MVPA during attendance has increased. This is important for two reasons. First, this indicates the relevance and importance of other structured settings for promoting PA for children and adolescents across different times of the week such as weekdays (i.e. afterschool and sports programs), and weekend days (i.e. sports programs), and during times away from school (i.e. summer camp, holiday camps). Second, the estimates of ST and PA intensity provide a better understanding of the overall ‘contribution’ of these structured settings to youths’ ST and PA. This is valuable information for researchers and practitioners for further initiatives, programs, and policies.

Globally, the school-time estimates presented herein (~ 27.8 min/day MVPA) are slightly below the recommendation that states youth should accumulate at least 30 minutes of MVPA during attendance [19, 20]. Considering these are mean estimates only, and despite the high methodological variability and cultural differences between studies, these data provide evidence that schools are close to providing the expected amount of MVPA during attendance. However, this does not mean that schools are reaching their potential for promoting PA. For example, in the United States, fewer public schools have adopted all components of the comprehensive school PA program [216], which calls for multiple school-related environments (e.g., before and after school time) to provide comprehensive and consistent PA programming and opportunities for youth. Moreover, the current data from the Global Matrix 3.0 indicates that 40.8% of the 49 included countries were graded C or D on school indicators for promoting PA opportunities [217]. Important to note that the observed heterogeneity was partially explained by the moderators. For MVPA estimates for example, while the variability are explained by the sex and the global region context, other variables related to the methods, such as cut-points and risk of bias are significant in the models (i.e. absolute and relative estimates) as well.

In childcare, the current study showed TPA per hour estimates were close the 15 min/hour Institute of Medicine (IOM) recommendation [218], and children accumulate 55% of the daily recommended MVPA while attending (~ 6 hours). Once again, this finding does not mean that all childcare settings are automatically achieving all PA recommendations. For example, childcare settings are called upon to provide a variety of indoor/ outdoor [219–222], structured/organized activities [219–221], and to eliminate sitting for extended periods [219]. To date, all released guidelines for pre-school aged children [219–222] provide critical elements on PA for policy makers, educators, and childcare service, however, little is known about countries regulations, and the dissemination and implementation of any initiative in that direction maybe restricted to a few countries. For childcare, the more consistent moderator in the meta-regression was the accelerometer brand (i.e. Actigraph and Actical), which was significant for the absolute and relative metrics estimates models of ST, LPA, MVPA and TPA.

The current study indicates that the mean estimate of MVPA in afterschool settings was 23.5 min/day, 6.5 minutes short of the 30 minutes/day recommendation for afterschool program hours [223]. Nonetheless, afterschool programs provide children a substantial amount of MVPA. Moreover, during attendance youth spend less time sedentary (< 45% of the time), compared to childcare and school (~ 60%). In other words, current practice in afterschool programs provides children and adolescents with substantial amounts of PA and limits ST. Thus, afterschool programs have great potential to promote youth PA and reduce ST. Simply providing children and adolescents access to these programs may provide substantial amounts of MVPA and reduce ST.

Estimates indicated that youth accumulate 18.9 minutes in MVPA, with 8.5 minutes of this spent in VPA during PA / sport programs. Interestingly, for boy and girl estimates only, the amount of MVPA accumulated during the sessions were 28.3 and 26.4 minutes, respectively. The difference between the combined estimates versus separate boys and girl estimates are due to the type of activity. While the majority of the included studies for the combined estimate have pre-planned PA or dance [198, 199, 207, 209, 211] for boy and girl estimates included more sport activities such as soccer, basketball, and flag ball. Regardless the type of planned activity, the amount of MVPA accumulated in a lower length of time (~ 60 minutes), is substantial. In addition, children accumulate more daily MVPA during sports day compared non-sport day, and reduced ST by nearly 40 minutes [224].

The out-school months (e.g. vacation) has been identified as critical period associated to negative effects on youth´s health due the less structured environment (e.g. lack of routine, non-supervision) that they are exposure. However, few studies examined youth´s accumulation of ST and PA during summer camp programs and all included studies are from the United States [31, 194–196]. While studies indicated that youth are close to [195] or achieving more than the daily recommendations of MVPA [31, 196], the other indicated similar estimates when compared to a school day [194]. Thus, more studies conducted in summer camps are necessary to estimate their potential for promoting physical activity.

The major limitation is the high variability between the studies in all outcomes and structured settings (i.e. 40.6% of the included studies had low score on the risk of bias tools, and I^2^ > 54.4%). Previous systematic reviews [22, 23] have reported several methodological reasons that explain the variability between studies, such as differences in accelerometer cut-points, study design, inclusion criteria, measurement protocol of PA and ST, and data reduction processes. Additionally, estimates may be influenced by educational policies, length of attendance, delivery based (e.g. public/private, and church-based, school-based, family-based), and type of sport programs (e.g. competition, practice, and leisure activity), and/or cultural differences. Further studies should include descriptive information related to the structured (e.g. child attendance, start time and end time, school length duration), accelerometer protocol measure, and descriptive information about the valid data (e.g. mean wear-time, mean of valid days). Finally, the present study did not review the grey literature.

The present study also has several strengths that should be highlighted including: (a) estimated PA and ST for several well attended settings; (b) all studies provide objectively-measured estimates of PA and/or ST during structured setting attendance; (c) exhaustive literature search representing countries around the world. The estimates provided herein could support further policies and recommendations for PA and ST, and help to identify potential levers for intervention in structured settings across the world.

## Conclusions

The present study summarized the amount of PA (i.e. light, moderate, vigorous, MVPA and total PA) and ST that youth accumulate while attending childcare, school, afterschool programs, summer camp and PA/sport programs. The majority of the included studies are conducted in childcare and school, and in high-income countries. Our study found that routine practice in childcare and school provide children with large quantities of PA. These findings demonstrate that interventions delivered during the childcare and school day might produce better results if they focus on reducing sedentary time rather than promoting PA. Further, future PA interventions may need to target times outside of the school and childcare day. In light of these findings, governments and public health agencies should focus efforts on providing all youth access to these structured settings for health benefit.

## Supplementary Information

**Additional file 1.**

**Additional file 2.**

**Additional file 3.**

**Additional file 4.**

**Additional file 5.**

**Additional file 6.**

## Data Availability

All data generated or analyzed during this study are included in this published article.
