# Supplementary material for: Physical activity and sedentary time of youth in structured settings: a systematic review and meta-analysis
Source: Int J Behav Nutr Phys Act. 2020 Dec 4;17:160. doi: 10.1186/s12966-020-01054-y (PMC7716454; doi:10.1186/s12966-020-01054-y)
Supplement: Supplementary file 2 — Additional file 2. [file 12966_2020_1054_MOESM2_ESM.docx]

Additional table 1. Searching terms.

| Group | Search terms | Search strategy | | Search |
| --- | --- | --- | --- | --- |
| Outcome | Physical activity, Sedentary | #1 | **TITLE: “**physical activity” **OR** **ABSTRACT** “physical activity”  **TITLE:** sedentary **OR** ABSTRACT sedentary | # 1 **AND** #2 **AND** #3 **AND** #4 **AND** #5 |
|  |  | #2 |  |  |
| Structured setting | afterschool, after-school, sport, school, summer camp, summer, intervention, childcare, preschool, program | #3 | afterschool **OR** “after-school” **OR** sport **OR** school **OR** “summer camp” **OR** summer **OR** intervention **OR** childcare **OR** preschool **OR** program |  |
| Measure | objective measure, accelerometer | #4 | objective measure **OR** accelerometer |  |
| Population | children, adolescent | #5 | child* OR adolesc* |  |
