# Supplementary material for: Physical activity and sedentary time of youth in structured settings: a systematic review and meta-analysis
Source: Int J Behav Nutr Phys Act. 2020 Dec 4;17:160. doi: 10.1186/s12966-020-01054-y (PMC7716454; doi:10.1186/s12966-020-01054-y)
Supplement: Supplementary file 3 — Additional file 3. [file 12966_2020_1054_MOESM3_ESM.docx]

| **Risk of bias criteria** | **Answer** | | |
| --- | --- | --- | --- |
| **Study: _______________________________________________** | **Yes**  **(2)** | **Yes, partially**  **(1)** | **No (0)** |
| **The study presented and adequately described…** |  |  |  |
| … the design (i.e. cross-sectional, RCT, longitudinal).  (**Yes** = presented; **Yes, partially** = presented, but not adequately described; **No** = Not reported) |  |  |  |
| … the number of settings, classrooms and sample size.  (**Yes** = presented all information; **Yes, partially** = presented some information; **No** = Not reported) |  |  |  |
| …the demographic characteristics of the structured setting (i.e. geographical location, and structured setting hours).  (**Yes** = presented all information; **Yes, partially** = presented some information; **No** = Not reported) |  |  |  |
| …the sample characteristics (i.e., sex, age, socio-economic status, and racial/cultural background).  (**Yes** = presented all information; **Yes, partially** = presented some information; **No** = Not reported) |  |  |  |
| …the brand and model of objective-measured device and wear location.  (**Yes** = presented all information; **Yes, partially** = presented some information; **No** = Not reported) |  |  |  |
| …the physical activity/ sedentary time objective-measured protocol (i.e. Number of days and hours using).  (**Yes** = presented all information; **Yes, partially** = presented some information; **No** = Not reported) |  |  |  |
| …the physical activity/ sedentary time protocol measured comprising the structured setting hours.  (**Yes** = start time and end time; **Yes, partially** = waking time or 24 hours then reduced to setting hours; **No** = Not reported) |  |  |  |
| … the valid days and valid hours criteria.  (**Yes** = presented all information; **Yes, partially** = presented some information; **No** = Not reported) |  |  |  |
| …the non-wear time criteria and epoch length.  (**Yes** = presented all information; **Yes, partially** = presented some information; **No** = Not reported) |  |  |  |
| …the study presented the cut-point for physical activity/ sedentary time.  (**Yes** = presented all information; **Yes, partially** = presented some information; **No** = Not reported) |  |  |  |
| …the average of valid wear-time during setting.  (**Yes** = presented all information; **Yes, partially** = presented some information; **No** = Not reported) |  |  |  |
