# Supplementary material for: Physical activity and sedentary time of youth in structured settings: a systematic review and meta-analysis
Source: Int J Behav Nutr Phys Act. 2020 Dec 4;17:160. doi: 10.1186/s12966-020-01054-y (PMC7716454; doi:10.1186/s12966-020-01054-y)
Supplement: Supplementary file 4 — Additional file 4. [file 12966_2020_1054_MOESM4_ESM.docx]

Table 1s. Descriptive characteristics and risk of bias of the included studies.

| First Author  (Year) | Country | Structured  Setting | Setting hours | Study Design | Sample size  (Highest to lowest) | Sample characteristics  (age, sex, socioeconomic status, race) | Risk of bias |
| --- | --- | --- | --- | --- | --- | --- | --- |
| Addy  (2014) | U.S. | Childcare | NR | Cross-sectional | 22 preschools;  n = 199 | 4.2 years;  50.2% girls;  NR;  47.7% Black, 40.7% White, and 11.6% other | 20 |
| Alhassan  (2007) | U.S. | Childcare | 8 am – 5 pm | RCT | 1 Head Start preschool program;  n = 32 | 3.5 years;  37.5% girls;  Low income;  Latino and African-American | 21 |
| Alhassan  (2012) | U.S. | Childcare | 8:30 am – 4:30 pm | RCT  (pilot) | 2 preschool centers;  8 Classrooms;  n = 71 | 4.3 years;  51% girls;  Low income;  61% Latino/Hispanic, and 39% African-American | 22 |
| Alhassan  (2016) | U.S. | Childcare | 7 am – 4:30 pm | RCT | 10 preschool centers;  36 classrooms;  n = 291 | 4.1 years;  50% girls;  NR;  41% Hispanic, and 25% African-American | 22 |
| Andersen  (2017) | Norway | Childcare | NR | Cross-sectional (nested RCT) | 1 school;  n = 111 | 3.7 years;  51.3% girls;  NR;  NR | 19 |
| Annesi  (2013a) | U.S. | Childcare | 9:15 am – 2 pm | RCT | YMCA-affiliated preschools;  98 classrooms;  n = 885 | 4.4 years;  51.2% girls;  NR;  86% african-american, 9% hispanic, 3% white, and 2% other | 14 |
| Annesi  (2013b) | U.S. | Childcare | 9:15 am – 2 pm | RCT | 7 preschool;  19 classrooms;  n = 338 | 4.6 years;  53.8% girls;  NR;  92% African-American | 16 |
| Annesi  (2013c) | U.S. | Childcare | 9:15 am – 2 pm | RCT | YMCA-affiliated preschools;  32 classrooms  n = 275 | 4.6 years;  55.6% girls;  Lower and lower to middle income;  African-American | 16 |
| Barbosa  (2016) | Brazil | Childcare | 8 am – 5 pm  8 am – 12 pm  2 pm – 6 pm | Cross-sectional | 8 centers of early childhood education;  22 classrooms;  n =370 | 4 to 6 years;  49.6% girls;  24.5% of mother with ≥8 years of education;  NR; | 21 |
| Bonis  (2014) | U.S. | Childcare | 8:30 am – 3 pm | RCT | 26 childcare;  n = 209 | 3.8 years;  50% girls;  NR;  NR | 22 |
| Byun  (2013) | U.S. | Childcare | NR | Cross-sectional | 17 preschools (9 Montessori and 8 traditional);  n = 331 | 4.4 years;  49% girls;  46.0% of parents with ≤ college degree;  35% African-American, 51% White; and 14% other | 18 |
| Byun  (2015) | U.S. | Childcare | NR | Cross-sectional | 22 preschools (11 commercial, 7 religious, 4 Head Start);  n = 191 | 4.1 years;  50.3% girls;  NR;  45.6% African-American, 42.6% White, and 11.5% other | 18 |
| Carson  (2015) | Canada | Childcare | NR | Longitudinal | 12 childcare centers;  n = 86 | 3.3 years;  50% girls;  9.3% of parents with education between 9th-12th grade;  NR | 20 |
| Carson  (2016) | Australia | Childcare | NR | Longitudinal | 6 local government areas (2 low income, 2 middle income, 2 High income)  65 Centers;  n = 177 | 4.2 years;  43.5% girls;  57.1% of parents with university degree;  NR | 19 |
| Copeland  (2016) | U.S. | Childcare | NR | Cross-sectional | 30 childcare centers;  60 classrooms;  n = 388 | 4.3 years;  51% girls;  61% with annual family income ≤ $50,000;  43% White, 40% African-American; and 17% other | 20 |
| Dawson-Hahn  (2015) | U.S. | Childcare | 9 am – 1 pm | Cross-sectional | 4 Head Start program;  n = 82 | 4.7 years;  42.0% girls;  70.4% of parents with less than college education;  Latino | 17 |
| Delaney  (2014) | U.S. | Childcare | NR | Cross-sectional | 24 licensed family child care homes;  n = 144 | 4.0 years;  NR;  NR;  NR | 19 |
| Ellis  (2017) | Australia | Childcare | NR | Cross-sectional | 11 childcare centers  (5 middle/ high SES, 6 low SES);  n = 301 | 3.7 years;  52% girls;  52% low income;  NR | 16 |
| Erinosho  (2016) | U.S. | Childcare | NR | Cross-sectional | 50 childcare centers;  73 classrooms;  n = 544 | 3 to 5 years;  50% girls;  NR;  62% White, and 26% Black/African-American | 21 |
| Gagné  (2013) | Canada | Childcare | NR | Cross-sectional | 20 childcare centers;  n = 242 | 3 to 5;  49.2% girls;  NR;  NR; | 20 |
| Goldfield  (2016) | Canada | Childcare | 8:30 am – 4:30 pm | RCT | 6 childcare centers;  n = 83 | 3.3 years;  50.6% girls;  62.5% with annual family income ≥ $ 50,000;  NR | 19 |
| Henderson  (2015) | U.S. | Childcare | NR | Cross-sectional | 35 licensed full and partial-day Connecticut  childcare centers;  n = 389 | 4.7 years;  50% girls;  Low-income;  50% Hispanic, and 34% Black | 19 |
| Hesketh  (2014) | England | Childcare | 6 am – Noon  or  Noon – 5 pm | Cross-sectional | n = 593 | 4.1 years;  51% girls;  32% of mother left education at 16 years or less;  3% Non-white | 18 |
| Hinkley  (2016) | Australia | Childcare | NR | Cross-sectional | 71 childcare centers and 65 preschools;  n = 731 | 4.6 years;  46% girls;  NR;  NR | 15 |
| Kuzik  (2016) | Canada | Childcare | NR | Cross-sectional  (nested – Quasi-experimental) | 8 childcare centers;  n = 100 | 3.2 years;  47% girls;  49% had a parent with a university education;  NR | 20 |
| LaRowe  (2016) | U.S. | Childcare | NR | Cross-sectional | 20 early childcare and education (7 family 7 small centers and 6 large centers);  n = 231 | 2 to 5 years;  NR;  NR;  73.3% White/Caucasian, 8.9% African-American, 8% American-Indian, and 2.1% Multiple races | 17 |
| Loprinzi  (2010) | Australia | Childcare | NR | Cross-sectional | 13 childhood  education centers (5 low SES, 5 middle SES, 3 High SES);  n = 156 | 3.7 years;  48.1% girls;  31.4% of parents with high school or less;  NR | 19 |
| Mazzucca  (2018) | U.S. | Childcare | 8:30 am – 5:30 pm | Cross-sectional | 49 early care and education centers;  n = 558 | 3 – 5 years;  50% girls;  NR;  57% White, 31% Black, 8% Hispanic, and 4% other | 19 |
| Møller  (2017) | Denmark | Childcare | 8:32 am – 3:31 pm | Cross-sectional | n = 231 | 3.0 years;  50.6% girls;  NR;  NR | 20 |
| Neshteruk  (2018) | U.S. | Childcare | NR | Cross-sectional | 26 counties in central North Carolina;  166 family childcare homes;  n = 496 | 2.97 years;  50.4% girls;  NR;  63.3% Black or African-American; 27.2% White, 8.5% Mixed race, 4% Hispanic or Latino, and 1% other | 20 |
| Nielsen  (2012) | Denmark | Childcare | 8 am – 1 pm or 2 pm | Longitudinal | 18 schools;  n = 594 | 6.3 years;  47.8% girls;  NR;  88.5 % Danish | 19 |
| Olesen  (2013) | Denmark | Childcare | NR | Cross-sectional | 23 preschools;  n = 426 | 5.8 years;  50.5% girls;  NR;  NR | 21 |
| O’Dwyer  (2014) | England | Childcare | 9 am – 3 pm | Cross-sectional | 12 preschools in a high social and economic deprivation;  n = 188 | 4.6 years;  46.8% girls;  NR;  NR | 19 |
| O’Neill  (2016) | U.S. | Childcare | NR | Cross-sectional | 16 public and private preschools;  n = 341 | 4.6 years;  46.8% girls;  NR;  46% African-American, 37% White, and 17% others | 18 |
| Pagels  (2010) | Sweden  U.S. | Childcare | NR | Cross-sectional | 4 schools (2 in Sweden and 2 in U.S);  n = 55 | 4.5 years;  49% girls;  NR;  NR | 17 |
| Pate  (2004) | U.S. | Childcare | NR | Cross-sectional | 9 preschools (3 private, 3 Church-based, 3 Head Start);  n = 247 | 4.0 years;  53.4% Girls;  25.5% High school (parent education);  65% Black, 35% White | 17 |
| Pate  (2014) | U.S. | Childcare | NR | Cross-sectional | 9 Montessori and 8 traditional preschools;  n = 301 | 4.5 years;  49.9% Girls;  65.1% of parents with ≥ 4years degree;  NR | 19 |
| Pate  (2016) | U.S. | Childcare | NR | RCT | 16 preschools (8 public and 8 private);  n = 379 | 4.5 years;  49.6% girls;  60.5% of parents with ≥2 years of college/tech school;  44.3% Black, 41.7% White, and 14% other | 21 |
| Razak  (2018) | Australia | Childcare | 9 am – 3 pm | RCT | 10 childcare;  n = 378 | 3.7 years;  46.5% girls;  42.8% of parents with annual income > $80,000;  10% Aboriginal and Torres Island | 20 |
| Rice  (2014) | U.S. | Childcare | NR | Cross-sectional | 47 family day care centers;  n = 114 | 3.7 years;  47.4% girls;  NR;  NR | 20 |
| Schlechter  (2017) | U.S. | Childcare | 7:30 am - NR | Cross-sectional | 2 university sponsored  preschool centers;  8 classrooms;  n = 73 | 3.6 years;  53% girls;  3.1% High school or less (mother education);  80.9% Non-Hispanic Caucasian, and 19.1% Racial/ethnic minority | 20 |
| Schuna  (2016) | U.S. | Childcare | 7 am – 6 pm | Cross-sectional | 4 childcare centers;  n = 62 | 4.3 years;  43.5% girls;  NR;  89.3% Caucasian, 6.7% Mixed-race, 2.7% African-American, 1.3% Hispanic, and 1.3% Native American | 20 |
| Shen  (2012) | U.S. | Childcare | 8:30 am – 4 pm | Cross-sectional | 6 Head Start program;  n = 158 | 3.6 years;  49.3% Girls;  Low income;  NR | 20 |
| Shen  (2013) | U.S. | Childcare | NR | Cross-sectional | 2 preschools;  n = 46 | 4.1 years;  56.5% girls;  NR;  NR | 18 |
| Sisson  (2017) | U.S. | Childcare | 7 am – 5:30 pm | Cross-sectional | 11 tribally affiliated, full-day childcare centers;  n = 82 | 3.8 years;  45% girls;  NR;  67% American-Indian, 28% White, and 5% other race | 21 |
| Soini  (2014) | Finland  (F)  Australia (A) | Childcare | 8 am – 5 pm  (F)  8 am – 3:30 pm  (A) | Cross-sectional | 14 childcare centers  (F)  n = 80  13 childcare centers  (A)  n = 41 | 3.2 years (F);  53% girls% (F);  NR (F);  NR (F)  3.4 years (A);  56% girls (A);  NR (A);  NR (A) | 20 |
| Sugiyama  (2012) | Australia | Childcare | NR | Cross-sectional | 10 childcare in urban areas;  n = 89 | 4.1 years;  46.0% girls;  NR;  NR | 18 |
| Shen  (2015) | U.S. | Childcare | 8 am – 5 pm | Cross-sectional | 10 centers;  n = 98 | 4.5 years;  49% girls;  49% with annual family income ≥ $ 50,000;  70% White, African-American/Black 6%, > 1 race 15%, 6% Asian, or Native-American, and 13% Hispanic | 18 |
| Tucker  (2015) | Canada | Childcare | NR | Cross-sectional | 27 facilities (9 centers, 10 homes, 8 FDK);  n = 218 | 4.1 years;  53.2% Girls;  69.8% with annual family income ≥ $ 60,000;  80.6% Caucasian, 0.3% African-Canadian, 0.7% Aboriginal, 2.0% Arab, 1.0% Latin America, 4.0% Asian, 6.7% other | 21 |
| Tucker  (2017) | Canada | Childcare | NR | RCT | 22 centers-based childcare;  n = 338 | 3.3 years;  48% girls;  62% with annual family income ≥ $ 60,000;  70% Caucasian, 0.8% African-Canadian, 3.9% Aboriginal, 1.6% Arab, 2.4% Latin American, 5.6% Asian, and 15.7% other | 21 |
| Vale  (2009) | Portugal | Childcare | NR | Cross-sectional | n = 59 | 4.3 years;  52.5% girls;  NR;  NR | 17 |
| Van Cauwenberghe (2012a) | Belgium | Childcare | 8 am – 4 pm | Cross-sectional | 4 preschool;  8 classrooms;  n = 107 | 5.1 years;  43.9% girls;  NR;  NR | 18 |
| Van Cauwenberghe (2012b) | New Zealand | Childcare | NR | Cross-sectional | 6 childhood centers;  n = 49 | 4.0 years;  55.1% girls;  NR;  NR; | 19 |
| Van Cauwenberghe  (2013) | Belgium | Childcare | 8 am – 4 pm | Cross-sectional | 26 preschools;  n = 200 | 5.3 years;  43.5% girls;  NR;  NR | 18 |
| Vanderloo  (2014) | Canada | Childcare | NR | Cross-sectional | 5 publicly-funded childcare centers;  n = 31 | 4.1 years;  45.1% girls;  NR;  NR | 20 |
| Vanderloo  (2015a) | Canada | Childcare | NR | Cross-sectional | 9 childcare centers;  n = 101 | 3.5 years;  56.4% girls;  NR;  79.2% Caucasian | 21 |
| Vanderloo  (2015b) | Canada | Childcare | NR | Cross-sectional | 27 facilities (9 centers, 10 homes, 8 FDK);  n = 218 | 4.1 years;  53.2% girls;  53% with annual family income ≥ $ 60,000;  80.6% Caucasian, 0.3% African-Canadian, 0.7% Aboriginal, 2.0% Arab, 1.0% Latin America, 4.0% Asian, and 6.7% other | 20 |
| Vanderloo  (2016) | Canada | Childcare | NR | Cross-sectional | 8 schools from 2 boards;  n = 113 | 4.6 years;  53.9% girls;  NR;  NR | 20 |
| Ward  (2017) | Canada | Childcare | 7:30 am – 5:30 pm | Cross-sectional | 50 childcare centers;  n = 624 | 4.0 years;  47.7% girls;  NR;  NR | 21 |
| Webster  (2015) | U.S. | Childcare | NR | Cross-sectional | 1 Head Start preschool Center;  11 classrooms  n = 118 | 3,8 years;  53.4% girls;  NR;  83.1% African-American, 8.5 Caucasian, 5.1% Hispanic, and 3.4% mixed racial background | 18 |
| Aadland  (2018) | Norway | School | 9 am – 2 pm | Cross-sectional  (Nested-RCT) | Schools (ASK project);  n = 465 | 10.9 years;  52% girls;  NR;  NR | 20 |
| Abbott  (2013) | Australia and  New Zealand | School | NR | Cross-sectional  (Nested-RCT) | n = 53 | 11.2 years;  53% girls,  NR;  NR | 17 |
| Aibar  (2014) | France  (F)  Spain  (S) | School | 8 am – 5 pm  (F)  8:30 am - 2:30 pm  (S) | Cross-sectional | 10 schools  n = 711 | 14.3 years (F);  55.2% girls (F);  6.99 (0-9) SES (F);  NR (F)  14.3 years (S);  45% girls (S);  6.57 (ranged: 0-9) SES (S);  NR (S) | 20 |
| Andersen  (2015) | Denmark | School | 8 am – 2 pm | Longitudinal | 4 schools;  n = 316 | 10 to 14 years;  53.2% girls;  NR;  NR | 18 |
| Baere  (2016) | Belgium | School | NR | Cross-sectional | 30 schools;  n = 201 | 12 years;  51% girls;  NR;  NR | 17 |
| Bailey  (2012) | England | School | NR | Cross-sectional | 11 schools (Happy study);  n = 135 | 11.7 years;  57% girls;  NR;  21.5% Non-white | 17 |
| Bershwinger  (2013) | U.S. | School | NR | Intervention  (pilot) | Rural area in  western New York;  n = 18 | 9.2 years;  38% girls;  Median household income of  $ 35,576;  78% Caucasian | 10 |
| Brittin  (2017) | U.S. | School | NR | Natural-experiment | 2 schools;  n = 53 | 8.5 years,  52.8% girls;  NR;  32% minority | 21 |
| Brusseau  (2016) | U.S. | School | 8 am – 3 pm | Cross-sectional | 3 schools;  n = 395 | 8.4 years;  45% Girls;  93.7% Low-income (school level);  86% Minority (school level) | 20 |
| Burns  (2016) | U.S. | School | 8 am – 3 pm | Cross-sectional | 3 elementary school;  n = 1,049 | 8.4 years;  50.1% girls;  NR;  NR | 18 |
| Burns  (2018) | U.S. | School | 8 am – 3 pm | Cross-sectional | 5 schools;  n = 2,119 | 8.5 years;  49.1% girls;  NR;  NR | 19 |
| Carlin  (2018) | Ireland | School | 8:30 am – 4 pm | Cross-sectional | 6 schools;  n = 199 | 12.4 years;  100% girls;  NR;  NR | 19 |
| Carlson  (2013) | U.S. | School | NR | Cross-sectional | 97 schools;  n = 172 | 10.2 years;  51.7% girls;  NR;  69.2% White non-Hispanic | 15 |
| Carlson  (2015) | U.S. | School | NR | Intervention | 6 district, 24 schools (97 classrooms);  n = 1,322 | 8.8 years;  53.7% girls;  NR;  67.8% Latino | 20 |
| Carlson  (2017) | U.S. | School | NR | Cross-sectional | 317 census block groups in 2 US regions 942 households (subsample);  n = 549 | 14.1 years;  49.9% girls;  64.7% had a parent with a college degree;  31.3% non-White | 18 |
| Carson  (2013) | Australia | School | NR | Longitudinal | 24 schools;  n = 655 | 13.5 years;  100% girls;  4% Had no formal education, 20.2% had grade 10, 15.9% had grade 12, 7.9% had trade/apprentice, 6.3% had a diploma, 6.1% had postgraduate, 25.2% don’t know (mother education);  NR | 18 |
| Carson  (2014) | U.S. | School | NR | Quasi-experimental | 16 schools;  n = 351 | 11.7 years;  55.2% girls;  74.6% Free or reduced lunch;  63.5% non-White | 20 |
| Centeio  (2014) | U.S. | School | NR | RCT | 20 urban elementary schools;  n = 334 | 9.4 years;  57% girls;  NR;  53% African American, 23% Caucasian, 2% Hispanic, and 20% other | 18 |
| Cinemre  (2015) | Turkey | School | NR | Cross-sectional | 1 elementary school;  n = 40 | 8.3 years  0% girls;  NR;  NR | 17 |
| Cohen  (2008) | U.S. | School | 9 am – 2 pm* | RCT | 35 middle schools  (6 cities);  n = 1,566 | 11,8 years;  100% girls;  35.6% reduced or free lunch;  43.1% White, 21.5% African American, 20.2% Hispanic, and 15% other | 12 |
| Costa  (2017) | Brazil | School | NR | Cross-sectional | 5 public schools;  n = 571 | 7 to 12 years;  54.1% Girls;  51.6% of mothers with < 12 years of education;  NR | 16 |
| Cradrock  (2014) | U.S. | School | 8 am – 3 pm | Quasi-experimental | 6 schools;  26 classrooms;  n = 393 | 10.2 years;  52% girls;  NR;  59% Black, 31% Hispanic, 7% Asian, 2% White, and 2% Other | 21 |
| Decelis  (2014a) | Malta | School | 8:30 am – 2 pm | Cross-sectional | 54 schools (nationally representative);  n = 769 | 10.8 years;  51.8% girls;  NR  NR | 17 |
| Decelis  (2014b) | Malta | School | 8 am – 3 pm | Cross-sectional | 20 schools (12 state based, 6 church-based, 2 independent  n= 187 | 11.0 to 12.0 years;  49.7%;  NR;  NR | 19 |
| Engelen  (2013) | Australia | School | 9 am – 3 pm | RCT | 12 primary schools;  n = 206 | 6 years;  46.1% girls;  The Index of Community Socio-Educational Advantage – 1,076 (980-1,170);  parents originating 35 different countries | 22 |
| Fairclough  (2007) | England | School | 9 am – 3 pm | Cross-sectional | 6 schools;  n = 58 | 8.6 years;  46.5% girls;  NR;  NR | 18 |
| Fairclough  (2012) | England | School | 9 am – 3:30 pm | Cross-sectional | 8 primary schools;  n = 223 | 10.7 years;  55.6 girls;  IMD Scores (18.2);  98.9% White British, 0.4% White European, and 0.7% Asian | 20 |
| Farmer  (2017) | New Zealand | School | NR | RCT | 16 schools;  n = 704 | 7.9 years;  51% girls;  NR;  49% New Zealand, 16.8% Maori, 11.5% Pacific, 8.0% Asian, and 14.7% unknown | 18 |
| Gao  (2017) | U.S. | School | 8 am – 3:30 pm | Cross-sectional | 1 school;  n = 138 | 8.1 years;  51.5% girls;  NR;  87% White, 11.6% Hispanic, and1.5% African-American | 15 |
| Goh  (2014) | U.S. | School | NR | Intervention* | 1 elementary school;  n = 219 | 8 to 11;  54.3% girls;  NR;  57% Caucasian/White, 35% Hispanic, 5% Pacific Islander, and 3% other | 12 |
| Guinhouya  (2009) | France | School | 8:30 am – 4 pm | Cross-sectional | 3 elementary school;  n = 93 | 10 years;  48.5% girls;  NR;  NR | 18 |
| Hamer  (2017) | England | School | 9 am – 3 pm | Intervention | 5 Primary and 3 secondary schools;  n = 231 | 8.0 years;  48.4% girls;  NR;  34.6% Caucasian, 18.1% Black, 16.4% Asian, 12.5% Mixed, and 18.1% other | 21 |
| Harding  (2015) | England | School | NR | Longitudinal | 27 secondary school (Peach project);  n = 363 | 12.0 years;  61.4%;  61.7% Low SES;  NR | 18 |
| Harrington  (2016) | England | School | 9 am – 3 pm | Longitudinal | 92 primary schools and 43 secondary schools (Speedy);  n = 301 | 10.2 years;  55.2% girls;  41.2% of parents left full time education at 16 years or less;  NR | 20 |
| Herrick  (2012) | U.S. | School | NR | Quasi-experimental | 6 afterschool programs (school-based);  n = 100 | 10.4 years;  55% girls;  NR;  53% Asian, 31% Latino, 3% White, 2% African-American, and 11% other | 14 |
| Hubbard  (2016) | U.S. | School | NR | Cross-sectional | 13 schools;  n = 453 | 9.1 years;  60.5% girls;  30.9% Free or reduced lunch;  74.4% white, 10.6% Hispanic, 4.9% Black/African-american, 3.3% Multiracial, 2.4% Asian, and 0.9% Native American | 21 |
| Kriemler  (2010) | Switzerland | School | NR | RCT | 15 elementary schools; 28 classrooms;  n = 502 | 6.9 and 11.5 years;  51.2% girls;  9.3% with no formal parental education;  27.0% migrant families | 19 |
| Kim  (2015) | U.S. | School | 7:30 am – 3 pm | Cross-sectional | 1 public elementary school in a low SES neighborhood;  n = 75 | 10.1 years;  61.5% girls;  NR;  54.7% Black, 29.3% Hispanic, and 16% other | 18 |
| Kulik  (2015) | U.S. | School | NR | Cross-sectional | 6 schools;  n = 347 | 9.4 years;  57.3% girls;  NR;  52.3% African-American, 20.6% Caucasian, 3.5% American Indian, 2.3% Hispanic, 1.2% Pacific Islander, and 20.1% other | 15 |
| Kwon  (2015) | U.S. | School | NR | Cross-sectional | 14 public schools;  n = 538 | 10 to 12 years;  53% girls;  NR;  28% White, 8.5% Black, 40.1% Hispanic; and 23.4% No majority race | 20 |
| Lee  (2016) | U.S. | School | 8 am – 3 pm | Cross-sectional | 2 urban schools;  n = 261 | 8.3 years;  51.3% girls;  NR;  67% non-Hispanic | 15 |
| Lewis  (2016) | Australia | School | 9 am – 3 pm | Cross-sectional | 26 schools (21 government-based, 3 independents, and 2 catholic);  n = 491 | 10.8 years;  54.1% girls;  $AU 89,753;  NR | 18 |
| Lin  (2012) | Taiwan | School | 8 am – 4 pm | Cross-sectional | 1 school;  n = 49 | 9.2 years;  0% girls;  NR;  NR | 12 |
| Long  (2013) | U.S. | School | 8 am – 3 pm | Cross-sectional | 2003–2006 NHANES;  n = 2,548 | 7.0 years;  50.3% girls;  28.7% of 130% of the federal poverty level;  58.1% White, 15.8% Black, 14.3% Mexican-American, and 11.7% other | 20 |
| Madsen  (2013) | U.S. | School | NR | Cross-sectional | 7 schools;  n = 156 | 9.8 years;  40% girls;  61% free or reduced price meals;  42% Latino, 32% Asian, 12% African American,  and 14% other | 19 |
| Madsen  (2015) | U.S. | School | NR | RCT | 6 schools;  n = 450 | 9.5 years;  50.8% girls;  NR;  49.4% Latino, 14.9% Multiracial, 9.8% Asian, 6.7% Black, 6.3% White, and 12.9% other | 15 |
| Magnusson  (2011) | Iceland | School | NR | Cross-sectional | 6 elementary schools;  n = 196 | 7.4 years;  55.6% girls;  NR;  NR | 16 |
| Martin  (2017) | Ireland | School | NR | Cross-sectional | 10 schools;  n = 197 | 8.9 years;  50.2% girls;  NR;  NR | 19 |
| Mooses  (2016) | Estonia | School | NR | Cross-sectional | 13 schools;  n = 244 | 9.1 years;  52.7% girls;  NR;  NR | 17 |
| Morton  (2016) | England | School | NR | Longitudinal | 92 primary schools and 43 secondary schools (Speedy);  n = 636 | 10.2 years;  52.3% girls;  18.3% Low, 39.1% Middle, 42.6% High income families;  NR | 19 |
| Nettlefold  (2011) | Canada | School | 9 am – 3 pm | Cross-sectional | 9 elementary schools;  n = 379 | 10 years;  52.1% girls;  NR;  NR | 20 |
| Nielsen  (2012) | Denmark | School | 8 am – 2 pm | Longitudinal | 18 schools;  n = 518 | 9.5 years;  48.6% girls;  NR;  88.6 % Danish | 19 |
| Nilsson  (2009) | Denmark | School | 8 am – 2 pm | Cross-sectional | European Youth Heart Study;  n = 1,184 | 9.7 years;  53% girls;  NR;  NR | 20 |
| Noonan  (2017) | England | School | 9 am – 3pm | Cross-sectional | 6 Schools;  n = 129 | 10.1 years;  61.2% Girls;  NR;  NR | 18 |
| Pau  (2017) | Italy | School | 8:30 am - 1:30 pm | Cross-sectional | 1 school;  n = 169 | 8.6 years;  55% girls;  NR;  NR | 20 |
| Pearce  (2018) | Scotland | School | NR | Cross-sectional | 1 school;  n = 70 | 12.4 years;  67.1% girls;  NR;  NR | 17 |
| Piipari  (2016) | U.S. | School | NR | Cross-sectional | 3 elementary schools;  n = 200 | 7.8 years;  53.5% girls;  NR;  NR | 19 |
| Pizzarro  (2017) | Portugal | School | 7:45 am – 3:30 pm | Cross-sectional (nested - longitudinal) | 9 middle schools;  n = 374 | 11.7 years;  46.2% girls;  NR;  NR | 13 |
| Price  (2013) | U.S. | School | 9 am - 3:15 pm | Cross-sectional | 701 families;  n = 682 | 9.1 years;  49.9% girls;  49% of Household income > $100,000;  67.7% White, 17% Hispanic, 15.3% non-Hispanic non-White | 17 |
| Pulsford  (2013) | England | School | 9 am - 3 pm | Cross-sectional | 7 secondary schools;  n = 629 | 11.0 years;  49.2% girls;  63.9% of household income ≤ £ 40,000;  NR | 20 |
| Rainham  (2012) | Canada | School | NR | Cross-sectional | 6 schools;  n = 316 | 13.3 years;  47% girls;  65.1% with < $50,000 annual household income;  84% Caucasian | 16 |
| Ramirez-Rico  (2014) | Spain | School | Primary  9:30 am – 4 pm  High-School  8:30 am – 2:30 pm | Cross-sectional | 11 schools (4 primary and 7 high-schools);  n = 367 | 12 years;  62.3% girls;  NR;  NR | 19 |
| Resaland  (2016) | Norway | School | 9 am – 2 pm | RCT | 57 schools;  n = 1,063 | 10.2 years;  47.9% girls;  32% upper secondary education (parents);  NR | 19 |
| Ridgers  (2010) | England | School | 7 am – 3 pm | Cross-sectional | 8 schools;  n = 110 | 9.7 years;  59% girls;  NR;  NR | 19 |
| Riley  (2016) | Australia | School | 9 am – 3 pm | RCT | 8 schools;  n = 240 | 11.1 years;  40.9% girls;  NR;  NR | 16 |
| Ross  (2013) | U.S. | School | 9 am – 2 pm | Cross-sectional | 36 middle schools (TAAG);  n = 1,866 | 13.9 years;  100% girls;  NR;  48% White, 22% Hispanic, 18% Black, and 12% Other | 14 |
| Rush  (2012) | New Zealand | School | 9:30 am – 2:30 pm | Cross-sectional | 1 urban school;  2 classrooms;  n = 47 | 9.3 years;  59.5% girls;  Low socioeconomic status;  NR | 18 |
| Sayers  (2012) | U.S. | School | NR | Cross-sectional | 3 elementary schools;  n = 77 | 8.3 years;  48% Girls;  54.5% with annual family income ≥ $60,000;  64.9% Caucasian, 10.3% Asian American, 5.2% Black/African-American, 2.5% American-Indian/Alaskan Native, and 12.9% other | 17 |
| Schneider  (2017) | U.S. | School | NR | RCT | n = 126 | 11.0 years;  52.0% girls;  NR;  48% Latino, 19% non-Latino White, 12% African-American, 10% Asian, 11% other; | 17 |
| Siahpush  (2012) | U.S. | School | 7:30 am – 4 pm | Intervention | 2 schools;  n = 93 | 9.6 years;  46.8% Girls;  NR;  75.9% Caucasian, 14.1% African-American, and 10% other | 15 |
| Silva  (2018) | Portugal | School | 8 am – 5 pm | Intervention | 1 school;  2 classrooms;  n = 49 | 11.7 years;  53% girls;  NR;  96% Caucasian | 20 |
| Sigmund  (2014) | Czech Republic | School | 8 am – 1 pm | Cross-sectional | 6 primary schools;  n = 338 | 9.5 years;  50.3% girls;  NR;  NR | 19 |
| Sprengeler  (2017) | Germany | School | NR | Cross-sectional | 4 schools (2 in middle-income area and 2 in high-income area);  27 classrooms;  n = 207 | 8.5 years;  47.3% girls;  75.5% high SES (ISCED);  NR | 18 |
| Steele  (2010) | England | School | 9 am – 3 pm | Cross-sectional | 92 primary schools (Speedy study);  n = 1,568 | 10.3 years;  55.3% girls;  20.5% parents with high education;  NR | 19 |
| Stewart  (2017) | New Zealand | School | NR | Cross-Sectional | 7 secondary schools;  n = 76 | 14.7 years;  40.8% girls;  NR;  NR | 17 |
| Strugnell  (2016) | Australia | School | 9 am – 3:30 pm | Cross-sectional | 39 schools (subsample of RCT);  n = 298 | 11.2 years;  56.3% girls;  24.6% SEIFA quintile;  13% language other than English | 21 |
| Sutherland  (2017) | Australia | School | NR | RCT | 46 low socioeconomic elementary schools;  n = 989 | 10.1 years;  51% girls;  Low SES;  NR | 18 |
| Suzuki  (2018) | Japan | School | 8:15 am – 2:20 pm  8:25 am – 4 pm | Cross-sectional | 5 primary schools;  11 classrooms;  n = 39 | 10 years;  45% girls;  NR;  NR | 20 |
| Taylor  (2011) | New Zealand | School | 9 am – 3 pm | Cross-sectional | 16 primary schools;  n = 441 | 8 years;  46.7% girls;  NR;  NR | 20 |
| Taylor  (2017) | England | School | 8:15 am – 3 pm | Cross-sectional | 7 primary schools;  n = 186 | 10.2 years;  48.8% girls;  34.6% of children living in income-deprived;  NR | 20 |
| Ting  (2015) | Singapore | School | 7 am – 3 pm | Cross-Sectional | 7 secondary schools;  n = 225 | 14.0 years;  47.6% girls;  NR;  NR | 20 |
| Vanhelst  (2017) | Europe | School | 8:15 am – 2 pm  8:15 am – 4:20 pm | Cross-Sectional | 104 schools in 10 European cities (HELENA study);  n = 2,024 | 14.6 years;  49.8% girls;  33% lower mother education;  NR | 20 |
| Van Sluijs  (2011) | England | School | 8 am – 4 pm | Longitudinal | 92 schools (Speedy study);  n = 1,908 | 10.3 years;  55.9% girls;  NR;  NR | 19 |
| Van Stralen  (2014) | Europe | School | NR | Cross-sectional | Energy Project;  n = 1,025 | 11.6 years;  51% girls;  NR;  75% Native; | 20 |
| Verloigne  (2015) | Belgium | School | 8:30 am – 4 pm | Cross-Sectional | 10 schools;  n = 354 | 10.9 years;  59% girls;  NR;  NR | 17 |
| Weaver  (2016) | U.S. | School | NR | Cross-sectional | 7 schools (2 cities);  24 classrooms;  n = 323 | 7.5 years;  53.5% girls;  62.1% Free and reduced lunch;  33.1% Hispanic, 26.4% Caucasian, 27.8% Black, 9.7% Asian/Pacific/Islander, and 2.5% other | 21 |
| Weaver  (2018a) | U.S. | School | NR | Intervention | 8 elementary schools (rural district);  n = 795 | 7.6 years;  50.5% girls;  91% Free or reduced lunch;  86% African-American | 21 |
| Weaver  (2018b) | U.S. | School | NR | Quasi-experimental | 4 elementary schools;  9 classrooms;  n = 229 | 7.1 years;  58.2% girls;  32.2% Free or reduced lunch;  44.0% Black, 41.1% Caucasian, and 14.9% other | 21 |
| Wells  (2014) | U.S. | School | NR | RCT | 12 schools;  5 regions;  n = 124 | 9.3 years;  56.4% girls;  68.3% Free or reduced lunch;  51.5% White, 30.0 African-American, 8.8% Hispanic, and 9.7% Asian | 19 |
| Wilson  (2017) | Australia | School | 8:55 am – 3:20 pm | Intervention | 1 elementary school;  n = 38 | 12.4 years;  0% girls;  NR;  61% Caucasian, 33% Asian, and 6% Other; | 17 |
| Zimmo  (2017) | Qatar | School | 7 am – 1 pm | Cross-Sectional | 4 primary schools;  8 classrooms;  n = 186 | 5.0 and 9.0 years;  53% girls;  NR;  72.1% Qatari | 21 |
| Arundell  (2013) | Australia | Afterschool | 3:30 pm – 6 pm | Longitudinal | 43 elementary school (18 in low income, 7 in middle income and 18 in high income);  n = 2,053 | 5.5 and 11.0 years;  52% girls;  33% of mother with low education;  NR | 21 |
| Beets  (2010, 2012) | U.S. | Afterschool | 3 pm – 6 pm | Cross-sectional | Single community-based organization;  n = 253 | 8.2 years;  51% girls;  NR;  57% White, 38% African-Americans, and 5% Asian, Hispanic or other | 22 |
| Beets  (2013) | U.S. | Afterschool | 3 pm – 6 pm | Cross-sectional | 18 afterschool programs (3 cities);  n = 785 | 7.7 years;  45% girls;  NR;  67% American-Indian, 28% White, and 5% other race | 22 |
| Beets  (2014) | U.S. | Afterschool | 3 pm – 6 pm  3:30 pm – 6:30 pm | Cross-sectional | 4 large-scale YMCA locations;  n = 1,641 | 7.5 years;  46.5% girls;  NR;  58.0% White, 41% African-American and 1% other | 21 |
| Beets  (2015) | U.S. | Afterschool | 3 pm – 6 pm | Cross-sectional | 19 afterschool programs (10 Community-based, 3 Faith-based, 6 School-based in 2 cities);  n = 812 | 7.7 years;  47% Girls;  NR;  61% White, 39% other | 21 |
| Beets  (2015b) | U.S. | Afterschool | 3 pm – 6 pm | RCT | 12 afterschool program organizations (6 school-based, 1 faith-based, 3 community-based);  n = 1,765 | 8.1 years;  47.6% girls;  NR;  48.4% White, 44.7% African-American, and 6.9% other | 21 |
| Beets  (2018) | U.S. | Afterschool | 3 pm – 6 pm | Cross-sectional  (3 repeated) | 20 YMCAs (25% YMCAs facilities, 64% school-based, 7% community-based 3% faith-based);  n = 3,404 | 7.8 years;  46.3% girls;  13% in poverty;  58.9% White; 34.7% Black, 2.1% Hispanic, and 4.3% other | 22 |
| Cradock  (2016) | U.S. | Afterschool | NR | RCT | 20 afterschool programs;  n = 402 | 7.7 years;  51.2% girls;  NR;  34.8% Hispanic, 30.6% Black, 7.5% White, 3.2% Asian, and 23.9% other | 21 |
| Dzewaltowski (2010) | U.S. | Afterschool | NR | Repeated cross-section | 7 elementary schools;  n = 246 | 9.0 years;  54.8% girls;  54% Free or reduced food;  69.1% White, 20.7% African American, and 10.2% other | 17 |
| Gesell  (2013) | U.S. | Afterschool | 3 pm – 6 pm | Observational | School-based afterschool program (low income);  n = 82 | 7.9 years;  65% girls;  Low income;  NR | 20 |
| Herrick  (2012) | U.S | Afterschool | NR | Quasi-experimental | 6 afterschool programs (school-based);  n = 100 | 10.4 years;  55% girls;  NR;  53% Asian, 31% Latino, 3% White, 2% African-American, and 11% other | 17 |
| Huberty  (2014) | U.S. | Afterschool | NR | Cross-sectional | 9 afterschool programs;  n = 182 | 9.3 years;  100% girls;  NR;  49.4% Black, 28.5% Hispanic, and 22.1% other | 20 |
| Madsen  (2013) | U.S. | Afterschool | NR | Cross-sectional | 7 schools;  n = 156 | 9.8 years;  40% girls;  61% free or reduced lunch;  42% Latino, 32% Asian, 12% African American, and 14% other | 20 |
| Trost  (2008) | U.S. | Afterschool | NR | Cross-sectional | 7 afterschool programs;  n = 140 | 10.1 years;  43.6% girls;  NR;  48.6% White, 17.9% African-American, 10.7% Hispanic, 7.1% Native-American, 0.7% Asian, and 15% NR | 21 |
| Behrens  (2015) | U.S. | Physical Activity / Sport  (Pre-planned PA) | NR | Cross-sectional | 5 elementary schools;  n = 104 | 8 to 12 years;  56.3% girls;  NR;  57.6% White, 25.9 African-American, 11.6% Hispanic, 2.8% Multiple races, 1% Pacific Islander | 17 |
| Behrens  (2016) | U.S. | Physical Activity / Sport  (Pre-planned PA) | NR | Cross-sectional | 4 Low-SES elementary schools;  n = 205 | 8 to 12 years;  NR;  82-89% Free or reduced lunch;  NR | 19 |
| Cain  (2015) | U.S. | Physical Activity / Sport  (Dance) | NR | Cross-sectional | 17 private studios and 4 community centers;  n = 264 | 7.8 years / 13.1 years;  100% girls;  NR;  67.1% White | 16 |
| Cohen  (2014) | U.S. | Physical Activity / Sport  (Soccer) | NR | Cross-sectional | Single community-based parks and recreation program;  5 soccer teams;  n = 29 | 5 to 10 years;  NR;  NR;  NR | 16 |
| Fenton  (2015) | England | Physical Activity / Sport  (Soccer) | NR | Cross-sectional | 49 soccer teams;  n = 109 | 11.9 years;  0% girls;  NR;  NR | 19 |
| Guagliano  (2013) | Australia | Physical Activity / Sport  (Netball, basketball, Soccer) | NR | Cross-sectional | 3 clubs and 10 teams (4 netball, 3 basketball, and 3 soccer);  n = 82 | 13.4 years;  100% girls;  NR;  NR | 18 |
| Guagliano  (2015) | Australia | Physical Activity / Sport (Basketball) | NR | RCT | 2 sport centers;  n = 76 | 10.5 years;  100% girls;  NR;  NR | 18 |
| Guagliano  (2017) | Russia | Physical Activity / Sport (Pre-planned PA) | NR | Cross-sectional | 1 summer camp;  n = 32 | 10.7 years;  100% girls;  NR;  NR | 19 |
| Kim  (2015) | U.S. | Physical Activity / Sport (Pre-planned PA) | 3:30 pm - 4:50 pm | Cross-sectional | 1 public elementary school in a low SES neighborhood;  n = 75 | 10.1 years;  61% girls;  NR;  54.7% Black, 29.3% Hispanic, and 16% other | 19 |
| Leek  (2011) | U.S. | Physical Activity / Sport  (Soccer, Baseball/  Softball) | NR | Cross-sectional | 29 teams (12 girls' teams and 17 boys' teams);  n = 197 | 7 to 14 years;  38% girls;  72.5% with annual family income > U$ 50,000;  72% Hispanic, 17% White and 11% other | 19 |
| Lopez-Castillo  (2015) | U.S. | Physical Activity / Sport  (Dance) | NR | Cross-sectional | 21 studios (17 private and 4 community centers);  n = 291 | 10.4 years;  91% girls;  NR;  69% White | 17 |
| O'Neill  (2012) | U.S | Physical Activity / Sport  (Dance, Jazz, Ballet) | NR | Cross-sectional | 11 dance studios;  n = 137 | 14.6 years;  100% girls;  NR;  80.3% Caucasian, 9.5% African-Americans, and 10.2% other | 20 |
| Ridley  (2018) | Australia | Physical Activity / Sport  (Soccer, Netball, Australia football) | NR | Cross-sectional | 4 local organized sport clubs;  15 teams;  n = 141 | 11.65 years;  49% girls;  Mean socio-economic indexes for areas (1,025) > the national average (1,000);  NR | 21 |
| Rosenkranz  (2011) | U.S. | Physical Activity / Sport (Pre-planned PA) | NR | Cross-sectional | 7 afterschool programs;  n = 240 | 9.3 years;  49% girls;  49% Free or reduced lunch;  62% Caucasian | 22 |
| Sacheck  (2011) | U.S. | Physical Activity / Sport (Soccer) | NR | Cross-sectional | 1 youth indoor recreational soccer;  n = 111 | 9.1 years;  68% girls;  NR;  NR | 18 |
| Schlechter  (2017) | U.S. | Physical Activity / Sport (Flag Football) | NR | Cross-sectional | 14 recreation-level flag football teams;  n = 111 | 7.9 years;  0% girls;  25% Free or reduced lunch;  77.7% Caucasian, and 22.3 % minority | 17 |
| Schuna  (2013) | U.S. | Physical Activity / Sport (Pre-planned PA) | NR | Cross-sectional | 4 elementary schools;  n = 116 | 9.7 years;  55.1% girls;  NR;  NR | 21 |
| Smith  (2016) | Germany | Physical Activity / Sport  (Pre-planned PA) | NR | Cross-sectional | GINIplus and LISAplus;  n = 1,010 | 15.6 years;  50.4% Girls;  71.1% Higher-educated parents (university);  NR | 20 |
| Baker  (2017) | U.S. | Summer camp | 8 am – 4 pm | Cross-sectional | 6 Summer days Camps (one city);  n = 132 | 8.4 years;  44.7% girls;  53% free or reduced lunch;  63.6% black, 33.3% White, and 3.1% NR | 20 |
| Barnett  (2018) | U.S. | Summer camp | NR | Cross-sectional | 1 summer camp;  n = 40  5 summer camps;  n = 142 | 11.5 years;  45% girls;  NR;  43% Black, 18% white, 13% Asian, 10% Hispanic, and 18% Other  7.6 years;  46% girls;  NR;  36% Black, 8% White, 26% Hispanic, and 30% other | 19 |
| Beets  (2011) | U.S. | Summer camp | NR | Cross-sectional | 3 Summer camp (South Carolina, Kentucky and Hawaii);  n = 149 | 8.6 years;  40.2% girls;  NR;  32.8% African-American, and 32.2% White | 18 |
| Weaver  (2017) | U.S. | Summer camp | 8 am – 6 pm | Repeated cross-sectional quasi-experimental | 20 summer day camps (9 organizations);  n = 1,830 | 7.9 year;  45% girls;  NR;  67.7% African-Americans, 25.5% White non-Hispanic, and 6.8% other | 22 |

Legend: U.S = United States of America; NR = Not reported; RCT = Randomized controlled trial; PA = Physical Activity; SES = Socioeconomic status; SEIFA = Socio-Economic Indexes for Areas; ISCED = International Standard Classification of Education.
