# Supplementary material for: Physical activity and sedentary time of youth in structured settings: a systematic review and meta-analysis
Source: Int J Behav Nutr Phys Act. 2020 Dec 4;17:160. doi: 10.1186/s12966-020-01054-y (PMC7716454; doi:10.1186/s12966-020-01054-y)
Supplement: Supplementary file 5 — Additional file 5. [file 12966_2020_1054_MOESM5_ESM.docx]

Table 2s. Descriptive information of the first author, year of publication, objective-measure protocol, sample size, mean and standard deviation of physical activity and sedentary time by structured settings.

| First Author (Year) | Measure | | | | Sample  (n) | Average valid weartime during setting (min) | ST | LPA | MPA | VPA | MVPA | TPA |
| --- | --- | --- | --- | --- | --- | --- | --- | --- | --- | --- | --- | --- |
|  |  |  |  |  |  |  | Mean (SD) | Mean (SD) | Mean (SD) | Mean (SD) | Mean (SD) | Mean (SD) |
|  | Device;  (Wear location) | Number wear Days;  Wear duration | Valid days;  Valid hours;  Non-wear time;  Epoch length | Cutpoint |  |  |  |  |  |  |  |  |
| **Childcare** | | | | | | | | | | | | |
| Addy  (2014) | 7164  (Right hip) | 2 weeks;  waking hours | ≥ 4 weekdays;  ≥ 4.9 hours during school;  ≥ 60min;  15-s | Pate  (2006) | Total  (199) | 492.0 | -- | -- | -- | -- | 61.5  (22.1) | 108.2  (35.2) |
| Alhassan (2007) | Actigraph  (Lower back) | 5 weekdays;  waking hours | ≥ 4 days;  ≥ 10 hours;  ≥ 20 min;  30-s converted to 60-s | Sirard  (2005) | Total A  (15) | 454.7^a^ | 432.6  (19.1) | 23.3  (9.4) | -- | -- | 11.6  (10.2) | -- |
|  |  |  |  |  | Total B  (17) | 479.2^a^ | 436.9  (11.2) | 22.4  (7.9) | -- | -- | 8.4  (5.15) | -- |
| Alhassan (2012) | GT1M  (Lower back) | 7 days;  waking hours | ≥ 4 weekdays;  ≥ 9 hours;  ≥ 20 min;  15-s | Sirard  (2005) | Total A  (48) | 542.9^a^ | 402.5  (30.5) | 92.2 (16.1) | -- | -- | 47.2  (23.8) | -- |
|  |  |  |  |  | Total B  (43) | 558.5^a^ | 437.3  (25.1) | 89.3  (10.9) | -- | -- | 31.8  (14.5) | -- |
| Alhassan (2016) | GT1M  (Lower back) | 5 weekdays;  during childcare hours | ≥ 3 days;  ≥ 7 hours;  NR;  15-s | Sirard  (2005) | Total A  (150) | 416.0 | 316.6  (24.5) | 76.9  (17.0) | -- | -- | 26.2  (14.5) | -- |
|  |  |  |  |  | Total B  (141) | 411.6 | 301.2  (28.4) | 82.3  (18.9) | -- | -- | 28.4  (13.5) | -- |
| Andersen  (2017) | GT1M  /GT3X+  (Left hip) | 5 weekdays;  during childcare hours | ≥ 2 days;  ≥ 6 hours;  ≥ 60 min, with allowance for two  exceptions > zero;  15-s | Butte (2014) | Total  (111) | 486.0 | 262.4  (NR) | 160.3  (NR) | 43.7  (NR) | 9.7  (NR) | 58.0  (20.0) | -- |
| First Author (Year) | Measure | | | | Sample  (n) | Average valid weartime during setting (min) | ST | LPA | MPA | VPA | MVPA | TPA |
|  | Device;  (Wear location) | Number wear Days;  Wear duration | Valid days;  Valid hours;  Non-wear time;  Epoch length | Cutpoint |  |  | Mean (SD) | Mean (SD) | Mean (SD) | Mean (SD) | Mean (SD) | Mean (SD) |
| Annesi  (2013a) | GT3X  (Waist) | NR;  NR | NR;  NR;  NR;  15-s | Pate  (2006) | Total A  (169) | 360.0^a^ | 212.7  (26.2) | -- | -- | 55.8  (12.9) | 81.0  (17.2) | -- |
|  |  |  |  |  | Total B  (716) |  | 215.8  (34.9) | -- | -- | 63.0  (19.8) | 86.0  (23.7) | -- |
| Annesi  (2013b) | GT3X  (Waist left) | NR;  NR | NR;  NR;  NR;  15-s | Pate (2006) | Total A  (136) | 285.0 | 138.5  (31.6) | -- | -- | 20.5 (5.8) | 91.4 (23.6) | -- |
|  |  |  |  |  | Total B  (202) |  | 139.0  (30.7) | -- | -- | 20.1 (5.7) | 90.3 (22.2) | -- |
| Annesi  (2013c) | GT3X  (Waist) | NR;  NR | NR;  NR;  NR;  15-s | Pate (2004)  Pate (2006) | Total A  (121) | 285.0 | 143.9  (13.6) | -- | -- | 18.2  (0.9) | 86.3  (5.6) | -- |
|  |  |  |  |  | Total B (154) |  | 139.9 (16.5) | -- | -- | 20.1 (1.3) | 90.6  (7.5) | -- |
| Barbosa  (2016) | GT3X  (Left waist) | 5 weekdays;  during childcare hours | ≥ 3 days;  ≥ 6 hours (A,B)  and ≥ 2 hours (C)  NR;  1-s | Sirard (2005) | Total A  (110) | 501.9 ^a^ | 446.8  (70.5)^b^ | 42.6  (11.5)^b^ | 9.6  (3.8)^b^ | 2.7  (1.9)^b^ | -- | 55.0  (15.6)^b^ |
|  |  |  |  |  | Total B  (109) | 488.1^a^ | 440.3  (70.9)^b^ | 37.7  (13.4)^b^ | 6.9  (3.6)^b^ | 3.0  (2.0)^b^ | -- | 47.7  (17.9)^b^ |
|  |  |  |  | Van Cauwenberghe (2011) | Total C  (151) | 158.2^a^ | 139.3  (38.3) | 8.9  (4.5) | 5.8  (3.6) | 4.1 (3.4) | -- | 18.9  (11.2) |
| Bonis  (2014) | GT3X+  (Right hip) | 2 weekdays;  during childcare hours | 2 days;  ≥ 9 clean hours of 2 days;  ≥ 10 min;  15-s | Van Cauwenberghe (2011) | Total  (209) | 540.0 | 485.6  (31.7) | 27.7  (13.1) | 18.0  (12.6) | 8.7  (8.5) | 26.7  (10.6) | 54.4  (30.2) |
| First Author (Year) | Measure | | | | Sample  (n) | Average valid weartime during setting (min) | ST | LPA | MPA | VPA | MVPA | TPA |
|  | Device;  (Wear location) | Number wear Days;  Wear duration | Valid days;  Valid hours;  Non-wear time;  Epoch length | Cutpoint |  |  | Mean (SD) | Mean (SD) | Mean (SD) | Mean (SD) | Mean (SD) | Mean (SD) |
| Byun  (2013) | GT1M  (Right hip) | 5 weekdays;  waking hours | ≥ 3 days;  ≥ 50% of school hours;  NR;  15-s | Pate  (2006) | Total A  (164) | 348.0 | 257.5 (66.5)^b^ | -- | -- | -- | -- | -- |
|  |  |  |  |  | Boys A  (81) |  | 252.3  (46.8) ^b^ | -- | -- | -- | -- | -- |
|  |  |  |  |  | Girls A  (83) |  | 263.3  (52.8) ^b^ | -- | -- | -- | -- | -- |
|  |  |  |  |  | Total B  (167) | 354.0 | 277.8  (68.4) ^b^ | -- | -- | -- | -- | -- |
|  |  |  |  |  | Boys B  (88) |  | 271.4  (49.7) ^b^ | -- | -- | -- | -- | -- |
|  |  |  |  |  | Girls B  (79) |  | 282.0  (47.1) ^b^ | -- | -- | -- | -- | -- |
| Byun  (2015) | 7164  (Right hip) | 8 to 10 days;  NR | ≥ 4 weekdays;  ≥ 5 hours in school;  ≥ 60 min;  15-s | Sirard  (2005) | Total  (191) | 504.0 | 269.6  (33.2) | -- | -- | -- | -- | -- |
|  |  |  |  |  | Total  (191) |  | 373.8  (29.3) | -- | -- | -- | -- | -- |
|  |  |  |  | Van Cauwenberghe (2011) | Total  (191) |  | 430.9 (16.1) | -- | -- | -- | -- | -- |
| Carson  (2015) | Actical  (Right hip) | 5 weekdays;  during childcare hours | ≥ 3 days;  ≥ 1 hour;  > 20 min and nap time;  15-s | Colley (2013) | Total A  (36) | 336.0 | 211.6  (23.5)^b^ | 101.3  (19.8)^b^ | -- | -- | 22.4  (13.4)^b^ | -- |
|  |  |  |  |  | Total B  (50) |  | 197.1  (43.1)^b^ | 109.2  (23.3)^b^ | -- | -- | 29.6  (27.7)^b^ | -- |
| First Author (Year) | Measure | | | | Sample  (n) | Average valid weartime during setting (min) | ST | LPA | MPA | VPA | MVPA | TPA |
|  | Device;  (Wear location) | Number wear Days;  Wear duration | Valid days;  Valid hours;  Non-wear time;  Epoch length | Cutpoint |  |  | Mean (SD) | Mean (SD) | Mean (SD) | Mean (SD) | Mean (SD) | Mean (SD) |
| Carson  (2016) | GT1M  (Right hip) | 7 days;  waking hours | ≥ 3 days;  ≥ 6 hours and  ≥ 50% during childcare;  ≥ 10 min;  15-s | Janssen (2013) | Total  (177) | 263.8 | 126.1  (99.7)^b^ | -- | -- | -- | -- | -- |
| Copeland  (2016) | Actical  (Right hip) | 1 day;  24h | 1 day;  NR;  ≥ 30 min;  15-s | Pfeiffer  (2006) | Total  (388) | 360.0 | 216.6  (35.4)^b^ | 128.4  (35.4)^b^ | -- | -- | 14.4  (7.8)^b^ | -- |
| Dawson-Hahn  (2015) | GT1M  (Hip) | NR;  NR | ≥ 5 days;  ≥ 3 hours;  ≥ 60 min, aside from 1 to 2 min of counts between 0–100;  15-s | Pate  (2006) | Total  (96) | NR | -- | -- | -- | -- | -- | 98.4  (18.1) |
| Delaney  (2014) | GT1M  (NR) | 5 weekdays;  during childcare hours | 5 days;  ≥ 3 hours;  ≥ 30 min;  10-s | Pfeiffer  (2006) | Total  (144) | 292.8 ^a^ | 167.3  (22.4) | 82.4  (12.2) | 42.9  (12.6) | 15.1  (6.8) | -- | -- |
| Ellis  (2017) | ActiPAL  (Upper thigh) | 5 weekdays;  during childcare hours | > 1 day;  ≥ 3 hours (nap time was excluded for toddlers);  NR;  NR | NR | Total  (301) | 309.0 | 249.3  (64.1)^b^ | -- | -- | -- | -- | 58.9  (20.8)^b^ |
|  |  |  |  |  | Boys  (145) | 307.0 | 244.4  (81.8)^b^ | -- | -- | -- | -- | 64.1  (18.0)^b^ |
|  |  |  |  |  | Girls  (156) | 310.0 | 253.9 (96.1)^b^ | -- | -- | -- | -- | 54.6  (14.9)^b^ |
| First Author (Year) | Measure | | | | Sample  (n) | Average valid weartime during setting (min) | ST | LPA | MPA | VPA | MVPA | TPA |
|  | Device;  (Wear location) | Number wear Days;  Wear duration | Valid days;  Valid hours;  Non-wear time;  Epoch length | Cutpoint |  |  | Mean (SD) | Mean (SD) | Mean (SD) | Mean (SD) | Mean (SD) | Mean (SD) |
| Erinosho  (2016) | GT1M  (Waist) | 4 weekdays;  during childcare hours except nap time | 4 days;  ≥ 4 hours;  NR;  5-s converted to 15-s | Evenson (2008)  Pate  (2006) | Total  (544) | 408.0 | 205.9  (48.3) | -- | -- | -- | 38.0  (19.0) | -- |
| Gagné  (2013) | Actigraph  (NR) | 4 weekdays;  during childcare hours | 4 days;  ≥ 4 hours;  ≥ 40 min;  15-s | Sirard (2005) | Total  (242) | 480.0 | -- | -- | -- | -- | 12.6  (9.2) | 53.0  (23.6) |
| Goldfield  (2016) | Actical  (NR) | 5 weekdays;  during childcare hours | ≥ 2 days;  ≥ 4 hours;  NR;  15-s | Adolph  (2012)  Pfeiffer  (2006) | Total A  (43) | 527.9 | 237.7  (32.1)^b^ | 127.3  (25.5)^b^ | -- | -- | 35.3  (11.1) ^b^ | 162.9  (32.1)^b^ |
|  |  |  |  |  | Total B  (40) | 519.5 | 251.8  (33.5) ^b^ | 118.9  (26.5)^b^ | -- | -- | 29.9  (13.9) ^b^ | 148.8  (33.5)^b^ |
| Henderson  (2015) | GT1M  (Right hip) | NR;  during childcare | NR;  NR;  ≥ 60 min;  5-s | Evenson  (2008) | Total  (389) | 198.0 | 98.4  (18.2) | 72.4  (12.0) | 15.6  (5.1) | 11.6  (6.1) | 27.3  (11.2) | -- |
| Hesketh  (2014) | Actiheart  (Left chest) | 7 days;  24h | ≥ 1 weekday;  ≥ 10 hours;  ≥ 100 min;  60-s | Light (>20 CPM) Moderate  (>400 CPM), and Vigorous (>600 CPM) | Total A  (593) | NR | 91.1  (40.0) | 185.4  (47.5) | 11.7  (9.4) | 10.7  (11.6) | 22.6  (19.0) | -- |
|  |  |  |  |  | Total B  (593) |  | 77.8  (46.6) | 189.9  (39.2) | 16.0  (11.2) | 15.6  (15.2) | 32.0  (23.9) | -- |
| First Author (Year) | Measure | | | | Sample  (n) | Average valid weartime during setting (min) | ST | LPA | MPA | VPA | MVPA | TPA |
|  | Device;  (Wear location) | Number wear Days;  Wear duration | Valid days;  Valid hours;  Non-wear time;  Epoch length | Cutpoint |  |  | Mean (SD) | Mean (SD) | Mean (SD) | Mean (SD) | Mean (SD) | Mean (SD) |
| Hinkley  (2016) | GT1M  (Right hip) | 8 days;  waking hours | ≥ 2 weekdays;  ≥ 50% during childcare;  ≥ 20 min  15-s | >100 CPM | Boys  (395) | NR | -- | -- | -- | -- | -- | 183.9  (NR) |
|  |  |  |  |  | Girls  (336) |  | -- | -- | -- | -- | -- | 170.4  (NR) |
| Kuzik  (2016) | Actical  (Right hip) | 5 weekdays;  during childcare hours | ≥ 3 days;  ≥ 1 hour;  ≥ 20 min and nap time;  15-s | Colley  (2013) | Total  (100) | 342.0 | 204.0  (30.0) | 108.0  (18.0) | -- | -- | 30.0  (18.0) | 138.0  (24.0) |
| LaRowe  (2016) | Actical  (Hip) | NR;  during childcare hours | NR;  NR;  NR;  15-s | Puyau  (2004) | Total  (231) | 420.9 | 254.6  (13.3) | 156.6 (12.4) | -- | -- | 9.6  (2.4) | -- |
| Loprinzi  (2010) | 7164  (Right hip) | NR;  during childcare hours | ≥ 2 childcare days;  ≥ 4 hours;  ≥ 10 min;  15-s | Sirard  (2005) | Total  (156) | 330.0 | -- | -- | -- | -- | 50.0  (19.2) | -- |
|  |  |  |  |  | Boys  (75) |  | -- | -- | -- | -- | 51.1  (21.4) | -- |
|  |  |  |  |  | Girls  (81) |  | -- | -- | -- | -- | 49.5  (17.6) | -- |
| Mazzucca  (2018) | GT1M  (NR) | 4 weekdays;  during childcare hours | NR;  NR;  ≥ 60 min;  15-s | Pate  (2006) | Total  (559) | 509.4 | 294.6  (35.6) | 146.0  (19.5) | -- | -- | 68.7  (19.5) | -- |
| First Author (Year) | Measure | | | | Sample  (n) | Average valid weartime during setting (min) | ST | LPA | MPA | VPA | MVPA | TPA |
|  | Device;  (Wear location) | Number wear Days;  Wear duration | Valid days;  Valid hours;  Non-wear time;  Epoch length | Cutpoint |  |  | Mean (SD) | Mean (SD) | Mean (SD) | Mean (SD) | Mean (SD) | Mean (SD) |
| Møller  (2017) | GT3X  (Right hip) | 7 days;  24h | ≥ 4 weekdays;  ≥ 8 hours;  ≥ 20 min;  10-s | Pate  (2006) | Boys  (114) | 384.0 | 183.9  (30.3) | -- | -- | -- | 49.9  (16.1) | 200.0  (30.3) |
|  |  |  |  |  | Girls  (117) |  | 196.6  (35.7) | -- | -- | -- | 41.4  (16.5) | 187.7  (35.7) |
| Neshteruk  (2018) | GT3X+  (Right hip) | 3 weekdays;  waking hours | ≥ 2 days;  ≥ 2.5 hours during childcare;  ≥ 60 min  15-s | Evenson (2008)  Pate  (2006) | Total  (496) | 390.0 | 250.2  (27.9) | 109.2  (20.8) | -- | -- | 30.5  (10.4) | -- |
| Nielsen  (2012) | 7164  (Lower back) | 4 days (2 x 2);  waking hours | ≥ 3 days;  ≥ 8 hours;  ≥ 10 min  10-s | MPA (2,500 to  5,000 CPM) VPA > 5,000 CPM | Total  (594) | NR | -- | -- | 17.7  (9.2) | 5.8  (4.8) | -- | -- |
|  |  |  |  |  | Boys  (310) |  | -- | -- | 19.5  (9.9) | 6.3 (5.2) | -- | -- |
|  |  |  |  |  | Girls  (284) |  | -- | -- | 15.7  (7.9) | 5.2  (4.3) | -- | -- |
| Olesen  (2013) | GT1M/ GT3X  (Right hip) | 7 days;  during childcare hours | ≥ 3 weekdays;  ≥ 3 hours during childcare;  ≥ 60 min;  15-s | Evenson (2008) | Boys  (211) | 429.0 | -- | -- | -- | -- | 64.3  (21.0) | -- |
|  |  |  |  |  | Girls  (215) |  | -- | -- | -- | -- | 52.3 (16.7) | -- |
| O’Dwyer  (2014) | GT1M  (Right hip) | 7 days;  waking hours | ≥ 3 day (2x1);  ≥ 80% during childcare hours;  ≥ 20 min;  5-s | Sirard  (2005) | Boys  (100) | NR | -- | -- | -- | -- | 26.5  (12.4) | -- |
|  |  |  |  |  | Girls  (88) |  | -- | -- | -- | -- | 22.2  (9.2) | -- |
| First Author (Year) | Measure | | | | Sample  (n) | Average valid weartime during setting (min) | ST | LPA | MPA | VPA | MVPA | TPA |
|  | Device;  (Wear location) | Number wear Days;  Wear duration | Valid days;  Valid hours;  Non-wear time;  Epoch length | Cutpoint |  |  | Mean (SD) | Mean (SD) | Mean (SD) | Mean (SD) | Mean (SD) | Mean (SD) |
| O’Neill  (2016) | GT1M / GT3X  (Right hip) | 5 weekdays;  waking hours | ≥ 2 days;  NR;  NR;  15-s | Pate  (2006)  Espanã-Romero (2013) | Total  (341) | 330.0 | -- | -- | -- | -- | 39.0  (14.8) | 77.5  (24.7) |
|  |  |  |  |  | Boys  (174) |  | -- | -- | -- | -- | 41.8 (15.4) | 81.9  (24.7) |
|  |  |  |  |  | Girls  (167) |  | -- | -- | -- | -- | 35.7  (13.7) | 73.1 (24.2) |
| Pagels  (2010) | GT1M  (NR) | 5 weekdays;  during childcare hours | NR;  NR;  NR;  15-s | Sirard  (2005) | Boys  (28) | 433.2 | 396.0  (55.0) | 50.0  (19.0) | -- | -- | 16.6  (9.5) | -- |
|  |  |  |  |  | Girls  (27) |  | 397  (71.0) | 46.0  (15.0) | -- | -- | 17.4  (10.0) | -- |
| Pate  (2004) | 7164  (Right hip) | 1 to 11 days;  NR | ≥ 3 days;  ≥ 1 hour;  NR;  15-s | Sirard  (2001) | Total  (247) | 276.0 | 193.6  (26.6) | 48.3  (14.7) | -- | 8.7  (5.0) | 35.4  (14.2) | -- |
|  |  |  |  |  | Boys  (115) | 270.0 | 187.6  (24.7) | 46.8  (13.0) | -- | 9.4  (4.9) | 35.1  (13.9) | -- |
|  |  |  |  |  | Girls  (132) | 282.0 | 199.7  (28.6) | 49.8  (16.4) | -- | 7.99  (5.1) | 32.4  (14.1) | -- |
| First Author (Year) | Measure | | | | Sample  (n) | Average valid weartime during setting (min) | ST | LPA | MPA | VPA | MVPA | TPA |
|  | Device;  (Wear location) | Number wear Days;  Wear duration | Valid days;  Valid hours;  Non-wear time;  Epoch length | Cutpoint |  |  | Mean (SD) | Mean (SD) | Mean (SD) | Mean (SD) | Mean (SD) | Mean (SD) |
| Pate  (2014) | GT1M / GT3X  (Right hip) | 5 weekdays;  waking time | ≥ 1 weekday;  ≥ 50% in childcare hours;  ≥ 60 min;  15-s | Pate  (2006)  Espanã-Romero (2013) | Total A  (145) | 342.0 | -- | 43.8  (26.4)^b^ | -- | -- | 43.8  (33.7) ^b^ | 87.7  (61.4)^b^ |
|  |  |  |  |  | Boys A (72) |  | -- | 45.0  (18.4)^b^ | -- | -- | 46.1  (28.5) ^b^ | 91.2  (42.8)^b^ |
|  |  |  |  |  | Girls A  (73) |  | -- | 40.1  (19.5)^b^ | -- | -- | 41.8  (24.6) ^b^ | 81.4  (45.0)^b^ |
|  |  |  |  |  | Total B  (156) | 354.0 | -- | 38.3  (28.7)^b^ | -- | -- | 38.3  (36.2) ^b^ | 76.7  (66.1)^b^ |
|  |  |  |  |  | Boys B  (77) |  | -- | 43.3 (19.1)^b^ | -- | -- | 41.0  (29.5) ^b^ | 83.7  (44.3)^b^ |
|  |  |  |  |  | Girls B  (79) |  | -- | 37.1  (20.2)^b^ | -- | -- | 34.8  (25.5) ^b^ | 71.3  (46.6)^b^ |
| Pate  (2016) | GT1M / GT3X  (Right hip) | 5 weekdays;  waking time | ≥ 3 days;  ≥ 50% of the school day  ≥ 60 min;  15-s | Pate  (2006) | Total A  (191) | 330.0 | 252.4  (27.5) | 39.6  (8.8) | -- | -- | 37.9  (15.4) | 77.5  (27.5) |
|  |  |  |  |  | Boys A  99) | 336.0 | 252.5  (27.4) | 42.0  (14.0) | -- | -- | 41.4  (15.6) | 83.4  (26.8) |
|  |  |  |  |  | Girls A  92) | 324.0 | 252.1  (27.5) | 37.2  (14.5) | -- | -- | 34.0  (14.5) | 71.8  (27.0) |
|  |  |  |  |  | Total B  (188) | 306.0 | 236.1  (22.4) | 34.6  (9.6) | -- | -- | 35.7  (13.7) | 69.8  (22.4) |
|  |  |  |  |  | Boys B  (92) | 294.0 | 219.5  (20.5) | 35.2  (8.8) | -- | -- | 38.7  (12.7) | 74.4  (20.5) |
|  |  |  |  |  | Girls B  96) | 318.0 | 252.2  (21.7) | 33.3  (9.5) | -- | -- | 31.8  (13.2) | 65.7  (21.7) |
| First Author (Year) | Measure | | | |  | Average valid weartime during setting (min) | ST | LPA | MPA | VPA | MVPA | TPA |
|  | Device;  (Wear location) | Number wear Days;  Wear duration | Valid days;  Valid hours;  Non-wear time;  Epoch length | Cut-point | Sample  (n) |  | Mean (SD) | Mean (SD) | Mean (SD) | Mean (SD) | Mean (SD) | Mean (SD) |
| Razak  (2018) | GT3X+  (Left hip) | 5 weekdays;  during childcare hours | ≥ 1 day;  ≥ 50% childcare hours;  NR;  5-s | Pate  (2006) | Total A  (217) | NR | -- | 53.2  (11.5) | 31.9  (9.6) | 19.8  (8.5) | 51.7  (17.3) | 104.9 (26.7) |
|  |  |  |  |  | Total B  (161) |  | -- | 54.9  (14.1) | 34.9  (11.3) | 23.5  (11.0) | 58.5  (21.1) | 113.4 (32.1) |
| Rice  (2014) | GT1M  (Right hip) | 5 weekdays;  during childcare hours | ≥ 2 days;  ≥ 75% during childcare hours;  ≥ 60 min;  NR | Van Cauwenberghe  (2011) | Total  (114) | 330.0 | -- | -- | -- | -- | 31.9  (17.6) | 57.2  (24.2) |
| Schlechter  (2017) | GT1M  (Right hip) | NR;  during childcare hours | NR;  NR;  NR;  15-s | Van Cauwenberghe  (2011) | Total  (73) | 385.0 | 267.5  (47.6) | -- | -- | -- | -- | 117.4  (51.9) |
| Schuna  (2016) | ActiTrainer  (Right hip) | 5 weekdays;  during childcare hours | ≥ 2 days;  ≥ 3 hours;  ≥ 10 min;  5-s | Pate  (2006) | Total A  (62) | 319.8 | 178.3  (27.5)^b^ | 90.9  (18.1)^b^ | 31.7  (1.5)^b^ | -- | 50.5  (17.3)^b^ | -- |
|  |  |  |  |  | Total A  (62) | 305.7 | 186.3  (28.3)^b^ | 40.1  (19.6)^b^ | 24.3  (8.6)^b^ | -- | 39.3  (17.3)^b^ | -- |
| Shen  (2012) | RT3  (NR) | 5 weekdays;  during childcare hours | ≥ 2 days;  ≥ 1 hour (excluding eating time);  NR;  60-s | Trost  (2003)  Pate  (2004) | Total  (158) | 138.0 | 128.8  (10.3) | 7.1 (3.1) | 2.0  (0.2) | 0  (0) | 6.3  (2.0) | -- |
|  |  |  |  |  | Boys  (80) |  | 128.5  (11.2) | 7.5  (3.2) | 2.2  (0.3) | 0  (0) | 10.5  (3.2) | -- |
|  |  |  |  |  | Girls  (78) |  | 129.4  (9.7) | 6.6  (2.7) | 1.8  (0.2) | 0  (0) | 8.1  (2.7) | -- |
| First Author (Year) | Measure | | | | Sample  (n) | Average valid weartime during setting (min) | ST | LPA | MPA | VPA | MVPA | TPA |
|  | Device;  (Wear location) | Number wear Days;  Wear duration | Valid days;  Valid hours;  Non-wear time;  Epoch length | Cut-point |  |  | Mean (SD) | Mean (SD) | Mean (SD) | Mean (SD) | Mean (SD) | Mean (SD) |
| Shen  (2013) | GT1M  (Right hip) | 6 days;  waking hours | NR;  NR;  NR;  15-s | Sirard (2005) | Boys A  (20) | 408.0 | -- | 72.1  (29.2) | 19.1  (10.5) | 7.1  (7.5) | -- | 98.3  (32.0) |
|  |  |  |  |  | Boys B  (20) |  | -- | 72.2 (27.8) | 19.1  (11.1) | 6.6  (5.6) | -- | 97.9  (33.2) |
|  |  |  |  |  | Girls A  (26) |  | -- | 66.1  (37.0) | 18.2  (10.7) | 4.3  (4.3) | -- | 88.2  (39.6) |
|  |  |  |  |  | Girls B  (26) |  | -- | 67.6  (26.3) | 18.2  (10.7) | 5.0  (5.9) | -- | 90.90 (33.1) |
| Sisson  (2017) | GT3X  (Waist) | 2 weekdays;  during childcare hours | ≥ 1 day;  NR;  ≥ 30 min;  5-s | Sirard  (2005) | Total  (82) | 354.0 | 297.5  (92.0) | 33.0  (14.0) | 16.2  (10.2) | 8.8  (7.1) | 25.3 (12.9) | -- |
| Soini  (2014) | GT3X  (NR) | 5 days (3x2);  during childcare hours | ≥ 1 weekday  ≥ 7.5 hours;  ≥ 10 min;  5-s | Pate  (2006) | Total A  (80) | 462.0 | -- | -- | -- | -- | 48.0  (24.0) | 147.0  (55.0) |
|  |  |  |  |  | Total B  (41) | 486.0 | -- | -- | -- | -- | 53.0  (34) | 163.0  (79.0) |
| Sugiyama  (2012) | GT1M  (Waist right) | 3 weekdays;  during childcare hours | NR;  NR;  NR;  15-s | Sirard (2005) | Total  (89) | 397.5 | 323.1 (68.5) | -- | -- | -- | 24.3  (12.6) | -- |
| Tandon  (2015) | GT3X+  (Right hip) | 4 weekdays;  during childcare hours | NR;  NR;  NR;  15-s | Pate  (2006) | Total  (98) | 384.2 | 279.3  (NR) | 49.7  (NR) | -- | -- | 55.2  (NR) | -- |
| First Author (Year) | Measure | | | | Sample  (n) | Average valid weartime during setting (min) | ST | LPA | MPA | VPA | MVPA | TPA |
|  | Device;  (Wear location) | Number wear Days;  Wear duration | Valid days;  Valid hours;  Non-wear time;  Epoch length | Cut-point |  |  | Mean (SD) | Mean (SD) | Mean (SD) | Mean (SD) | Mean (SD) | Mean (SD) |
| Tucker  (2015) | Actical  (Right hip) | 5 weekdays;  during childcare hours | 3 days;  ≥ 5 hours;  ≥ 60 min;  15-s | Pfeiffer  (2006) | Total A  (71) | 406.2 | 278.8  (25.3)^b^ | -- | -- | -- | -- | -- |
|  |  |  |  |  | Total B  (20) |  | 272.8  (42.4)^b^ | -- | -- | -- | -- | -- |
|  |  |  |  |  | Total C  (127) |  | 265.8  (22.9)^b^ | -- | -- | -- | -- | -- |
| Tucker  (2017) | Actical  (Right hip) | 5 weekdays;  during childcare hours | 2 days;  ≥ 5 hours;  ≥ 20 min;  15-s | Adolph  (2012) | Total A  (138) | 429.7 ^a^ | 235.2  (37.6) | 151.8  (26.4) | -- | -- | 42.7  (16.6) | 194.4  (36.7) |
|  |  |  |  |  | Total B  (200) | 434.2 ^a^ | 238.6  (30.0) | 156.3  (21.2) | -- | -- | 38.9  (15.3) | 195.5  (30.0) |
| Vale  (2009) | 7164  (NR) | 5 weekdays;  during childcare hours | 4 days;  ≥ 6 hours;  NR;  5-s and 60-s | Sirard  (2005) | Total A  (59) | NR | -- | -- | 18.4  (7.0) | 8.0  (3.8) | 26.4  (9.6) | -- |
|  |  |  |  |  | Total B  (59) |  | -- | -- | 9.5  (8.0) | 0.5  (0.7) | 10.0  (8.4) | -- |
| Van Cauwenberghe  (2012a) | GT1M  (NR) | 5 weekdays;  entire day | NR;  ≥ 8 hours;  NR;  15-s | Van Cauwenberghe  (2011) | Total  (107) | 463.7 | 390.2  (22.7)^b^ | -- | -- | -- | 40.6  (17.5)^b^ | 73.5  (22.7)^b^ |
|  |  |  |  |  | Boys  (60) |  | 380.8  (22.4)^b^ | -- | -- | -- | 47.7  (17.0)^b^ | 82.8  (22.4)^b^ |
|  |  |  |  |  | Girls  (47) |  | 401.0  (22.6)^b^ | -- | -- | -- | 32.4  (17.1)^b^ | 62.7  (22.6)^b^ |
| Van Cauwenberghe  (2012b) | ActiCal  (Hip) | NR;  during childcare hours | NR;  ≥ 3 hours;  NR;  15-s | Evenson (2008) | Total A (49) | 294.8 | 150.5  (52.8) | -- | -- | -- | -- | -- |
|  | ActiPAL  (Thigh) |  |  | PALTech (2008) | Total A  (49) |  | 149.2  (48.9) | -- | -- | -- | -- | -- |
| First Author (Year) | Measure | | | | Sample  (n) | Average valid weartime during setting (min) | ST | LPA | MPA | VPA | MVPA | TPA |
|  | Device;  (Wear location) | Number wear Days;  Wear duration | Valid days;  Valid hours;  Non-wear time;  Epoch length | Cutpoint |  |  | Mean (SD) | Mean (SD) | Mean (SD) | Mean (SD) | Mean (SD) | Mean (SD) |
| Van Cauwenberghe  (2013) | GT1M  (Right hip) | 4 weekdays;  waking hours | NR;  ≥ 8 hours;  ≥ 10 min;  15-s | Van Cauwenberghe  (2011) | Total  (200) | 474.0 | 394.0  (28.2)^b^ | 36.1  (12.7)^b^ | -- | -- | 44.2  (18.3)^b^ | 80.3  (28.2)^b^ |
|  |  |  |  |  | Total  (200) |  | 407.0  (28.2)^b^ | 33.2  (12.7)^b^ | -- | -- | 34.1  (18.3)^b^ | 67.3  (28.2)^b^ |
| Vanderloo  (2014) | Actical  (Right hip) | 1 weekday;  during childcare hours | 1 day;  ≥ 4 hours;  NR;  15-s | Pfeiffer  (2006) | Total  (31) | 451.7 | 305.3  (68.5) | -- | -- | -- | 11.4  (10.6) | 132.6  (46.5) |
| Vanderloo  (2015a) | Actical  (Right hip) | 5 weekdays;  during childcare hours | ≥ 1 day;  ≥ 5 hours;  ≥ 60 min;  15-s | Pfeiffer  (2006) | Monday  (99) | 346.0 | 256.2  (35.4) | 83.2  (27.5) | -- | -- | 8.5  (18.0) | 89.9  (31.6) |
|  |  |  |  |  | Tuesday  (101) | 379.9 | 259.7  (52.6) | 112.4  (51.7) | -- | -- | 8.8  (8.9) | 120.2  (52.2) |
|  |  |  |  |  | Wednesday  (100) | 371.5 | 264.7  (46.3) | 97.4  (42.3) | -- | -- | 9.5  (8.4) | 107.0  (45.5) |
|  |  |  |  |  | Thursday  (101) | 336.8 | 228.0  (63.0) | 102.33  (60.6) | -- | -- | 7.1  (5.9) | 109.5  (60.5) |
|  |  |  |  |  | Friday  (101) | 333.9 | 238.9  (40.4) | 88.4  (36.3) | -- | -- | 6.5  (5.7) | 95.0  (38.9) |
| Vanderloo  (2015b) | Actical  (Right hip) | 5 weekdays;  during childcare hours | ≥ 3 days;  ≥ 5 hours;  ≥ 60 min;  15-s | Pfeiffer  (2006) | Total A  (71) | 406.2 | -- | -- | -- | -- | 10.5  (4.9) | 123.0  (22.7) |
|  |  |  |  |  | Total B  (20) |  | -- | -- | -- | -- | 11.7  (6.4) | 129.1  (42.4) |
|  |  |  |  |  | Total C  (127) |  | -- | -- | -- | -- | 22.3  (8.7) | 136.0  (25.8) |
| First Author (Year) | Measure | | | | Sample  (n) | Average valid weartime during setting (min) | ST | LPA | MPA | VPA | MVPA | TPA |
|  | Device;  (Wear location) | Number wear Days;  Wear duration | Valid days;  Valid hours;  Non-wear time;  Epoch length | Cutpoint |  |  | Mean (SD) | Mean (SD) | Mean (SD) | Mean (SD) | Mean (SD) | Mean (SD) |
| Vanderloo  (2016) | Actical  (Right hip) | 5 weekdays;  during childcare hours | ≥ 3 days;  ≥ 5 hours;  ≥ 60 min;  NR | Pfeiffer  (2006) | Total A  (113) | 379.0 | 251.9  (21.0) | 106.5  (20.1) | -- | -- | 21.8  (7.5) | 128.3  (21.7) |
|  |  |  |  |  | Total B  (113) |  | 251.0  (20.5) | 108.1  (17.9) | -- | -- | 21.4  (8.1) | 129.6  (21.3) |
|  |  |  |  |  | Boys A  (52) |  | 247.2  (22.5) | 108.6  (21.3) | -- | -- | 24.3  (6.5) | 132.9  (22.9) |
|  |  |  |  |  | Boys B  (52) |  | 251.5  (21.2) | 109.9  (18.7) | -- | -- | 19.8  (8.0) | 129.7  (22.2) |
|  |  |  |  |  | Girls A  (61) |  | 255.3  (19.6) | 105.0  (19.6) | -- | -- | 19.9  (7.7) | 124.9  (20.6) |
|  |  |  |  |  | Girls B  (61) |  | 250.7  (19.5) | 106.6  (17.2) | -- | -- | 22.9  (8.0) | 129.6  (20.8) |
| Ward  (2017) | Actical  (Hip) | 5 weekdays;  during childcare hours | 4 days;  ≥ 2 hours;  NR;  15-s | Pfeiffer (2006)  Wong (2011) | Total  (624) | 478.6 | 306.7  (59.4) | 162.2  (53.6) | -- | -- | 9.7  (9.3) | 171.9  (55.6) |
| Webster  (2015) | Actical  (Right hip) | 4 weekdays;  during childcare hours | 4 weekdays;  NR;  NR;  15-s | Pfeiffer  (2006) | Total  (118) | 257.6 | -- | -- | 5.3  (NR) | 2.2  (NR) | 7.5  (NR) | -- |
|  |  |  |  |  | Total  (118) |  | -- | -- | 5.8  (NR) | 3.1  (NR) | 9.0  (NR) | -- |
| **School** | | | | | | | | | | | | |
| Aadland  (2018) | GT3X+  (NR) | 7 days;  waking hours | 5 days;  ≥ 8 hours and ≥4 hours during school hours;  ≥ 20 min;  10-s | Evenson (2­008) | Total A  (465) | 295.0 | 183.0  (18.0) | 85.0  (13.0) | 16.0  (5.0) | 13.0  (6.0) | 26.0  (10.0) | -- |
| First Author (Year) | Measure | | | | Sample  (n) | Average valid weartime during setting (min) | ST | LPA | MPA | VPA | MVPA | TPA |
|  | Device;  (Wear location) | Number wear Days;  Wear duration | Valid days;  Valid hours;  Non-wear time;  Epoch length | Cutpoint |  |  | Mean (SD) | Mean (SD) | Mean (SD) | Mean (SD) | Mean (SD) | Mean (SD) |
| Abbott  (2013) | Actical  (Hip) | 7 days;  waking hours | 4 days (3x1);  ≥10 hours;  NR;  NR | Puyau (2002) | Total  (53) | 375.0 | 250.5  (27.3) | -- | -- | -- | -- | -- |
| Aibar  (2014) | GT3X+  (Right hip) | 8 days;  waking hours | ≥ 3 weekdays;  ≥ 80% during school hours;  ≥ 10 min, with allowance for 1-2 minutes of counts between 0-100  15-s | Evenson (2008) | Total A  (352) | 336.9 | -- | -- | -- | -- | 23.0  (0.5) | -- |
|  |  |  |  |  | Total B  (359) | 390.6 | -- | -- | -- | -- | 28.5  (0.5) | -- |
| Andersen  (2015) | GT3X  (Hip) | 7 days;  waking hours | ≥ 1 weekday;  NR;  ≥ 60 min;  2-s | Evenson (2008) | Boys  (148) | 310.6 | 178.5  (NR) | -- | -- | -- | 27.1  (NR) | -- |
|  |  |  |  |  | Girls  (168) | 321.0 | 205.3  (NR) | -- | -- | -- | 19.4  (NR) | -- |
|  |  |  |  |  | Total  (316) | 316.1 | 192.7  (40.9) | -- | -- | -- | 23.0  (11.9) | -- |
| Baere  (2016) | SWM  (Left arm) | 7 days;  24 hours | NR;  NR;  NR;  60-s | De Baere  (2015) | Boys  (99) | 262.2 | 106.5  (6.3) | 131.6  (10.5) | 17.0  (2.0) | 7.1  (3.4) | 24.1  (4.5) | -- |
|  |  |  |  |  | Girls  (102) | 260.8 | 123.7  (6.1) | 121.1  (7.2) | 10.8  (2.4) | 4.2  (2.8) | 15.0  (5.3) | -- |
| Baily  (2012) | RT3  (NR) | 7 days;  NR | ≥ 3 days;  ≥ 9 hours;  ≥ 10 min;  60-s | Rowland  (2004) | Boys  (57) | 390.5 | 241.7  (38.5) | 79.6  (24.4) | 48.1  (17.1) | 21.1  (17.4) | 69.3  (29.1) | -- |
|  |  |  |  |  | Girls  (78) | 405.4 | 267.4  (41.3) | 83.9  (23.8) | 44.2  (16.8) | 9.9  (9.0) | 54.1  (22.4) | -- |
| First Author (Year) | Measure | | | | Sample  (n) | Average valid weartime during setting (min) | ST | LPA | MPA | VPA | MVPA | TPA |
|  | Device;  (Wear location) | Number wear Days;  Wear duration | Valid days;  Valid hours;  Non-wear time;  Epoch length | Cutpoint |  |  | Mean (SD) | Mean (SD) | Mean (SD) | Mean (SD) | Mean (SD) | Mean (SD) |
| Bershwinger  (2013) | NL-1000  (NR) | 5 weekdays;  NR | NR;  NR;  NR;  NR | NR | Total A  (18) | NR | -- | -- | -- | -- | 17.7  (2.8) | -- |
|  |  |  |  |  | Total B  (18) |  | -- | -- | -- | -- | 24.2  (3.4) | -- |
| Brittin  (2017) | GT3X+ / GT1M  (right hip) | 5 to 7 days during 3 weekdays;  during school hours | ≥ 3 days;  ≥ 7 hours or ≥ 5 or 6 hours during school hours;  ≥ 30 min;  60-s | Evenson  (2008) | Total A  (32) | 420.0 | 265.2  (39.7) | 129.8  (34.2) | -- | -- | 25.0  (9.6) | -- |
|  |  |  |  |  | Total B  (21) | 393.3 | 214.9  (37.6) | 167.2  (35.3) | -- | -- | 11.2  (4.9) | -- |
| Brusseau  (2016) | wGT3X-BT  (Right hip) | 5 weekdays;  during school hours | ≥ 3 days;  ≥ 6 hours;  NR;  15-s | Evenson  (2008) | Total  (395) | NR | -- | -- | -- | -- | 22.5  (12.6) | -- |
|  |  |  |  |  | Boys  (217) |  | -- | -- | -- | -- | 24.2  (13.3) | -- |
|  |  |  |  |  | Girls  (178) |  | -- | -- | -- | -- | 20.9  (11.5) | -- |
| Burns  (2016) | WGT3X-BT  (Hip) | 5 weekdays;  during school hours | ≥ 4 days;  NR;  NR;  5-s | Evenson  (2008) | Boys (524) | NR | -- | -- | -- | -- | 27.5  (13.7) | 62.5  (15.5) |
|  |  |  |  |  | Girls  (525) |  | -- | -- | -- | -- | 22.2  (11.5) | 58.7  (14.2) |
|  |  |  |  |  | Total  (1,049) |  | -- | -- | -- | -- | 24.8  (12.9) | 61.1  (14.9) |
| First Author (Year) | Measure | | | | Sample  (n) | Average valid weartime during setting (min) | ST | LPA | MPA | VPA | MVPA | TPA |
|  | Device;  (Wear location) | Number wear Days;  Wear duration | Valid days;  Valid hours;  Non-wear time;  Epoch length | Cutpoint |  |  | Mean (SD) | Mean (SD) | Mean (SD) | Mean (SD) | Mean (SD) | Mean (SD) |
| Burns  (2018) | WGT3X-BT  (Right hip) | 5 weekdays;  during school hours | ≥ 3 days;  ≥ 7 hours;  NR;  15-s | Evenson  (2008) | Boys  (1,078) | NR | -- | -- | -- | 4.9  (4.5) | 33.1  (15.9) | -- |
|  |  |  |  |  | Girls  (1,041) |  | -- | -- | -- | 4.2  (4.3) | 29.9  (12.9) | -- |
|  |  |  |  |  | Total  (2,119) |  | -- | -- | -- | 4.7  (4.4) | 31.7  (15.9) | -- |
| Carlin  (2018) | GT3  (NR) | 7 days;  waking hours | ≥ 3 days;  ≥ 10 hours;  NR;  15-s | Evenson (2008) | Total A  (98) | 450.8 | 310.3  (24.8) | 118.5  (20.3) | 16.8  (6.3) | 5.3  (4.1) | -- | 140.5  (24.8) |
|  |  |  |  |  | Total B (101) | 450.6 | 325.1  (21.8) | 104.8  (18.9) | 16.8  (15.5) | 5.1  (3.8) | -- | 125.5  (22.0) |
| Carlson  (2013) | 7164 / GT1M  (NR) | 7 days;  waking hours | NR;  NR;  ≥ 20 min;  30-s | Freedson  (2005) | Total  (172) | NR | -- | -- | -- | -- | 29.4  (16.2) | -- |
| Carlson  (2015) | GT3X+  (waist) | NR;  during school hours | NR;  ≥ 4 hours;  ≥ 60 min;  5-s | Evenson  (2008) | Total  (1322) | 350.0 | -- | -- | -- | -- | 25.5  (11.3) | -- |
| Carlson  (2017) | 7164/GT1M/GT3X  (Left iliac) | 7 days;  NR | ≥1 valid school day and ≥1 valid nonschool day;  ≥ 8 hours;  ≥ 60 min;  30-s | Evenson  (2008) | Total  (528) | NR | -- | -- | -- | -- | 23.2  (15.1) | -- |
| First Author (Year) | Measure | | | | Sample  (n) | Average valid wear-time during setting (min) | ST | LPA | MPA | VPA | MVPA | TPA |
|  | Device;  (Wear location) | Number wear Days;  Wear duration | Valid days;  Valid hours;  Non-wear time;  Epoch length | Cutpoint |  |  | Mean (SD) | Mean (SD) | Mean (SD) | Mean (SD) | Mean (SD) | Mean (SD) |
| Carson  (2013) | 7164 / GT1M  (Right hip) | 7 days;  waking hours | ≥ 3 days;  ≥ 10 hours;  ≥ 60 min;  30-s | Matthews  (2008) | Girls A  (655) | NR | 243.1  (35.7) | -- | -- | -- | -- | -- |
|  |  |  |  |  | Girls B  (655) |  | 256.7  (34.4) | -- | -- | -- | -- | -- |
| Carson  (2014) | GT3XE-Plus / GT1M  (Right hip) | 5 weekdays;  waking hours | NR;  ≥ 10 hours/ day and ≥ 3 hours during school time;  ≥ 30 min;  5-s | Evenson  (2008) | Boys A  (99) | 362.4 | 261.9  (67.9) | -- | -- | -- | 21.7  (12.5) | -- |
|  |  |  |  |  | Boys B  (58) | 377.3 | 260.9  (53.0) | -- | -- | -- | 23.8  (12.4) | -- |
|  |  |  |  |  | Girls A  (132) | 341.9 | 250.7  (72.1) | -- | -- | -- | 18.0  (10.1) | -- |
|  |  |  |  |  | Girls B  (62) | 378.8 | 264.9  (49.8) | -- | -- | -- | 22.2  (8.9) | -- |
| Centeio  (2014) | GT3X+  (Right hip) | 3 weekdays;  during school hours | NR;  NR;  NR;  15-s | Freedson  (1998) | Total  (344) | 397.0 | -- | -- | -- | -- | 7.3  (7.4) | -- |
| Cinemre  (2015) | GT3X-BT  (Right hip) | 5 weekdays;  during school hours | NR;  NR;  ≥ 20 min;  5-s | Freedson  (2005) | Boys  (40) | 415.0 | 279.0  (NR) | 35.0  (NR) | 80.0  (NR) | 21.0  (NR) | 101.2  (17.8) | -- |
| Cohen  (2008) | 7164  (NR) | 6 days (4x2);  NR | 4 weekdays and  during school hours;  NR;  NR | Puyau  (2002) | Girls  (1,566) | NR | -- | 103.5  (NR) | -- | -- | 33.8  (NR) | -- |
| First Author (Year) | Measure | | | | Sample  (n) | Average valid weartime during setting (min) | ST | LPA | MPA | VPA | MVPA | TPA |
|  | Device;  (Wear location) | Number wear Days;  Wear duration | Valid days;  Valid hours;  Non-wear time;  Epoch length | Cutpoint |  |  | Mean (SD) | Mean (SD) | Mean (SD) | Mean (SD) | Mean (SD) | Mean (SD) |
| Costa  (2017) | GT3X+  (Right hip) | 2 weekdays;  during school hours | 1 day;  NR;  NR;  15-s | Evenson  (2008) | Total  (571) | NR | 132.6  (23.6)^b^ | -- | -- | -- | -- | -- |
|  |  |  |  |  | Boys (309) |  | 137.5  (25.4)^b^ | -- | -- | -- | -- | -- |
|  |  |  |  |  | Girls  (262) |  | 126.9  (21.0)^b^ | -- | -- | -- | -- | -- |
| Cradock  (2014) | GT3X/GT1M or 7164  (Hip) | 5 weekday;  during school hours | NR;  ≥ 3 hours;  60 consecutive minutes of zero counts, allowing for up to 2 minutes of counts below 100;  10-s | Freedson (2005) | Total A  (192) | 338.2 | 183.7  (76.2)^b^ | -- | -- | 6.7  (15.2)^b^ | 20.8  (33.2)^b^ | -- |
|  |  |  |  |  | Total B  (201) | 329.8 | 182.0  (73.7)^b^ | -- | -- | 3.8  (15.0)^b^ | 16.4  (34.0)^b^ | -- |
| Decelis  (2014a) | GT3X  (Right hip) | 5 days (3x2);  NR | ≥ 3 days;  ≥ 10 hours;  ≥60 min;  10-s | Evenson  (2008) | Total  (769) | NR | -- | -- | -- | -- | 18.1  (8.1) | -- |
|  |  |  |  |  | Boys (412) |  | -- | -- | -- | -- | 20.8  (8.8) | -- |
|  |  |  |  |  | Girls  (399) |  | -- | -- | -- | -- | 15.2  (6.3) | -- |
| First Author (Year) | Measure | | | | Sample  (n) | Average valid weartime during setting (min) | ST | LPA | MPA | VPA | MVPA | TPA |
|  | Device;  (Wear location) | Number wear Days;  Wear duration | Valid days;  Valid hours;  Non-wear time;  Epoch length | Cutpoint |  |  | Mean (SD) | Mean (SD) | Mean (SD) | Mean (SD) | Mean (SD) | Mean (SD) |
| Decelis  (2014b) | GT3X  (NR) | 5 weekdays;  waking hours | ≥ 3 days;  ≥ 8.3 hours;  ≥60 min;  10-s | Puyau  (2002) | Boys A  (53) | NR | 266.9  (52.5) | 54.8 (14.2) | -- | -- | 21.0 (10.1) | -- |
|  |  |  |  |  | Boys B (22) |  | 260.3 (64.1) | 52.9 (15.8) | -- | -- | 17.6 (6.8) | -- |
|  |  |  |  |  | Boys C  (19) |  | 273.0 (56.8) | 47.9 (17.0) | -- | -- | 13.3 (7.5) | -- |
|  |  |  |  |  | Girls A (53) |  | 298.7 (49.6) | 38.0 (10.4) | -- | -- | 11.8 (5.7) | -- |
|  |  |  |  |  | Girls B (25) |  | 263.3 (57.4) | 36.4 (8.9) | -- | -- | 13.2 (6.7) | -- |
|  |  |  |  |  | Girls C (15) |  | 279.1 (62.8) | 36.8 (12.1) | -- | -- | 8.1 (3.0) | -- |
| Engelen  (2013) | GT3X  (Left hip) | 5 weekdays;  during school hours | ≥ 3 days;  ≥ 75% during school time;  ≥ 10 min;  15-s | Evenson  (2008) | Total A  (108) | 336.3 | 171.6  (41.3)^b^ | 140.1  (31.9)^b^ | -- | -- | 24.8  (27.0)^b^ | -- |
|  |  |  |  |  | Total B  (113) | 348.5 | 174.4  (42.7)^b^ | 146.5  (31.8)^b^ | -- | -- | 27.4  (31.5)^b^ | -- |
| First Author (Year) | Measure | | | | Sample  (n) | Average valid weartime during setting (min) | ST | LPA | MPA | VPA | MVPA | TPA |
|  | Device;  (Wear location) | Number wear Days;  Wear duration | Valid days;  Valid hours;  Non-wear time;  Epoch length | Cutpoint |  |  | Mean (SD) | Mean (SD) | Mean (SD) | Mean (SD) | Mean (SD) | Mean (SD) |
| Fairclough  (2007) | GT1M  (Right hip) | 4 weekdays;  waking hours | NR;  ≥ 10.1 hours;  ≥ 20 min;  5-s | Nilsson  (2002) | Boys  (31) | NR | -- | -- | -- | -- | 32.8  (9.6) | -- |
|  |  |  |  |  | Girls  (27) |  | -- | -- | -- | -- | 25.4  (9.6) | -- |
| Fairclough  (2012) | GT1M  (Right hip) | 7 days;  waking hours | ≥ 3 weekdays;  ≥ 10 hours;  ≥ 20 min;  5-s | Trost (1998) Eukland (2007) | Total A  (98) | NR | -- | -- | 17.6  (4.9) | 10.8  (4.0) | -- | -- |
|  |  |  |  |  | Boys A  (58) |  | -- | -- | 18.9  (4.7) | 11.2  (4.2) | -- | -- |
|  |  |  |  |  | Girls  (40) |  | -- | -- | 15.6  (4.4) | 10.2  (3.6) | -- | -- |
|  |  |  |  |  | Total B  (125) |  | -- | -- | 14.8  (4.4) | 8.5  (3.9) | -- | -- |
|  |  |  |  |  | Boys B  (41) |  | -- | -- | 15.0  (3.8) | 9.0  (3.1) | -- | -- |
|  |  |  |  |  | Girls B  (84) |  | -- | -- | 14.7  (4.6) | 8.2  (4.3) | -- | -- |
| Farmer  (2017) | GT3X  (Right hip) | 7 days;  24 hours | ≥ 3 days;  ≥ 8 hours valid awake hours;  ≥ 20 min;  15-s | Evenson  (2008) | Total A  (348) | NR | -- | -- | -- | -- | 29.0  (10.0) | -- |
|  |  |  |  |  | Total B  (356) |  | -- | -- | -- | -- | 30.0  (10.0) | -- |
| Gao  (2017) | GT3X  (Right hip) | NR;  8am to 10pm | NR;  NR;  NR;  15-s | Evenson  (2008) | Total A  (138) | 414.4 | 249.7  (NR) | 143.9  (NR) | -- | -- | 20.8  (NR) | -- |
|  |  |  |  |  | Total B  (138) |  | 263.4  (NR) | 146.5 (NR) | -- | -- | 18.3  (NR) | -- |
| First Author (Year) | Measure | | | | Sample  (n) | Average valid weartime during setting (min) | ST | LPA | MPA | VPA | MVPA | TPA |
|  | Device;  (Wear location) | Number wear Days;  Wear duration | Valid days;  Valid hours;  Non-wear time;  Epoch length | Cutpoint |  |  | Mean (SD) | Mean (SD) | Mean (SD) | Mean (SD) | Mean (SD) | Mean (SD) |
| Goh  (2014) | GT1M/GT3X  (NR) | 4 weekdays;  NR | NR;  NR;  NR;  15-s | Evenson  (2008) | Total  (64) | NR | -- | -- | 18.6  (4.4) | 14.8  (5.5) | 33.4  (8.8) | -- |
| Guinhouya  (2009) | 7164  (NR) | 2 days;  waking hours | ≥ 1 day;  ≥11.2 hrs;  ≥ 20 min;  60-s | Puyau  (2002) | Boys  (50) | NR | -- | -- | -- | -- | 29.9  (19.9) | -- |
|  |  |  |  |  | Girls  (43) |  | -- | -- | -- | -- | 17.7  (11.1) | -- |
| Hamer (2017) | GT3X  (Waist) | 7 days;  waking hours | ≥ 1 weekday;  ≥8.3 hours;  ≥ 60 min, allowing for 2 minutes of none zero interruptions;  60-s | ST < 100 CPM,  Light PA 100- 3,000 CPM,  MVPA > 3,000 CPM | Total  (62) | 359.5 | 187.2  (32.3) | 158.7  (29.5) | -- | -- | 13.6  (7.5) | -- |
|  |  |  |  |  | Total (169) | 360.5 | 168.3  (32.2) | 178.1  (31.1) | -- | -- | 14.1  (7.6) | -- |
| Harding (2015) | GT1M  (Waist) | 7 days;  waking hours | NR;  ≥ 60% each segment;  ≥ 60 min, allowing for 2 minutes of none zero interruptions;  NR | Evenson  (2008) | Total A  (363) | 397.7 | 263.9  (36.2) | 108.1  (24.4) | -- | -- | 26.3  (14.9) | -- |
| First Author (Year) | Measure | | | | Sample  (n) | Average valid weartime during setting (min) | ST | LPA | MPA | VPA | MVPA | TPA |
|  | Device;  (Wear location) | Number wear Days;  Wear duration | Valid days;  Valid hours;  Non-wear time;  Epoch length | Cutpoint |  |  | Mean (SD) | Mean (SD) | Mean (SD) | Mean (SD) | Mean (SD) | Mean (SD) |
| Harrington  (2016) | GT1M  (Right hip) | 7 days;  waking hours | ≥ 1 weekday;  NR;  ≥ 10 min;  5-s | Eukland  (2003) | Total A  (301) | 342.7 | -- | -- | -- | -- | 28.2  (9.9) | -- |
| Herrick (2012) | GT1M  (NR) | 3 weekdays;  NR | NR;  ≥ 1 hour;  ≥ 20 min;  30-s | Evenson  (2008) | Total A  (52) | NR | -- | -- | -- | -- | 19.9  (12.2) | -- |
|  |  |  |  |  | Total B  (48) |  | -- | -- | -- | -- | 24.1  (10.5) | -- |
| Hubbard (2016) | GT3X/ GT3X+  (Right hip) | 7 days;  waking hours | ≥ 3 days;  ≥ 10 hours;  ≥ 60 min;  30-s | Evenson (2008) | Total (453) | 385.0 | 213.9  (37.3) | 143.8  (32.6) | -- | -- | 18.1  (8.1) | -- |
| Kim  (2017) | Polar  (Non-dominant wrist) | 6 weeks;  during school hours and afterschool | NR;  NR;  NA;  30-s | Freedson (2005) | Total  (75) | 450.0 | 204.5  (57.3)^b^ | 203.9  (50.8)^b^ | -- | -- | 41.6  (15.5)^b^ | -- |
| Kriemler (2010) | 7164  (Hip) | 5 weekdays;  NR | ≥ 2 weekdays and at least one day PE;  ≥ 12 hours;  ≥ 15 min;  60-s | MVPA > 2,000 CPM | Total A  (205) | 281.4 ^a^ | -- | -- | -- | -- | 37.0  (14.0) | -- |
|  |  |  |  |  | Total B  (297) | 281.5 ^a^ | -- | -- | -- | -- | 38.0  (16.0) | -- |
| Kulik  (2015) | GT3X+  (NR) | 3 weekdays;  during school hours | NR;  NR;  NR;  15-s | Freedson (1998) | Total (347) | NR | -- | -- | -- | -- | 7.3  (7.3) | -- |
| First Author (Year) | Measure | | | | Sample  (n) | Average valid weartime during setting (min) | ST | LPA | MPA | VPA | MVPA | TPA |
|  | Device;  (Wear location) | Number wear Days;  Wear duration | Valid days;  Valid hours;  Non-wear time;  Epoch length | Cutpoint |  |  | Mean (SD) | Mean (SD) | Mean (SD) | Mean (SD) | Mean (SD) | Mean (SD) |
| Kwon  (2015) | GT1M/GT3X  (Right hip) | 4 weekdays;  during school hours | ≥3 days;  ≥ 6 hours;  ≥ 60 min, allowing for 2 minutes of none zero interruptions;  5-s | Evenson (2008) | Boys (252) | 390.0 | 228.0  (48.5)^b^ | -- | -- | -- | 19.9  (10.5)^b^ | -- |
|  |  |  |  |  | Girls  (286) |  | 246.0  (32.3)^b^ | -- | -- | -- | 11.1  (6.4)^b^ | -- |
| Lee  (2016) | GT3X  (NR) | 5 days;  NR | 5 days (3x2);  ≥ 12 hours;  NR;  15-s | Evenson  (2008) | Total  (261) | NR | -- | -- | -- | -- | 20.7  (10.1) | -- |
|  |  |  |  |  | Boys  (127) |  | -- | -- | -- | -- | 24.7  (11.7) | -- |
|  |  |  |  |  | Girls  (134) |  | -- | -- | -- | -- | 17.4  (6.9) | -- |
| Lewis  (2016) | GT3X+  (Right hip) | 7 days;  24 hours | ≥ 4 days, at least 1 weekend day;  ≥10 hours;  NR;  60-s | Treuth (2004) | Total  (491) | NR | -- | -- | -- | -- | 17.9  (11.1) | -- |
|  |  |  |  |  | Boys  (225) |  | -- | -- | -- | -- | 23.1  (12.2) | -- |
|  |  |  |  |  | Girls  (266) |  | -- | -- | -- | -- | 13.6  (7.7) | -- |
| Lin  (2012) | GT1M  (NR) | NR;  NR | ≥ 3 days, at least 1 weekend day;  NR;  NR;  NR | Freedson  (2005) | Boys A  (18) | NR | -- | -- | -- | -- | 72.8  (20.4) | -- |
|  |  |  |  |  | Boys B  (18) |  | -- | -- | -- | -- | 66.0  (19.8) | -- |
| First Author (Year) | Measure | | | | Sample  (n) | Average valid weartime during setting (min) | ST | LPA | MPA | VPA | MVPA | TPA |
|  | Device;  (Wear location) | Number wear Days;  Wear duration | Valid days;  Valid hours;  Non-wear time;  Epoch length | Cutpoint |  |  | Mean (SD) | Mean (SD) | Mean (SD) | Mean (SD) | Mean (SD) | Mean (SD) |
| Long  (2013) | 7164  Right hip) | 7 days;  waking hours | ≥ 4 days;  ≥ 10 hours;  ≥ 60 min, allowing for 2 minutes of none zero interruptions;  60-s | Troiano  (2008)  Trost  (2002) | Boys A  (393) | NR | -- | -- | -- | -- | 36.9  (41.6)^b^ | -- |
|  |  |  |  |  | Boys B  (873) |  | -- | -- | -- | -- | 17.7  (20.6) ^b^ | -- |
|  |  |  |  |  | Girls A  (423) |  | -- | -- | -- | -- | 28.3  (16.4) ^b^ | -- |
|  |  |  |  |  | Girls B  (859) |  | -- | -- | -- | -- | 10.3  (14.6) ^b^ | -- |
| Madsen  (2013) | GT1M / GT3X  NR | 7 days;  during school hours | NR;  NR;  ≥ 20 min;  15-s | Evenson  (2008) | Total  (156) | 480.0 | -- | -- | -- | -- | 19.4  (9.6) | -- |
| Madsen  (2015) | GT1M / GT3X  NR | 5 weekdays;  NR | NR;  NR;  ≥ 20 min;  15-s | Evenson  (2008) | Total A  (147) | 370.0 | 248.7  (28.8) | -- | -- | -- | 21.8  (8.2) | -- |
|  |  |  |  |  | Total B  (303) |  | 240.9  (29.9) | -- | -- | -- | 22.1  (8.8) | -- |
| Magnusson  (2011) | GT1M  NR | 7 days;  waking hours | 2 weekdays;  ≥ 85% of 6 hours school day and ≥ 10 hours per day;  NR;  60-s | Eukland (2004) | Total A  (96) | NR | -- | -- | -- | -- | 32.2  (11.6) | -- |
|  |  |  |  |  | Boys A  (39) |  | -- | -- | -- | -- | 40.7  (9.5) | -- |
|  |  |  |  |  | Girls A  (57) |  | -- | -- | -- | -- | 26.8  (15.3) | -- |
|  |  |  |  |  | Total B  (100) |  | -- | -- | -- | -- | 36.3  (14.3) | -- |
|  |  |  |  |  | Boys B  (48) |  | -- | -- | -- | -- | 45.2  (16.1) | -- |
|  |  |  |  |  | Girls B  (52) |  | -- | -- | -- | -- | 30.2  (12.6) | -- |
| First Author (Year) | Measure | | | | Sample  (n) | Average valid weartime during setting (min) | ST | LPA | MPA | VPA | MVPA | TPA |
|  | Device;  (Wear location) | Number wear Days;  Wear duration | Valid days;  Valid hours;  Non-wear time;  Epoch length | Cutpoint |  |  | Mean (SD) | Mean (SD) | Mean (SD) | Mean (SD) | Mean (SD) | Mean (SD) |
| Martin  (2017) | GT3X/ GT3X+  NR | 5 weekdays;  during school hours | 5 weekdays;  > 80% School day;  NR;  15-s | Evenson (2008) | Total A  (91) | 332.5 | 214.7  (24.8) | 96.3  (20.6) | -- | -- | 21.5  (6.2) | -- |
|  |  |  |  |  | Total B  (95) | 334.0 | 222.7  (22.5) | 92.1  (19.6) | -- | -- | 19.2  (6.7) | -- |
| Mooses  (2016) | GT3X  (waist) | NR;  waking hours | ≥ 4 weekdays;  ≥ 10 hours;  ≥ 20 min;  15-s | Evenson (2008) | Total A  (244) | NR | -- | -- | -- | -- | 18.2  (12.9) | -- |
|  |  |  |  |  | Total B  (228) |  | -- | -- | -- | -- | 12.9  (10.4) | -- |
| Morton  (2016) | GT1M  (NR) | 7 days;  during waking hours | ≥ 2 days;  ≥80 % of the duration of school day;  ≥ 10 mins;  5-s | Ridgers (2012) and above 2,000 CPM | Total A  (321) | 384.3 | 272.9  (25.5) | 80.6  (16.3) | -- | -- | 30.7  (10.7) | -- |
|  |  |  |  |  | Total B  (315) | 392.2 | 300.1  (29.8) | 64.6  (18.8) | -- | -- | 27.4  (13.7) | -- |
| Nettlefold  (2011) | GT1M  (Waist) | 5 days;  waking hours | ≥ 3 days;  ≥ 10 hours;  NR;  15-s | Trost  (2002) | Boys  (181) | 366.1 | 246.2  (25.0) | 56.4  (11.9) | -- | -- | 63.5  (21.4) | -- |
|  |  |  |  |  | Girls  (198) | 367.1 | 260.1  (28.8) | 54.1  (13.3) | -- | -- | 52.9  (16.8) | -- |
| Nielsen  (2012) | 7164  (Lower back) | 4 days (2 x 2);  waking hours | ≥ 3 days;  ≥ 8 hours;  ≥ 10 min;  10-s | MPA (2,500 to  5,000 CPM  VPA >5,000 CPM | Total  (518) | NR | -- | -- | 21.1  (9.2) | 10.7  (7.9) | -- | -- |
|  |  |  |  |  | Boys  (266) |  | -- | -- | 23.6  (9.4) | 12.1  (8.3) | -- | -- |
|  |  |  |  |  | Girls  (252) |  | -- | -- | 18.4  (8.1) | 9.2  (7.2) | -- | -- |
| Nilsson  (2009) | 7164  (Waist) | 4 days  (2x2);  waking hours | ≥ 3 days (2x1);  ≥ 10 hours;  NR;  60-s | Puyau (2002)  MVPA > 2,000 CPM | Boys A  (141) | 352.0 | 115.0  (38.0) | -- | -- | -- | 49.0  (25.0) | -- |
|  |  |  |  |  | Girls A  (160) |  | 128.0  (38.0) | -- | -- | -- | 29.0  (18.0) | -- |
|  |  |  |  |  | Boys B  (151) |  | 146.0  (42.0) | -- | -- | -- | 38.0  (22.0) | -- |
|  |  |  |  |  | Girls B  (141) |  | 153.0  (40.0) | -- | -- | -- | 26.0  (14.0) | -- |
|  |  |  |  |  | Boys C  (151) |  | 122.0  (40.0) | -- | -- | -- | 46.0  (23.0) | -- |
|  |  |  |  |  | Girls C  (148) |  | 138.0  (36.0) | -- | -- | -- | 35.0  (17.0) | -- |
|  |  |  |  |  | Boys D  (152) |  | 128.0  (33.0) | -- | -- | -- | 58.0  (27.0) | -- |
|  |  |  |  |  | Girls D  (140) |  | 140.0  (31.0) | -- | -- | -- | 44.0  (19.0) | -- |
|  |  |  |  |  | Boys E  (86) | 401.0 | 205.0  (51.0) | -- | -- | -- | 26.0  (17.0) | -- |
|  |  |  |  |  | Girls E  (112) |  | 218.0  (45.0) | -- | -- | -- | 21.0  (14.0) | -- |
|  |  |  |  |  | Boys F  (79) |  | 206.0  (47.0) | -- | -- | -- | 40.0  (23.0) | -- |
|  |  |  |  |  | Girls F  (83) |  | 217.0  (45.0) | -- | -- | -- | 28.0  (14.0) | -- |
|  |  |  |  |  | Boys G  (100) |  | 186.0  (53.0) | -- | -- | -- | 43.0  (21.0) | -- |
|  |  |  |  |  | Girls G (172) |  | 227.0  (48.0) | -- | -- | -- | 30.0  (16.0) | -- |
|  |  |  |  |  | Boys H  (58) |  | 206.0  (38.0) | -- | -- | -- | 41.0  (19.0) | -- |
|  |  |  |  |  | Girls H  (80) |  | 228.0  (43.0) | -- | -- | -- | 33.0  (13.0) | -- |
| First Author (Year) | Measure | | | | Sample  (n) | Average valid wear-time during setting (min) | ST | LPA | MPA | VPA | MVPA | TPA |
|  | Device;  (Wear location) | Number wear Days;  Wear duration | Valid days;  Valid hours;  Non-wear time;  Epoch length | Cutpoint |  |  | Mean (SD) | Mean (SD) | Mean (SD) | Mean (SD) | Mean (SD) | Mean (SD) |
| Noonan  (2017) | GENEActiv  (Left wrist) | 7 days;  waking hours | ≥ 3 days (2x1);  ≥ 10 hours;  NR;  1-s | Hildebrand (2014) | Total A  (107) | NR | -- | 165.5  (34.0) | -- | -- | 16.7  (7.1) | -- |
|  | GT3X+  (right hip) |  |  |  | Total B (83) |  | -- | 61.7  (29.0) | -- | -- | 9.8  (4.4) | -- |
| Pau  (2017) | GT3X  (dominant Wrist) | 7 days;  24 hours | NR;  ≥ 16 hours;  ≥ 60 min;  10-s | Crouter (2015) | Total A  (90) | 299.8 | 195.3  (27.5) | 71.6  (16.5) | -- | -- | 32.9  (14.1) | -- |
|  |  |  |  |  | Total B  (79) | 479.9 | 293.3  (50.3) | 125.7  (30.1) | -- | -- | 60.9  (27.7) | -- |
| Pearce  (2018) | GT3X+  (Right hip) | 7 days;  waking hours | ≥ 1 day;  ≥ 9 hours;  ≥ 60 min and 15,000 counts per minute;  10-s | Hänggi (2012) | Total  (70) | 333.2 | -- | -- | -- | -- | 24.2  (25.1)^b^ | -- |
| Piipari  (2016) | GT1M / GT3X/ GT3X+  (Right hip) | 5 weekdays;  during school hours/ | ≥ 1 day;  ≥ 80% school hours;  ≥ 30 min;  NR | Evenson  (2008) | Total  (61) | 387.0 | 250.2  (37.4) | 125.7  (30.3) | 13.5  (7.1) | 6.4  (5.1) | 20.0  (15.2) | -- |
|  |  |  |  |  | Boys  (29) |  | 239.9  (37.4) | 129.6  (30.3) | 15.4  (7.1) | 7.7  (5.1) | 23.5  (12.6) | -- |
|  |  |  |  |  | Girls  (32) |  | 258.6  (39.9) | 122.5  (31.6) | 11.6  (7.1) | 5.1  (6.4) | 16.8  (8.1) | -- |
|  |  | 7 days;  (NR) |  |  | Total  (139) | 283.2 | 171.8  (16.9) | 87.3  (13.6) | 15.1  (4.7) | 8.9  (4.2) | 24.1  (8.3) | -- |
|  |  |  |  |  | Boys  (64) |  | 166.6  (16.5) | 89.6  (12.2) | 16.5  (4.2) | 10.3  (4.7) | 26.7  (9.0) | -- |
|  |  |  |  |  | Girls  (75) |  | 176.5  (16.0) | 85.4  (14.6) | 13.6  (3.7) | 8.0  (4.2) | 21.8  (6.9) | -- |
| First Author (Year) | Measure | | | | Sample  (n) | Average valid weartime during setting (min) | ST | LPA | MPA | VPA | MVPA | TPA |
|  | Device;  (Wear location) | Number wear Days;  Wear duration | Valid days;  Valid hours;  Non-wear time;  Epoch length | Cutpoint |  |  | Mean (SD) | Mean (SD) | Mean (SD) | Mean (SD) | Mean (SD) | Mean (SD) |
| Pizarro  (2017) | GT3X+  (Waist) | 7 days;  waking time | NR;  NR;  ≥ 60 min;  30-s | Evenson  (2008) | Total  (374) | NR | -- | -- | -- | -- | 30.5  (NR) | -- |
|  |  |  |  |  | Boys  (201) |  | -- | -- | -- | -- | 37.0  (NR) | -- |
|  |  |  |  |  | Girls  (173) |  | -- | -- | -- | -- | 24.7  (NR) | -- |
| Price  (2013) | GT1M  (NR) | 7 days;  waking time | ≥ 3 days;  ≥ 10 hours;  ≥ 20 min;  30-s | PAGA  (2008) | Total (667) | NR | -- | -- | -- | -- | 37.6  (27.4) | -- |
| Pulsford  (2013) | GT1M  (NR) | 7 days;  waking hours | ≥ 2 days (1x1);  ≥ 10 hours;  ≥ 60 min;  10-s | Treuth  (2004) | Total  (629) | 340.9 | 218.4  (35.0) | -- | -- | -- | -- | -- |
| Rainham  (2012) | GT1M  (Waist) | 8 days;  waking hours | ≥1 day;  ≥ 8 hours;  NR;  30-s | NR | Total A  (91) | NR | -- | -- | -- | -- | 45.7  (45.2) | -- |
|  |  |  |  |  | Total B  (102) |  | -- | -- | -- | -- | 18.6  (28.0) | -- |
|  |  |  |  |  | Total C  (123) |  | -- | -- | -- | -- | 29.8  (39.7) | -- |
| Ramirez-Rico  (2014) | GT1M  (Right hip) | 7 days;  waking hours | ≥ 3 days (2x1);  ≥ 9 hours;  ≥ 20 min;  5-s | Evenson  (2008) | Total A  (187) | 363.1 | - | -- | 14.3  (5.4)^b^ | 4.7  (4.0)^b^ | 19.6  (8.1)^b^ | -- |
|  |  |  |  |  | Total B  (180) | 386.0 | -- | -- | 11.0  (5.3)^b^ | 8.6  (5.3)^b^ | 19.6  (9.3)^b^ | -- |
| First Author (Year) | Measure | | | | Sample  (n) | Average valid wear-time during setting (min) | ST | LPA | MPA | VPA | MVPA | TPA |
|  | Device;  (Wear location) | Number wear Days;  Wear duration | Valid days;  Valid hours;  Non-wear time;  Epoch length | Cutpoint |  |  | Mean (SD) | Mean (SD) | Mean (SD) | Mean (SD) | Mean (SD) | Mean (SD) |
| Resaland  (2016) | GT3X+  (Right hip) | 7 days;  waking hours | ≥ 3 weekdays;  ≥ 3 hours between 9am-2pm;  ≥ 20 min;  10-s | Evenson  (2008)  Trost (2011) | Total A (497) | NR | 179.0  (20.0) | -- | -- | -- | 29.0  (11.0) | -- |
|  |  |  |  |  | Total B  (566) |  | 178.0  (19.0) | -- | -- | -- | 28.0  (10.0) | -- |
| Ridgers  (2010) | GT1M  (Right hip) | 7 days;  waking hours | ≥3 days (1 weekend);  ≥ 9 hours;  ≥ 20 min;  5-s | 4km·h^-1^ | Total  (110) | NR | -- | -- | -- | -- | 30.0  (24.1) | -- |
| Riley  (2016) | GT3X  (NR) | NR;  during school hours | NR;  ≥ 5 hours and 50 min of 60min of math class;  NR;  15-s | Evenson  (2008) | Total A  (98) | NR | -- | -- | -- | -- | 27.8  (35.6)^b^ | -- |
|  |  |  |  |  | Total B  (142) |  | -- | -- | -- | -- | 30.9  (35.2)^b^ | -- |
| Ross  (2013) | 7164  (Right hip) | 7 days;  NR | ≥ 1 day;  NR;  ≥ 20 min;  30-s | Treuth  (2004) | Girls A  (1,387) | NR | -- | -- | -- | -- | 25.1  (16.3) | -- |
|  |  |  |  |  | Girls B  (479) |  | -- | -- | -- | -- | 74.9  (49.9) | -- |
| Rush  (2012) | Actical  (NR) | 3 weekdays;  during school hours | ≥ 3 weekdays;  NR;  NR;  15-s | Puyau  (2004) | Total  (47) | 300.0 | 140.7  (37.1) | 92.4  (21.3) | 63.9  (18.3) | 3.8  (3.1) | 67.5  (20.4) | -- |
|  |  |  |  |  | Boys  (19) |  | 143.3  (33.6) | 89.1  (19.5) | 64.7  (15.5) | 3.6  (3.6) | 68.4  16.7) | -- |
|  |  |  |  |  | Girls  (28) |  | 138.9  (39.9) | 94.7  (22.4) | 63.3  (20.3) | 3.9  (2.8) | 67.3  (22.4) | -- |
| First Author (Year) | Measure | | | | Sample  (n) | Average valid weartime during setting (min) | ST | LPA | MPA | VPA | MVPA | TPA |
|  | Device;  (Wear location) | Number wear Days;  Wear duration | Valid days;  Valid hours;  Non-wear time;  Epoch length | Cutpoint |  |  | Mean (SD) | Mean (SD) | Mean (SD) | Mean (SD) | Mean (SD) | Mean (SD) |
| Sayers  (2012) | GT1M  (Right hip) | 7 days;  waking hours | NR;  NR;  NR;  30-s | Trost  (2001) | Total A  (38) | NR | -- | -- | -- | -- | 18.3  (8.4) | -- |
|  |  |  |  |  | Total B  (39) |  | -- | -- | -- | -- | 19.2  (7.2) | -- |
| Schneider  (2017) | GT3X  (Left hip) | 7 days;  waking hours | ≥ 4 days (≥ 1 weekend day);  ≥ 8 hours;  NR;  NR | Freedson  (2005) | Total A  (32) | NR | -- | -- | -- | -- | 30.3  (12.0) | -- |
|  |  |  |  |  | Total B  (31) |  | -- | -- | -- | -- | 28.2  (8.8) | -- |
|  |  |  |  |  | Total C  (34) |  | -- | -- | -- | -- | 29.0  (8.9) | -- |
|  |  |  |  |  | Total D  (29) |  | -- | -- | -- | -- | 28.8  (10.1) | -- |
| Sigmund  (2014) | ActiTrainer  (Waist) | 7 days;  24 hours | NR;  NR;  ≥ 60 min;  15-s | Evenson  (2008) | Total  (338) | 280.7^a^ | 146.3  (30.5) | 122.7  (30.5) | -- | -- | 13.7  (10.7) | -- |
| Silva  (2018) | ActivPAL / GT3X  (Right hip) | 7 days;  waking hours | ≥ 4 days;  ≥ 10 hours;  Choi (2011);  15-s | Evenson  (2008) | Total A  (27) | NR | -- | 157.6  (35.4) | -- | -- | 36.5  (14.8) | -- |
|  |  |  |  |  | Total B  (22) |  | -- | 162.9  (36.1) | -- | -- | 46.7  (23.1) | -- |
| Sprengeler  (2017) | GT3X+/ GT1M / ActiTrainer  (Right hip) | 7 days;  waking hours | ≥ 3 days;  ≥ 10 hours;  ≥ 90 min;  3-s | Evenson  (2008) | Total  (207) | NR | 271.0  (38.0) | 103.0  (22.0) | -- | -- | 41.0  (14.0) | -- |
| First Author (Year) | Measure | | | | Sample  (n) | Average valid weartime during setting (min) | ST | LPA | MPA | VPA | MVPA | TPA |
|  | Device;  (Wear location) | Number wear Days;  Wear duration | Valid days;  Valid hours;  Non-wear time;  Epoch length | Cutpoint |  |  | Mean (SD) | Mean (SD) | Mean (SD) | Mean (SD) | Mean (SD) | Mean (SD) |
| Steele  (2010) | GT1M  (Right hip) | 7 days;  waking hours | ≥ 3 days (2x1);  ≥ 8.3 hours;  ≥ 10 min;  5-s | ST < 100 CPM, MVPA >2,000 CPM,  VPA > 4,000 CPM | Total  (1,568) | NR | 236.4  (21.2) | -- | -- | 10.2  (4.4) | -- | -- |
|  |  |  |  |  | Boys  (701) |  | 229.1  (20.8) | -- | -- | 12.6  (5.8) | -- | -- |
|  |  |  |  |  | Girls  (867) |  | 242.4  (19.6) | -- | -- | 8.2  (4.0) | -- | -- |
| Stewart  (2017) | GT3X  (Right hip) | 7 days;  NR | ≥ 1 day;  ≥ 8 hours;  ≥ 60 mins;  15-s | Evenson  (2008) | Total A  (126) | 252.0 | 188.0  (25.7) ^b^ | -- | -- | -- | 14.5  (8.3)^b^ | -- |
|  |  |  |  |  | Total B  (60) | 285.0 | 189.0  (27.0)^b^ | -- | -- | -- | 15.8  (8.3)^b^ | -- |
| Strugnell  (2016) | GT3X / GT3X+  (Right hip) | NR;  waking hours | ≥ 3 days;  ≥ 10 hours;  ≥ 60 min;  15-s | Romanzini  (2014) | Boys  (130) | 376.2 | 212.7  (30.2) | 101.7  (18.7) | -- | -- | 61.8  (17.1) | -- |
|  |  |  |  |  | Girls  (168) | 375.6 | 227.3  (31.8) | 103.1  (22.7) | -- | -- | 45.2  (15.0) | -- |
| Sutherland  (2017) | GT3X+  (Waist) | 7 days;  waking hours | ≥ 3 days;  ≥ 10 hours;  ≥ 30 min;  15-s | Evenson  (2008) | Total A  (492) | NR | -- | -- | 22.8  (17.0) | 12.4  (7.4) | 35.2  (24.9) | -- |
|  |  |  |  |  | Total B  (497) |  | -- | -- | 21.7  (17.0) | 10.6  (9.1) | 32.3  (26.3) | -- |
| Suzuki  (2018) | HJA-350IT  (Waist) | ≥ 7 days;  waking hours | NR;  NR;  ≥ 20 min;  10-s | ST ≤1.5  LPA >1.5 - < 3.0  MPA ≥ 3.0 - <6.0  VPA ≥ 6.0  MET | Total (39) | 473.6 | 188.8  (57.4) | 236.7  (36.0) | 40.6  (12.0) | 7.5  (4.0) | 48.1  (14.9) | -- |
| First Author (Year) | Measure | | | | Sample  (n) | Average valid weartime during setting (min) | ST | LPA | MPA | VPA | MVPA | TPA |
|  | Device;  (Wear location) | Number wear Days;  Wear duration | Valid days;  Valid hours;  Non-wear time;  Epoch length | Cutpoint |  |  | Mean (SD) | Mean (SD) | Mean (SD) | Mean (SD) | Mean (SD) | Mean (SD) |
| Taylor  (2011) | GT3X  (Waist) | 5 weekdays;  waking hours | NR;  > 8 hours;  ≥ 20 min;  60-s | Puyau  (2004) | Boys  (230) | 343.0 | -- | -- | -- | -- | 17.0  (10.0) | -- |
|  |  |  |  |  | Girls  (197) | 342.0 | -- | -- | -- | -- | 11.0  (7.0) | -- |
| Taylor  (2017) | GT9X  (Non-dominant wrist) | 7 days;  24h | ≥ 3 days  ≥ 16 hours;  ≥ 60 min;  1-s | Hildbrand (2014) | Boys  (92) | 376.7 | 198.4  (31.3) | 157.5  (27.4) | -- | -- | 20.9  (8.7) | -- |
|  |  |  |  |  | Girls  (94) |  | 210.4  (32.6) | 151.9  (27.9) | -- | -- | 14.3  (7.2) | -- |
| Ting  (2015) | Actitrainer  (Right hip) | 5 days (3x2);  waking hours | 5 days;  ≥ 8 hours;  ≥ 10 min, allowance of 1 to 2 min of counts between 0–100;  NR | Evenson  (2008) | Total  (225) | 347.4 | 218.4  (72.5) | 114.6  (39.3) | -- | -- | 14.4  (9.9) | -- |
|  |  |  |  |  | Boys (118) | 335.3 | 202.0  (72.2) | 116.1  (42.5) | -- | -- | 17.1  (10.8) | -- |
|  |  |  |  |  | Girls  (107) | 360.8 | 236.4  (112.9) | 35.7  (11.0) | -- | -- | 11.4  (7.8) | -- |
| Van Sluijs  (2011) | GT1M  (Right hip) | 7 days;  waking hours | ≥ 2 weekdays;  ≥ 8.3 hours;  ≥ 10 min;  5-s | Trost (1998)  Eston  (1998) | Total  (1,908) | NR | 297.4  (26.5) | -- | 29.7  (8.4) | 13.9  (7.0) | -- | -- |
|  |  |  |  |  | Boys  (841) |  | 288.1  (25.6) | -- | 33.2  (8.1) | 17.0  (7.6) | -- | -- |
|  |  |  |  |  | Girls  (1,067) |  | 304.7  (24.8) | -- | 26.9  (7.6) | 11.5  (5.4) | -- | -- |
| Van Stralen  (2014) | ActiTrainer / GT3X / GT1M  (waist) | 6 days;  waking hours | NR;  at least 50% of school time;  ≥ 20 min;  15-s | Treuth  (2004) | Total  (1,025) | 354.0 | 209.0  (35.0) | -- | -- | -- | 16.0  (9.0) | -- |
|  |  |  |  |  | Total A  (191) | 391.0 | 231.0  (32.0) | -- | -- | -- | 17.0  (8.0) | -- |
|  |  |  |  |  | Total B  (201) | 325.0 | 182.0  (24.0) | -- | -- | -- | 16.0  (8.0) | -- |
|  |  |  |  |  | Total C  (178) | 299.0 | 188.0  (27.0) | -- | -- | -- | 14.0  (7.0) | -- |
|  |  |  |  |  | Total D  (190) | 376.0 | 215.0  (28.0) | -- | -- | -- | 13.0  (7.0) | -- |
|  |  |  |  |  | Total E  (265) | 370.0 | 233.0  (34.0) | -- | -- | -- | 21.0  (10.0) | -- |
|  |  |  |  |  | Boys  (496) | 354.0 | 223.0  (24.7) | -- | -- | -- | 17.7  (10.6) | -- |
|  |  |  |  |  | Boys A  (94) | 391.0 | 238.5  (27.3) | -- | -- | -- | 19.5  (7.8) | -- |
|  |  |  |  |  | Boys B  (98) | 325.0 | 198.2  (22.7) | -- | -- | -- | 19.5  (9.7) | -- |
|  |  |  |  |  | Boys C (88) | 299.0 | 194.3  (23.9) | -- | -- | -- | 14.9  (8.9) | -- |
|  |  |  |  |  | Boys D  (96) | 376.0 | 244.4  (30.0) | -- | -- | -- | 15.0  (7.5) | -- |
|  |  |  |  |  | Boys E (120) | 370.0 | 225.7  (22.2) | -- | -- | -- | 22.2  (7.4) | -- |
|  |  |  |  |  | Girls  (511) | 354.0 | 237.1  (28.3) | -- | -- | -- | 14.1  (7.0) | -- |
|  |  |  |  |  | Girls A  (96) | 391.0 | 254.1  (27.3) | -- | -- | -- | 15.6  (11.7) | -- |
|  |  |  |  |  | Girls B  (103) | 325.0 | 214.5  (26.0) | -- | -- | -- | 13.0  (6.5) | -- |
|  |  |  |  |  | Girls C  (88) | 299.0 | 209.3  (20.9) | -- | -- | -- | 11.9  (5.9) | -- |
|  |  |  |  |  | Girls D (94) | 376.0 | 255.6  (30.0) | -- | -- | -- | 11.2  (7.5) | -- |
|  |  |  |  |  | Girls E  (130) | 370.0 | 244.2  (25.9) | -- | -- | -- | 18.5  (11.1) | -- |
| First Author (Year) | Measure | | | | Sample  (n) | Average valid weartime during setting (min) | ST | LPA | MPA | VPA | MVPA | TPA |
|  | Device;  (Wear location) | Number wear Days;  Wear duration | Valid days;  Valid hours;  Non-wear time;  Epoch length | Cutpoint |  |  | Mean (SD) | Mean (SD) | Mean (SD) | Mean (SD) | Mean (SD) | Mean (SD) |
| Vanhelst  (2017) | GT1M  (Waist) | 7 days;  waking hours | ≥ 3 days;  ≥ 10 hours;  NR;  60-s | Vanhelst (2010) | Total A  (1,230) | 330.0 | 290.2  (52.3) | -- | -- | -- | 18.4  (13.8) | -- |
|  |  |  |  |  | Total B  (794) | 454.8 | 379.1  (68.3) | -- | -- | -- | 28.9  (19.9) | -- |
| Verloigne  (2015) | GT1M/ GT3X/ GT3X+/ ActiTrainer  (Right hip) | 7 days;  waking hours | ≥ 2 weekdays;  ≥ 10 min;  ≥ 60 min;  15-s | Ridgers (2012) | Total A  (193) | NR | 179.4  (NR) | -- | -- | -- | -- | -- |
|  |  |  |  |  | Boys A  (69) |  | 175.2  (NR) | -- | -- | -- | -- | -- |
|  |  |  |  |  | Girls A  (124) |  | 177.2  (NR) | -- | -- | -- | -- | -- |
|  |  |  |  |  | Total B  (161) |  | 189.9  (NR) | -- | -- | -- | -- | -- |
|  |  |  |  |  | Boys B  (75) |  | 186.3  (NR) | -- | -- | -- | -- | -- |
|  |  |  |  |  | Girls B  (86) |  | 189.6  (NR) | -- | -- | -- | -- | -- |
| Weaver  (2016) | GT3X+  (Waist) | 5 weekdays;  during school hours | ≥ 1 day;  ≥ 5 hours, at least 70% school hours;  NR;  NR | Matthews (2008)  Evenson (2008) | Boys A  (150) | 412.3 | 245.6  (76.1) | 132.3  (51.9) | -- | -- | 34.4  (17.8) | -- |
|  |  |  |  |  | Girls A (173) | 414.6 | 251.0 (70.6) | 133.2  (55.5) | -- | -- | 30.4  (14.9) | -- |
|  |  |  |  |  | Boys B  (150) | 405.2 | 276.3  (75.5) | 101.6  (58.5) | -- | -- | 27.3  (18.6) | -- |
|  |  |  |  |  | Girls B  (173) | 406.7 | 276.6  (62.9) | 106.6  (52.7) | -- | -- | 23.5  (16.6) | -- |
| First Author (Year) | Measure | | | | Sample  (n) | Average valid weartime during setting (min) | ST | LPA | MPA | VPA | MVPA | TPA |
|  | Device;  (Wear location) | Number wear Days;  Wear duration | Valid days;  Valid hours;  Non-wear time;  Epoch length | Cutpoint |  |  | Mean (SD) | Mean (SD) | Mean (SD) | Mean (SD) | Mean (SD) | Mean (SD) |
| Weaver  (2018a) | GT3X+  (Waist) | 4 weekdays;  during school hours | ≥ 1 day;  at least 70% school hours;  NR;  5-s | Matthews (2008)  Evenson (2008) | Boys A  (417) | 353.0 | -- | -- | -- | -- | 21.8  (11.6) | 103.6  (25.1) |
|  |  |  |  |  | Boys B  (417) |  | -- | -- | -- | -- | 22.8  (12.4) | 99.6  (26.7) |
|  |  |  |  |  | Girls A  (378) |  | -- | -- | -- | -- | 19.4  (9.9) | 99.8  (22.9) |
|  |  |  |  |  | Girls B  (378) |  | -- | -- | -- | -- | 21.5  (11.6) | 99.8  (24.5) |
| Weaver  (2018b) | GT3X+  (Waist) | 3 weekdays;  during school hours | ≥ 1 day;  ≥ 5 hours;  NR;  NR | Matthews (2008)  Evenson (2008) | Boys A  (57) | 370.8 | 167.9  (35.9) | -- | -- | -- | 31.5  (8.8) | -- |
|  |  |  |  |  | Boys B  (70) |  | 182.4  (53.0) | -- | -- | -- | 37.7  (16.3) | -- |
|  |  |  |  |  | Girls A  (40) |  | 174.6  (30.7) | -- | -- | -- | 28.5  (10.3) | -- |
|  |  |  |  |  | Girls B  (62) |  | 191.3  (47.4) | -- | -- | -- | 24.8  (12.2) | -- |
| Wells  (2014) | GT3X+ / GT1M  (Waist) | 3 weekdays;  during school hours | NR;  NR;  NR;  30-s | Evenson  (2008) | Total A  (64) | 355.0 | 194.3  (12.7) | 124.5  (7.3) | 19.2  (4.0) | 17.7  (4.3) | 36.7  (7.6) | -- |
|  |  |  |  |  | Total B  (60) |  | 196.0  (13.2) | 122.9  (7.7) | 18.3  (3.8) | 17.7  (4.4) | 36.0 (7.9) | -- |
| Wilson  (2017) | Geneactive  (Non- dominant wrist) | 7 days;  24 hours | 3 – 6 days;  NR;  NR;  15-s | Phillips (2013) | Total A  (19) | NR | -- | -- | 31.2  (11.1) | 5.5  (3.3) | 36.7  (7.6) | -- |
|  |  |  |  |  | Total B  (19) |  | -- | -- | 33.8  (11.3) | 8.6 (4.0) | 36.0  (7.9) | -- |
| First Author (Year) | Measure | | | | Sample  (n) | Average valid weartime during setting (min) | ST | LPA | MPA | VPA | MVPA | TPA |
|  | Device;  (Wear location) | Number wear Days;  Wear duration | Valid days;  Valid hours;  Non-wear time;  Epoch length | Cutpoint |  |  | Mean (SD) | Mean (SD) | Mean (SD) | Mean (SD) | Mean (SD) | Mean (SD) |
| Zimmo  (2017) | GT3X-BT  (Non-dominant wrist) | 5 weekdays;  during school hours | ≥ 3 days;  ≥ 4 hours;  ≥ 10 min of null activity readings, with 120 consecutives of counts less than 100;  5-s | Chandler (2015) | Total A  (91) | NR | -- | -- | -- | -- | 24.5  (8.7) | -- |
|  |  |  |  |  | Boys A  (47) |  | -- | -- | -- | -- | 26.0  (8.6) | -- |
|  |  |  |  |  | Girls A  (44) |  | -- | -- | -- | -- | 23.1  (8.8) | -- |
|  |  |  |  |  | Total B  (92) |  | -- | -- | -- | -- | 31.8  (16.1) | -- |
|  |  |  |  |  | Boys B  (39) |  | -- | -- | -- | -- | 42.7  (16.1) | -- |
|  |  |  |  |  | Girls B  (53) |  | -- | -- | -- | -- | 23.7  (10.3) | -- |
| **Afterschool** | | | | | | | | | | | | |
| Arundell  (2013) | 7164  (Right hip) | 8 days;  waking hours | ≥ 3 weekdays;  ≥ 50% during afterschool;  ≥ 20 min;  60-s | Trost  (2011)  Ridgers  (2012) | Boys A  (313) | 150.3^a^ | 41.7  (14.5) | 70.9  (12.6) | 25.1  (9.3) | 13.2  (8.4) | -- | -- |
|  |  |  |  |  | Girls A  (295) |  | 41.8  (13.9) | 74.2  (11.8) | 23.6  (7.8) | 10.2  (6.8) | -- | -- |
|  |  |  |  |  | Boys B  (656) | 150.9^a^ | 56.7  (17.7) | 73.4  (14.2) | 14.6  (7.7) | 6.8  (6.6) | -- | -- |
|  |  |  |  |  | Girls B  (789) |  | 57.2  (16.1) | 78.5  (13.7) | 11.4  (5.9) | 4.6  (4.9) | -- | -- |
| Beets  (2010, 2012) | GT1M  (Right hip) | 4 weekdays;  during afterschool hours | ≥ 1 day;  ≥ 1 hour;  ≥ 500 steps;  ≥ 10 min;  60-s / 5-s | Puyau  (2002) | Boys  (120) | 108.5 | 63.7 (25.1)^b^ | 26.8  (11.2)^b^ | -- | -- | 18.4  (11.1)^b^ | 45.8  (12.0)^b^ |
|  |  |  |  |  | Girls  (125) | 112.7 | 74.7  (24.6)^b^ | 24.9  (10.4)^b^ | -- | -- | 13.3  (8.8)^b^ | 37.3  (12.3)^b^ |
| First Author (Year) | Measure | | | | Sample  (n) | Average valid weartime during setting (min) | ST | LPA | MPA | VPA | MVPA | TPA |
|  | Device;  (Wear location) | Number wear Days;  Wear duration | Valid days;  Valid hours;  Non-wear time;  Epoch length | Cutpoint |  |  | Mean (SD) | Mean (SD) | Mean (SD) | Mean (SD) | Mean (SD) | Mean (SD) |
| Beets  (2013) | GT1M  (Waist) | 4 weekdays;  during afterschool hours | ≥ 1 days;  ≥ 50 min;  ≥ 10 min;  5-s | Puyau  (2002)  Matthews  (2011) | Boys  (393) | 124.5 | 63.1  (41.6) ^b^ | 47.4  (25.7)^b^ | 12.9  (15.8) ^b^ | 1.0  (1.9) ^b^ | 13.9  (17.8) ^b^ | 61.4  (41.6) ^b^ |
|  |  |  |  |  | Girls  (392) |  | 70.4  (39.0) ^b^ | 44.1  (23.4)^b^ | 9.3  (17.5)^b^ | 0.8  (1.9) ^b^ | 10.1  (19.5) ^b^ | 54.1  (39.0)^b^ |
| Beets  (2014) | Actigraph  (NR) | 4 weekdays;  during afterschool hours | ≥ 1 day;  ≥ 1 hour;  NR;  5-s | Evenson  (2008)  Matthews  (2008) | Boys  (204) | 115.7 | 52.2  (21.3) | 40.7  (15.2) | 12.2  (5.9) | 10.5  (6.7) | 22.7  (11.6) | -- |
|  |  |  |  |  | Girls  (186) | 113.8 | 58.0  (23.8) | 38.5  (15.8) | 9.7  (5.2) | 7.8  (5.7) | 17.5  (10.1) | -- |
|  |  |  |  |  | Boys  (241) | 115.7 | 46.0  (21.5) | 47.0  (19.4) | 14.6  (7.4) | 13.4  (9.3) | 28.0  (14.8) | -- |
| Beets  (2015a) | GT3X  (NR) | 4 weekdays;  during afterschool hours | ≥ 1 day;  ≥ 1 hour;  NR;  5-s | Evenson  (2008) | Boys  (430) | 127.1 | 62.5  (24.8) | 40.4  (15.8) | -- | -- | 19.8  (11.8) | 60.3  (24.5) |
|  |  |  |  |  | Girls  (382) | 122.7 | 71.0  (29.4) | 40.2  (15.7) | -- | -- | 15.8  (10.3) | 56.0  (22.9) |
| Beets  (2015b) | GT3X+  (NR) | 4 weekdays;  during afterschool hours | ≥ 1 day;  ≥ 1 hour;  NR;  5-s | Evenson  (2008)  Matthews  (2011) | Boys A  (339) | 135.6 | 68.5  (23.8) | 43.6  (15.7) | 11.6  (6.0) | 11.9  (7.9) | 23.5  (13.3) | -- |
|  |  |  |  |  | Girls A  (288) | 139.1 | 77.2  (27.7) | 44.1  (15.5) | 9.4  (4.4) | 8.4  (5.7) | 17.9  (9.5) | -- |
|  |  |  |  |  | Boys B  (352) | 126.7 | 60.7  (17.9) | 41.3  (13.0) | 12.4  (5.0) | 12.4  (6.5) | 24.8  (10.7) | -- |
|  |  |  |  |  | Girls B  (318) | 119.9 | 62.5  (19.0) | 39.5  (14.8) | 9.3  (4.6) | 8.6  (5.3) | 18.0 (9.3) | -- |
| Beets  (2018) | GT3X  (Hip) | 4 weekdays;  during afterschool hours | ≥ 1 day;  ≥ 1 hour;  NR;  5-s | Evenson  (2008)  Matthews  (2011) | Total  (1,078) | 127.0 | 64.3  (24.6) | 41.2  (17.9) | 11.0 (6.6) | 10.4  (7.7) | 21.4  (13.5) | -- |
| First Author (Year) | Measure | | | | Sample  (n) | Average valid weartime during setting (min) | ST | LPA | MPA | VPA | MVPA | TPA |
|  | Device;  (Wear location) | Number wear Days;  Wear duration | Valid days;  Valid hours;  Non-wear time;  Epoch length | Cutpoint |  |  | Mean (SD) | Mean (SD) | Mean (SD) | Mean (SD) | Mean (SD) | Mean (SD) |
| Cradock  (2016) | GT3X/GT1M/7164  (Hip) | 5 weekdays;  during afterschool hours | ≥ 2 days;  ≥ 1 hour;  ≥ 60 min allowing for up to 2 minutes bellow 100 counts;  60-s | Freedson  (2005) | Total A  (220) | 139.9 | 42.8  (56.3)^b^ | -- | -- | 13.7  (32.6)^b^ | 30.4  (54.8) ^b^ | -- |
|  |  |  |  |  | Total B  (182) |  | 48.6  (51.2) ^b^ | -- | -- | 8.9  (29.6) ^b^ | 23.8  (49.9) ^b^ | -- |
| Dzewaltowski  (2010) | GT1M  (Right hip) | 6 weekdays;  during afterschool hours | NR;  NR;  NR;  30-s | Freedson (2005) | Total A  (80) | NR | 39.9  (39.5)^b^ | -- | -- | 7.1  (15.2)^b^ | 17.7  (26.8)^b^ | -- |
|  |  |  |  |  | Total A  (39) |  | 32.3  (31.2)^b^ | -- | -- | 7.3  (11.8)^b^ | 20.9  (21.2)^b^ | -- |
|  |  |  |  |  | Total B  (81) |  | 39.2  (38.7)^b^ | -- | -- | 5.9  (14.4)^b^ | 17.5  (25.2)^b^ | -- |
|  |  |  |  |  | Total B  (46) |  | 46.9  (31.8)^b^ | -- | -- | 2.4  (12.2)^b^ | 11.3  (20.3)^b^ | -- |
| Gesell  (2013) | GT1M  (NR) | 5 weekdays;  during afterschool hours | ≥ 3 days;  NR;  NR;  10-s | Pate  (2006) | Total  (83) | 107.2 | -- | -- | -- | -- | 29.4  (15.3) | 82.9  (11.2) |
| Herrick  (2012) | GT1M  (NR) | 3 weekdays;  NR | NR;  ≥ 1 hour;  ≥ 20 min;  30-s | Evenson  (2008) | Total A  (52) | 180.0 | -- | -- | -- | -- | 20.6  (13.4) | -- |
|  |  |  |  |  | Total B  (48) | 173.3 | -- | -- | -- | -- | 22.7  (14.8) | -- |
| Huberty  (2014) | GT1M  (Right hip) | 4 days;  during afterschool hours | NR;  ≥ 1 hour;  NR;  5-s | Evenson  (2008) | Girls  (182) | 103.0 | 68.3  (29.6) | 26.4  (15.5) | 4.7  (3.4) | 3.6  (3.4) | 8.4  (6.5) | -- |
| First Author (Year) | Measure | | | | Sample  (n) | Average valid weartime during setting (min) | ST | LPA | MPA | VPA | MVPA | TPA |
|  | Device;  (Wear location) | Number wear Days;  Wear duration | Valid days;  Valid hours;  Non-wear time;  Epoch length | Cutpoint |  |  | Mean (SD) | Mean (SD) | Mean (SD) | Mean (SD) | Mean (SD) | Mean (SD) |
| Madsen  (2013) | GT1M/GT3X  (NR) | 7 days;  during session hours | NR;  NR;  ≥ 10 min;  15-s | Evenson  (2008) | Total  (156) | 180.0 | -- | -- | -- | -- | 17.3  (9.7) |  |
| Trost  (2008) | GT1M  (Right hip) | 6 weekdays;  during afterschool hours | NR;  ≥ 1 hour;  ≥ 10 min;  30-s | Freedson  (2005) | Total  (140) | 93.8 | 42.6  (7.0) | 40.8  (7.0) | 13.4  (4.7) | 6.9  (3.5) | 20.3  (9.4) | -- |
|  |  |  |  |  | Boys  (79) |  | 42.4  (10.6) | 37.7  (7.1) | 12.1  (5.3) | 4.1  (3.5) | 17.2  (9.7) | -- |
|  |  |  |  |  | Girls  (61) |  | 42.8  (10.1) | 43.8  (6.2) | 12.1  (4.6) | 4.1  (3.1) | 17.3  (8.5) | -- |
| **Sport** | | | | | | | | | | | | |
| Behrens  (2015) | GT3X  (NR) | NR;  during session hours | NR;  NR;  NR;  30-s / 15-s | Evenson  (2008) | Total  (104) | 37.2 | 4.4  (4.4) | 16.9  (5.9) | 8.4  (2.9) | 7.2  (4.4) | 15.6  (5.2) | -- |
| Behrens  (2016) | GT3X  (NR) | NR;  during session hours | NR;  ≥ 15 min;  ≥ 10 min;  30-s / 15-s | Evenson  (2008) | Total  (205) | 54.2 | 10.4  (6.1) | 28.2  (8.3) | 10.3  (4.4) | 5.3  (4.3) | 15.6  (6.6) | -- |
| Cain  (2015) | GT3X  (Waist) | NR;  During session hours | NR;  NR;  NR;  15-s | Freedson (2005)  Evenson (2008) | Total A (103) | NR | 12.9 (1.9) | 14.7  (0.8) | 15.2 (0.8) | 5.4  (0.7) | 20.6  (1.1) | -- |
|  |  |  |  |  | Total B (51) |  | 15.2 (1.5) | 15.2 (1.9) | 13.0 (1.3) | 3.4 (1.7) | 16.1 (1.9) | -- |
|  |  |  |  |  | Total C  (76) |  | 16.7  (1.5) | 23.9 (1.2) | 9.5  (0.9) | 3.1  (0.7) | 12.7  (1.4) | -- |
|  |  |  |  |  | Total D (34) |  | 14.9 (3.2) | 26.0  (2.4) | 10.7  (1.7) | 0.7 (1.3) | 11.0 (2.4) | -- |
| First Author (Year) | Measure | | | | Sample  (n) | Average valid weartime during setting (min) | ST | LPA | MPA | VPA | MVPA | TPA |
|  | Device;  (Wear location) | Number wear Days;  Wear duration | Valid days;  Valid hours;  Non-wear time;  Epoch length | Cutpoint |  |  | Mean (SD) | Mean (SD) | Mean (SD) | Mean (SD) | Mean (SD) | Mean (SD) |
| Cohen  (2014) | GT1M / GT3X  (Right hip) | NR;  during session hours | NR;  NR;  NR;  1-s / 15-s | Evenson  (2008) | Total  (29) | 62.5 | 15.2  (6.2) | -- | -- | -- | 23.0  (6.8) | -- |
| Fenton  (2015) | GT3X  (NR) | 7 days;  waking hours | NR;  ≥ 8 hours;  ≥ 30 min, allowing 1 min of counts < 100;  15-s | Evenson  (2008) | Total  (109) | 106.7 | -- | -- | -- | 27.7  (14.5) | 51.5  (17.9) | -- |
| Guagliano  (2013) | GT3X  (Right hip) | NR;  during session hours | NR;  NR;  NR;  5-s | Freedson  (2005) | Total  (82) | 100.0 | 38.9  (14.4) | 30.5  (9.7) | 15.7  (5.7) | 14.9  (6.5) | 30.6  (9.5) | -- |
|  |  |  |  |  | Total  (82) |  | 30.1  (10.6) | 36.0  (8.4) | 17.1  (5.0) | 16.7  (6.4) | 33.8  (7.7) | -- |
|  |  |  |  |  | Total A  (36) |  | 34.9  (9.9) | 33.7  (4.7) | 15.9  (3.8) | 15.5  (5.9) | 31.4  (9.0) | -- |
|  |  |  |  |  | Total A  (36) |  | 31.6  (7.2) | 34.7  (4.8) | 15.4  (2.8) | 18.2  (4.4) | 33.7  (5.3) | -- |
|  |  |  |  |  | Total A  (36) |  | 27.1  (5.9) | 27.8  (4.6) | 15.5  (2.6) | 29.4  (6.6) | 45.0  (6.3) | -- |
|  |  |  |  |  | Total B  (27) |  | 48.9  (11.3) | 20.6  (4.1) | 12.8  (4.2) | 17.7  (5.0) | 30.5  (8.0) | -- |
|  |  |  |  |  | Total B  (27) |  | 32.7  (11.6) | 31.6  (6.7) | 16.4  (3.5) | 19.1  (6.0) | 35.5  (8.1) | -- |
|  |  |  |  |  | Total C  (19) |  | 32.2  (18.3) | 38.6  (10.8) | 19.6  (7.9) | 9.6  (6.6) | 29.2  (12.4) | -- |
|  |  |  |  |  | Total C  (19) |  | 23.5  (12.1) | 45.0  (9.3) | 21.1  (7.6) | 10.4  (6.2) | 31.5  (10.5) | -- |
| First Author (Year) | Measure | | | | Sample  (n) | Average valid weartime during setting (min) | ST | LPA | MPA | VPA | MVPA | TPA |
|  | Device;  (Wear location) | Number wear Days;  Wear duration | Valid days;  Valid hours;  Non-wear time;  Epoch length | Cutpoint |  |  | Mean (SD) | Mean (SD) | Mean (SD) | Mean (SD) | Mean (SD) | Mean (SD) |
| Guagliano  (2015) | GT3X+  (NR) | 2 days;  during session hours | 2 days;  NR;  NR;  NR | Evenson  (2008) | Total A  (38) | 49.1 | 25.6  (9.8) | 12.8  (4.9) | 4.1  (2.4) | 6.5  (4.9) | 10.6  (7.3) | -- |
|  |  |  |  |  | Total B  (38) | 52.2 | 27.3  (9.2) | 13.6  (5.5) | 4.2  (1.8) | 7.0  (7.3) | 11.2  (6.1) | -- |
| Guagliano  (2017) | ActiCal  (Right hip) | 9 sessions;  during sessions hours | NR;  NR;  NR;  15-s | Puyau (2002) | Total A  (32) | 35.0 | 10.3  (3.1) | 14.1  (2.3) | 9.8  (2.5) | 0.7  (0.6) | 10.5  (2.7) | -- |
|  |  |  |  |  | Total B  (32) |  | 11.3  (3.1) | 13.9  (2.3) | 8.5  (2.5) | 0.7  (0.6) | 9.6  (2.7) |  |
| Kim  (2015) | Polar Active  (non-dominant wrist) | 6 weeks;  during school hours and afterschool | NR;  NR;  NR;  30-s | Freedson (2005) | Total  (75) | 40.0 | 4.7  (2.5) | 13.6  (3.4) | -- | -- | 21.7  (4.3) | -- |
| Leek  (2011) | GT1M  (Waist) | NR;  during sessions hours | NR;  NR;  NR;  15-s | Freedson  (2005) | Total A  (100) | 105.0 | 29.3  (14.1) | 20.3 (16.2) | 25.8  (8.7) | 29.6  (14.0) | -- | -- |
|  |  |  |  |  | Total B  (97) | 102.0 | 29.9  (15.5) | 29.4  (15.3) | 30.2  (16.5) | 10.5  (5.7) | -- | -- |
| Lopez-Castillo  (2015) | GT3X  (NR) | NR;  during session hours | NR;  NR;  NR;  15-s | Freedson  (2005)  Evenson (2008) | Total  (291) | 49.7 | -- | -- | -- | -- | 15.8  (17.3)^b^ | -- |
| O’Neill  (2012) | 7164  (Right hip) | 3 to 4 weeks periods;  during session hours and following week | ≥ 2 days;  NR;  NR;  30-s | Treuth  (2004) | Total  (137) | 62.0 | 13.2  (11.7) | 42.6  (12.8) | 6.2  (3.5) | 4.2  (3.5) | 10.4  (5.8) | -- |
| First Author (Year) | Measure | | | | Sample  (n) | Average valid weartime during setting (min) | ST | LPA | MPA | VPA | MVPA | TPA |
|  | Device;  (Wear location) | Number wear Days;  Wear duration | Valid days;  Valid hours;  Non-wear time;  Epoch length | Cutpoint |  |  | Mean (SD) | Mean (SD) | Mean (SD) | Mean (SD) | Mean (SD) | Mean (SD) |
| Ridley  (2018) | GT3X / GT3X+  (waist) | 2 sessions;  during the practice | ≥ 1 session;  ≥ 75% during the practice;  NR;  15-s | Evenson  (2008) | Boys A  (32) | 90.0 | 11.0 (5.0) | 35.0 (8) | 23.0 (7.0) | 21.0 (8.0) | 41.0  (9.0) | -- |
|  |  |  |  |  | Boys B  (40) | 60.0 | 12.0 (5.0) | 21.0 (4.0) | 10.0 (3.0) | 12.0 (5.0) | 22.0 (6.0) | -- |
|  |  |  |  |  | Girls A  (34) | 90.0 | 18.0 (9.0) | 38.0 (9.0) | 18.0 (5.0) | 16.0 (7.0) | 34.0 (9.0) | -- |
|  |  |  |  |  | Girls B  (35) | 80.0 | 19.0 (6.0) | 29.0 (5.0) | 14.0 (4.0) | 18.0 (4.0) | 32.0 (7.0) | -- |
| Rosenkranz  (2011) | GT1M  (Right hip) | 6 days;  during session hours | NR;  ≥ 0.5 hour;  ≥ 60 mins;  30-s | Freedson  (2005) | Total  (240) | 27.5 | 4.4  (3.0) | -- | -- | -- | 10.7  (4.0) | -- |
| Sacheck  (2011) | 7164  (Right hip) | NR;  during the game | NR;  NR;  NR;  1-s | Puyau  (2002) | Total  (111) | 51.6 | 25.4  (5.7) | -- | -- | -- | 16.9  (4.7) | -- |
|  |  |  |  |  | Boys  (36) |  | 25.9  (4.5) | 10.2  (3.7) | 11.9  (3.0) | 3.9  (2.7) | 15.8  (4.4) | -- |
|  |  |  |  |  | Girls  (75) |  | 25.1  (6.2) | 9.2  (2.6) | 13.7  (3.7) | 3.7  (2.3) | 17.5  (4.7) | -- |
| Schlechter  (2017) | GT1M  (NR) | NR;  during session hours; | NR;  NR;  NR;  15-s | Evenson  (2008) | Total  (111) | 61.5 | 7.7  (5.2)^b^ | -- | -- | 6.8  (5.2)^b^ | 19.9  (12.6)^b^ | -- |
| First Author (Year) | Measure | | | | Sample  (n) | Average valid weartime during setting (min) | ST | LPA | MPA | VPA | MVPA | TPA |
|  | Device;  (Wear location) | Number wear Days;  Wear duration | Valid days;  Valid hours;  Non-wear time;  Epoch length | Cutpoint |  |  | Mean (SD) | Mean (SD) | Mean (SD) | Mean (SD) | Mean (SD) | Mean (SD) |
| Schuna  (2013) | GT3X  (Right hip) | 2 weekdays;  during session hours | ≥ 1 session;  ≥ 15 min;  ≥ 10 mins;  30-s; the age specific  cut points were divided by 2 | Freedson  (2005) | Total (116) | 56.6 | 7.6  (9.6)^b^ | 26.9  (18.3) ^b^ | 18.4  (19.3) ^b^ | 2.9  (9.6) ^b^ | 22.2  (25.8) ^b^ | -- |
|  |  |  |  |  | Boys  (52) |  | 6.6  (7.2)^b^ | 25.3  (12.9) ^b^ | 20.1  (12.9) ^b^ | 3.5  (7.9) ^b^ | 24.7  (18.0) ^b^ | -- |
|  |  |  |  |  | Girls  (64) |  | 8.6  (8.0) ^b^ | 28.5  (14.4) ^b^ | 16.7  (14.4) ^b^ | 2.4  (5.6) ^b^ | 19.7  (20.0) ^b^ | -- |
| Smith  (2016) | GT3X  (Waist) | 7 days;  waking hours | NR;  ≥10 hours;  ≥ 60 min of consecutive zero counts with less  than two consecutive intervals with counts less than or equal to 100;  60-s | Freedson  (2005) | Boys  (455) | 111.0 | 23.3  (42.0) | 43.9  (45.0) | 18.9  (14.0) | 14.4  (14.0) | 33.3  (24.0) | -- |
|  |  |  |  |  | Girls  (555) | 95.2 | 20.9  (23.0) | 31.0  (12.2) | 14.4  (10.5) | 14.4  (11.0) | 22.7  (19.0) | -- |
| **Summer Camp** | | | | | | | | | | | | |
| Baker  (2017) | GT1M  (Right hip) | 4 weekdays;  during summer camp hours | NR;  NR;  NR;  10-s | Freedson  (2005) | Boys  (73) | 480.0 | -- | -- | -- | -- | 61.4 (3.4)^b^ | -- |
|  |  |  |  |  | Girls  (59) |  | -- | -- | -- | -- | 45.6  (3.8)^b^ | -- |
|  |  |  |  |  | Total  (132) |  | -- | -- | -- | -- | 57.7  (26.5)^b^ | -- |
| First Author (Year) | Measure | | | | Sample  (n) | Average valid weartime during setting (min) | ST | LPA | MPA | VPA | MVPA | TPA |
|  | Device;  (Wear location) | Number wear Days;  Wear duration | Valid days;  Valid hours;  Non-wear time;  Epoch length | Cutpoint |  |  | Mean (SD) | Mean (SD) | Mean (SD) | Mean (SD) | Mean (SD) | Mean (SD) |
| Barnett (2018) | GT3X/ GT3X+  (Hip) | 5 weekdays;  During summer camp hours | ≥ 2 days;  ≥ 2 hours;  ≥60 min;  NR | Troiano (2011) | Total A  (40) | 299.0 | -- | -- | -- | 13.4  (NR) | 72.6  (NR) | -- |
|  |  |  |  |  | Total B  (142) | 549.0 | -- | -- | -- | 16.4  (NR) | 85.6  (NR) | -- |
| Beets  (2011) | GT1M / Walk4Life  (Right hip) | NR;  during summer camp hours | NR;  NR;  ≥ 10 min;  5-s | Evenson (2008) | Total A  (149) | 341.0 | -- | -- | -- | -- | 36.9  (21.0) | -- |
|  |  |  |  | Freedson (2005) |  |  | -- | -- | -- | -- | 50.4  (25.5) | -- |
|  |  |  |  | Mattocks (2007) |  |  | -- | -- | -- | -- | 18.8  (13.3) | -- |
|  |  |  |  | Puyau (2002) |  |  | -- | -- | -- | -- | 22.7  (15.1) | -- |
|  |  |  |  | Treuth (2004) |  |  | -- | -- | -- | -- | 25.2  (16.2) | -- |
|  |  |  |  | Freedson (2005) | Total B  (45) |  | -- | -- | -- | -- | 41.5  (22.7) | -- |
|  |  |  |  | Mattocks (2007) |  |  | -- | -- | -- | -- | 17.0  (11.6) | -- |
|  |  |  |  | Puyau (2002) |  |  | -- | -- | -- | -- | 20.9  (13.5) | -- |
|  |  |  |  | Treuth (2004) |  |  | -- | -- | -- | -- | 23.4  (14.6) | -- |
|  |  |  |  | Evenson (2008) | Total C  (72) |  | -- | -- | -- | -- | 37.4  (22.5) | -- |
|  |  |  |  | Freedson (2005) |  |  | -- | -- | -- | -- | 54.9  (26.3) | -- |
|  |  |  |  | Puyau (2002) |  |  | -- | -- | -- | -- | 23.7  (16.5) | -- |
|  |  |  |  | Evenson (2008) | Boys A  (89) |  | -- | -- | -- | -- | 42.5  (21.8) | -- |
|  |  |  |  | Freedson (2005) |  |  | -- | -- | -- | -- | 57.7  (26.3) | -- |
|  |  |  |  | Mattocks (2007) |  |  | -- | -- | -- | -- | 21.5  (14.0) | -- |
|  |  |  |  | Puyau (2002) |  |  | -- | -- | -- | -- | 26.1  (15.8) | -- |
|  |  |  |  | Treuth (2004) |  |  | -- | -- | -- | -- | 29.0  (17.0) | -- |
|  |  |  |  | Evenson (2008) | Girls A  (60) |  | -- | -- | -- | -- | 28.7  (16.8) | -- |
|  |  |  |  | Freedson (2005) |  |  | -- | -- | -- | -- | 39.6  (20.0) | -- |
|  |  |  |  | Mattocks (2007) |  |  | -- | -- | -- | -- | 14.7  (11.0) | -- |
|  |  |  |  | Puyau (2002) |  |  | -- | -- | -- | -- | 17.6  (12.4) | -- |
|  |  |  |  | Treuth (2004) |  |  | -- | -- | -- | -- | 19.6  (13.4) | -- |
|  |  |  |  | Freedson (2005) | Boys B  (25) |  | -- | -- | -- | -- | 43.5  (24.9) | -- |
|  |  |  |  | Mattocks (2007) |  |  | -- | -- | -- | -- | 16.9  (11.1) | -- |
|  |  |  |  | Puyau (2002) |  |  | -- | -- | -- | -- | 21.2  (13.3) | -- |
|  |  |  |  | Treuth (2004) |  |  | -- | -- | -- | -- | 24.0  (14.7) | -- |
|  |  |  |  | Freedson (2005) | Girls B  (20) |  | -- | -- | -- | -- | 39.0  (19.9) | -- |
|  |  |  |  | Mattocks (2007) |  |  | -- | -- | -- | -- | 17.2  (12.6) | -- |
|  |  |  |  | Puyau (2002) |  |  | -- | -- | -- | -- | 20.4  (13.9) | -- |
|  |  |  |  | Treuth (2004) |  |  | -- | -- | -- | -- | 22.6  (14.9) | -- |
|  |  |  |  | Evenson (2008) | Boys C  (46) |  | -- | -- | -- | -- | 44.5  (22.3) | -- |
|  |  |  |  | Freedson (2005) |  |  | -- | -- | -- | -- | 63.9  (24.9) | -- |
|  |  |  |  | Puyau (2002) |  |  | -- | -- | -- | -- | 28.2  (16.8) | -- |
|  |  |  |  | Evenson (2008) | Girls C  (26) |  | -- | -- | -- | -- | 24.8  (16.9) | -- |
|  |  |  |  | Freedson (2005) |  |  | -- | -- | -- | -- | 38.9  (20.7) | -- |
|  |  |  |  | Puyau (2002) |  |  | -- | -- | -- | -- | 14.9  (12.2) | -- |
| Weaver  (2017) | GT3X-BT  (non-dominant wrist) | 4 non-consecutive weekdays within a 6-week period;  during summer camp hours | ≥ 1 day;  ≥ 4 hours;  NR;  5-s | Chandler  (2015)  Chandler  (2018) | Boys A  (522) | 516.0 | 259.0  (69.9) | -- | -- | -- | 98.7  (44.7) | -- |
|  |  |  |  |  | Boys B  (485) | 510.0 | 266.9  (64.1) | -- | -- | -- | 90.4  (37.1) | -- |
|  |  |  |  |  | Girls A  (404) | 516.0 | 275.1  (66.4) | -- | -- | -- | 85.1  (35.1) | -- |
|  |  |  |  |  | Girls B  (419) | 510.0 | 273.0  (66.4) | -- | -- | -- | 77.4  (35.1) | -- |

Legend: ST = Sedentary time; LA = Light physical activity; MPA = Moderate physical activity; VPA = Vigorous physical activity; MVPA = Moderate-to-vigorous physical activity; TPA = Total physical activity. NR = Not reported; CPM = counts per minute; MET = Metabolic equivalent;

^a^ = Information provided by the authors (e-mail); ^b^ = originally presented in standard error or 95%CI. Transformed according Cochrane handbook.
