# Supplementary material for: Physical activity and sedentary time of youth in structured settings: a systematic review and meta-analysis
Source: Int J Behav Nutr Phys Act. 2020 Dec 4;17:160. doi: 10.1186/s12966-020-01054-y (PMC7716454; doi:10.1186/s12966-020-01054-y)
Supplement: Supplementary file 6 — Additional file 6. [file 12966_2020_1054_MOESM6_ESM.docx]

Electronic supplementary material table 3s. Summary of Childcare setting results of meta-regression multivariate models by outcomes.

|  | | | | Mean estimate | | | | | Min/hour estimate | | | | |
| --- | --- | --- | --- | --- | --- | --- | --- | --- | --- | --- | --- | --- | --- |
|  |  |  |  | β | 95% CI | | p | Adjusted  R-squared (%) | β | 95% CI | | p | Adjusted R-squared (%) |
|  | Mean (range) | k | e |  | Lower | Upper |  |  |  | Lower | Upper |  |  |
| **Sedentary time** |  |  |  |  |  |  | **<0.01** | **60.6** |  |  |  | **<0.01** | **18.2** |
| Sex  All (combined estimates)  Boys  Girls |  | 9  9 | 12  12 | Ref.  -36.59  11.72 | -85.38  -37.60 | 12.19  61.12 | 0.13  0.63 |  | Ref.  -2.55  1.91 | -10.40  -5.91 | 5.29  9.77 | 0.51  0.62 |  |
| Age | 4.1 (3.0 – 6.1) | 44 | 86 | 15.40 | -8.39 | 39.20 | 0.20 |  | 1.16 | -2.61 | 4.91 | 0.54 |  |
| Sample size | 155 (15 – 716) | 44 | 86 | -0.09 | -0.19 | 0.003 | 0.06 |  | -0.01 | -0.03 | 8.20 | 0.06 |  |
| Global Region  Oceania/South America/Europe  North America |  | 32 | 63 | Ref.  -13.95 | 45.9 | 18.08 | 0.38 |  | Ref.  -0.52 | -5.55 | 4.50 | 0.83 |  |
| Study design  Other design  Cross-sectional |  | 32 | 60 | Ref.  34.38 | 2.40 | 66.36 | **0.03** |  | Ref.  6.41 | 1.39 | 11.43 | **0.01** |  |
| Accelerometer Brand  ActiCal  ActiGraph |  | 30 | 57 | Ref.  40.11 | 10.71 | 69.50 | **<0.01** |  | Ref.  6.28 | 1.67 | 10.90 | **<0.01** |  |
| Cutpoint  All  Pate  Sirard  Pfeiffer |  | 12  10  7 | 28  16  15 | Ref.  -55.99  57.98  -30.80 | -89.53  20.93  -81.39 | -22.46  95.03  19.78 | **<0.01**  **<0.01**  0.22 |  | Ref.  -7.66  7.72  -2.65 | -13.09  1.72  -10.75 | -2.22  13.73  5.44 | **<0.01**  **0.01**  0.51 |  |
| Weartime | 377.4 (138 – 624) | 44 | 86 | 0.79 | 0.65 | 0.94 | **<0.01** |  | 0.01 | -0.01 | 0.34 | 0.31 |  |
| Risk of bias | 19.3 (14 – 22) | 44 | 86 | 1.77 | 0.54 | 3.01 | **<0.01** |  | 0.33 | 0.13 | 0.52 | **<0.01** |  |
| **Light Physical Activity** |  |  |  |  |  |  | **<0.01** | **42.3** |  |  |  | **<0.01** | **31.8** |
| Sex  All (combined estimates)  Boys  Girls |  | 6  6 | 11  11 | Ref.  -10.13  -5.65 | -27.99  -24.30 | 7.73  13.04 | 0.26  0.54 |  | Ref.  -2.55  1.91 | -3.89  -4.33 | 2.46  2.25 | 0.65  0.52 |  |
| Age | 4.1 (3.0 – 6.1) | 30 | 64 | -23.48 | -38.51 | -8.15 | **<0.01** |  | -3.32 | -5.97 | -0.68 | **0.01** |  |
| Sample size | 155 (15 – 716) | 30 | 64 | 0.14 | 0.08 | 0.21 | **<0.01** |  | 0.02 |  |  | **<0.01** |  |
| Global Region  Oceania/Europe  North America |  | 24 | 54 | Ref.  -7.26 | -28.28 | 13.75 | 0.49 |  | Ref.  -2.22 | -5.94 | 1.50 | 0.23 |  |
| Study design  Other design  Cross-sectional |  | 22 | 46 | Ref.  -0.23 | -18.77 | 18.29 | 0.97 |  | Ref.  -0.67 | -3.95 | 2.60 | 0.68 |  |
| Accelerometer Brand  ActiCal  ActiGraph |  | 10  20 | 20  44 | Ref.  -54.63 | -69.56 | -39.69 | **<0.01** |  | Ref.  -9.05 | -11.70 | -6.40 | **<0.01** |  |
| Cutpoint  All  Pate  Sirard  Pfeiffer |  | 8  8  5 | 21  18  11 | Ref.  -23.40  5.31  1.69 | -38.52  -11.98  -22.95 | -8.28  22.60  26.33 | **<0.01**  0.54  0.89 |  | Ref.  -4.75  2.17  -1.04 | -7.35  -0.84  -5.40 | -2.15  5.18  3.30 | **<0.01**  0.15  0.63 |  |
| Weartime | 368.5 (138 – 624) | 30 | 64 | 0.15 | 0.06 | 0.24 | **<0.01** |  | 0.001 | -0.01 | 0.01 | 0.88 |  |
| Risk of bias | 19.8 (17 – 22) | 30 | 64 | -0.51 | -7.63 | 6.60 | 0.88 |  | -0.43 | -1.66 | 0.79 | 0.48 |  |
| **Moderate Physical Activity** |  |  |  |  |  |  | 0.73 | -9.4 |  |  |  | 0.83 | -12.4 |
| Age | 4.4 (3.7 – 6.1) | 16 | 24 | -2.90 | -8.93 | 3.13 | 0.32 |  | -0.50 | -1.57 | 0.56 | 0.33 |  |
| Sample size | 190.3 (20 – 593) | 16 | 24 | 0.0004 | -0.02 | 0.03 | 0.97 |  | 0.0002 | -0.005 | 0.005 | 0.91 |  |
| Weartime | 324.3 (158.2 – 501) | 16 | 24 | 0.01 | -0.04 | 0.08 | 0.55 |  | -0.004 | -0.01 | 0.007 | 0.44 |  |
| Risk of bias | 19.2 (17 – 22) | 16 | 24 | 0.57 | -3.38 | 4.53 | 0.76 |  | 0.19 | -0.50 | 0.89 | 0.57 |  |
| **Vigorous Physical Activity** |  |  |  |  |  |  | **<0.01** | **45.2** |  |  |  | **<0.01** | **22.5** |
| Age | 4.3 (3.5 – 6.3) | 17 | 36 | -1.76 | -5.60 | 2.07 | 0.35 |  | -0.26 | -1.08 | 0.55 | 0.51 |  |
| Sample size | 191.4 (20 – 716) | 17 | 36 | 0.02 | 0.003 | 0.03 | 0.02 |  | 0.004 | 0.0007 | 0.008 | 0.02 |  |
| Weartime | 286.4 (138 – 501) | 17 | 36 | 0.01 | -0.01 | 0.05 | 0.26 |  | 0.001 | -0.005 | 0.009 | 0.61 |  |
| Risk of bias | 18.3 (14 -22) | 17 | 36 | -3.36 | -4.86 | 1.86 | **<0.01** |  | -0.70 | -102 | -0.38 | **<0.01** |  |
| **Moderate to Vigorous Physical Activity** | |  |  |  |  |  | 0.06 | 5.6 |  |  |  | **<0.01** | **10.9** |
| Sex  All (combined estimates)  Boys  Girls |  | 11  11 | 14  14 | Ref.  3.05  -2.79 | -3.30  -8.75 | 6.84  3.24 | 0.45  0.52 |  | Ref.  0.86  -0.01 | -0.59  -1.48 | 2.32  1.45 | 0.24  0.98 |  |
| Age | 4.1 (3.0 – 5.8) | 46 | 90 | 4.16 | -1.50 | 9.82 | 0.14 |  | 0.67 | -0.26 | 1.62 | 0.15 |  |
| Sample size | 158.0 (15 – 716) | 46 | 90 | 0.01 | -0.002 | 0.04 | 0.07 |  | 0.003 | 0.0001 | 0.073 | **0.04** |  |
| Global Region  Oceania/Europe  North America |  | 32 | 65 | Ref.  -7.79 | -15.50 | -0.07 | **0.04** |  | Ref.  -1.04 | -2.36 | 0.26 | 0.11 |  |
| Study design  All (combined estimates)  Cross-sectional |  | 33 | 64 | Ref.  -5.02 | -12.26 | 2.21 | 0.17 |  | Ref.  -1.13 | -2.37 | 0.09 | 0.06 |  |
| Accelerometer Brand  ActiCal  ActiGraph |  | 41 | 66 | Ref.  10.01 | 2.73 | 17.30 | **<0.01** |  | Ref.  1.60 | 0.36 | 2.84 | **0.01** |  |
| Cutpoint  All  Pate  Sirard  Pfeiffer |  | 15  9  11 | 32  19  22 | Ref  17.61  - 14.32  -16.29 | 11.64  -27.25  -23.38 | 23.58  -1.39  -9.20 | **<0.01**  **<0.01**  **0.03** |  | Ref.  2.86  -2.44  -2.60 | 1.83  -3.68  -4.79 | 3.90  -1.20  -0.41 | **<0.01**  **<0.01**  **0.02** |  |
| Weartime | 373.9 (138 – 624) | 46 | 90 | 0.02 | -0.008 | 0.06 | 0.13 |  | -0.005 | -0.01 | 0.0054 | 0.07 |  |
| Risk of bias | 19.2 (14 – 22) | 46 | 90 | -0.82 | -2.85 | 1.20 | 0.42 |  | -0.22 | -0.56 | 0.14 | 0.19 |  |
| **Total Physical Activity** |  |  |  |  |  |  | **<0.01** | **31.1** |  |  |  | **<0.01** | **18.3** |
| Sex  All (combined estimates)  Boys  Girls |  | 8  8 | 12  12 | Ref.  21.7  -6.87 | -1.12  -30.15 | 34.42  16.40 | 0.06  0.55 |  | Ref.  4.34  -0.06 | 0.98  -3.59 | 6.52  3.46 | **0.01**  0.97 |  |
| Age |  | 26 | 59 | -31.9 | -47.2 | -16.6 | **<0.01** |  | -4.63 | -6.88 | 2.37 | **<0.01** |  |
| Sample size |  | 26 | 59 | 0.01 | -0.06 | 0.10 | 0.67 |  | 0.001 | -0.01 | 0.01 | 0.82 |  |
| Accelerometer Brand  ActiCal  ActiGraph |  | 17 | 39 | Ref.  -25.60 | -45.43 | -5.77 | **0.01** |  | Ref.  -3.39 | -6.38 | -0.39 | **0.02** |  |
| Cutpoint  All  Pate  Sirard  Pfeiffer |  | 9  3  6 | 21  7  14 | Ref.  19.61  -35.85  37.37 | -4.88  -55.07  -2.86 | 44.11  -6.63  67.61 | 0.11  **0.02**  0.06 |  | Ref.  3.39  -5.08  5.30 | -0.28  -9.53  -0.80 | 7.06  -0.63  11.41 | 0.06  **0.02**  0.08 |  |
| Weartime |  | 26 | 59 | 0.16 | 0.02 | 0.29 | **0.02** |  | -0.006 | -0.02 | 0.01 | 0.50 |  |
| Risk of bias |  | 26 | 59 | -0.91 | -6.99 | 5.15 | 0.76 |  | -0.18 | -1.07 | 0.71 | 0.68 |  |

Electronic supplementary material table 4s. Summary of School setting results of meta-regression multivariate models by outcomes.

|  | | | | | Mean estimate | | | | | Min/hour estimate | | | | |
| --- | --- | --- | --- | --- | --- | --- | --- | --- | --- | --- | --- | --- | --- | --- |
|  |  |  |  |  | β | 95% CI | | p | Adjusted  R-squared (%) | β | 95% CI | | p | Adjusted R-squared (%) |
|  |  |  |  |  |  | Lower | Upper |  |  |  | Lower | Upper |  |  |
|  | | Mean (range) | k | e |  |  |  |  |  |  |  |  |  |  |
| **Sedentary time** |  | |  |  |  |  |  | **<0.01** | **43.8** |  |  |  | **<0.01** | **16.7** |
| Sex  All (combined estimates)  Boys  Girls |  | | 20  19 | 37  36 | Ref.  -17.17  2.29 | -31.76  -12.35 | -2.59  17.35 | **0.02**  0.76 |  | Ref.  -2.82  0.30 | -5.21  -2.77 | -0.43  2.15 | **0.02**  0.80 |  |
| Age | 10.6 (6.0 – 15.5) | | 44 | 130 | 3.67 | 0.72 | 6.61 | **0.01** |  | 0.65 | 0.17 | 1.14 | **<0.01** |  |
| Sample size | 233.6 (19 – 1,908) | | 44 | 130 | 0.04 | 0.02 | 0.07 | **<0.01** |  | 0.008 | 0.004 | 0.01 | **<0.01** |  |
| Global Region  All  Europe  North America |  | | 22  13 | 79  31 | Ref.  -11.06  26.67 | -26.15  8.16 | 4.03  35.25 | 0.14  **<0.01** |  | Ref.  -2.05  4.87 | -4.51  1.95 | 0.41  7.78 | 0.10  **<0.01** |  |
| Study design  All  Cross-sectional |  | | 28 | 87 | Ref.  -5.65 | -20.09 | 8.77 | 0.43 |  | Ref.  -1.81 | -4.16 | 0.53 | 0.12 |  |
| Weartime | 364.1 (252.0 – 538.0) | | 44 | 130 | 0.68 | 0.53 | 0.83 | **<0.01** |  | 0.01 | -0.009 | 0.03 | 0.23 |  |
| Risk of bias | 19.2 (15 – 22) | | 44 | 130 | 1.46 | -2.98 | 5.92 | 0.51 |  | 0.31 | -0.41 | 1.04 | 0.39 |  |
| **Light Physical Activity** |  | |  |  |  |  |  | 0.11 | 6.0 |  |  |  | 0.43 | - 0.2 |
| Sex  All (combined estimates)  Boys  Girls |  | | 10  10 | 12  12 | Ref.  -18.27  -9.16 | -41.60  -33.22 | 5.05  14.73 | 0.12  0.44 |  | Ref.  -2.09  - 0.82 | -5.78  -4.57 | 1.59  2.93 | 0.26  0.66 |  |
| Age |  | | 29 | 59 | -2.57 | -7.29 | 2.14 | 0.27 |  | -0.35 | -1.10 | 0.39 | **0.34** |  |
| Sample size |  | | 29 | 59 | 0.001 | -0.04 | 0.04 | 0.97 |  | -0.0002 | -0.007 | 0.007 | **0.94** |  |
| Global Region  All  Europe  North America |  | | 15  9 | 29  18 | Ref.  -2.82  4.10 | -23.90  -22.03 | 18.25  30.24 | 0.78  0.75 |  | Ref.  -0.34  0.62 | -3.64  -3.46 | 2.95  4.72 | 0.83  0.59 |  |
| Study design  Other design  Cross-sectional |  | | 21 | 42 | Ref.  -14.71 | -36.32 | 6.86 | 0.17 |  | Ref.  -2.07 | -5.47 | 1.31 | 0.22 |  |
| Weartime | 366.8 (259.9 – 479.9) | | 29 | 59 | 0.20 | 0.02 | 0.39 | **0.02** |  | -0.02 | -0.05 | 0.007 | 0.13 |  |
| Risk of bias | 19.1 (12 – 22) | | 29 | 59 | 2.09 | -3.80 | 7.99 | 0.47 |  | 0.25 | -0.67 | 1.19 | 0.58 |  |
| **Moderate Physical Activity** |  | |  |  |  |  |  | 0.67 | -4.3 |  |  |  | 0.60 | -3.2 |
| Age | 10.1 (7.1 – 12.4) | | 16 | 40 | -1.58 | -5.47 | 2.29 | 0.41 |  | -0.21 | -0.96 | 0.41 | 0.42 |  |
| Sample size | 223.1 (15 – 1.908) | | 16 | 40 | 0.001 | -0.01 | 0.01 | 0.90 |  | 0.0006 | -0.002 | 0.002 | 0.95 |  |
| Weartime | 356.4 (259 – 473.6) | | 16 | 40 | 0.03 | -0.08 | 0.14 | 0.58 |  | -0.079 | -0.02 | 0.01 | 0.43 |  |
| Risk of bias | 18.2 (12 – 20) | | 16 | 40 | -2.92 | -7.33 | 1.49 | 0.18 |  | -0.45 | -1.24 | 0.32 | 0.24 |  |
| **Vigorous Physical Activity** |  | |  |  |  |  |  | 0.59 | -2.5 |  |  |  | 0.79 | -5.1 |
| Sex  All (combined estimates)  Boys  Girls |  | | 10  9 | 12  11 | Ref.  2.58  -2.50 | -0.38  -5.56 | 5.55  0.56 | 0.08  0.10 |  | Ref.  0.41  -0.40 | -0.08  -0.91 | 0.90  0.10 | 0.09  0.12 |  |
| Age | 10.1 (7.1 – 12.4) | | 19 | 48 | -0.06 | -1.01 | 0.88 | 0.89 |  | -0.007 | -0.15 | 0.15 | 0.98 |  |
| Sample size | 347.7 (19 – 2,119) | | 19 | 48 | 0.0003 | -0.002 | 0.003 | 0.77 |  | -0.0008 | -0.0003 | 0.0005 | 0.78 |  |
| Weartime | 356.4 (259 – 473.6) | | 19 | 48 | 0.01 | -0.01 | 0.04 | 0.32 |  | -0.001 | -0.006 | 0.003 | 0.55 |  |
| Risk of bias | 18.7 (12 – 21) | | 19 | 48 | -0.64 | -1.58 | 0.29 | 0.17 |  | -0.008 | -0.24 | 0.06 | 0.26 |  |
| **Moderate to Vigorous Physical Activity** | | |  |  |  |  |  | **<0.01** | **7.3** |  |  |  | **<0.01** | **6.0** |
| Sex  All (combined estimates)  Boys  Girls |  | | 29  28 | 50  48 | Ref.  9.90  -1.93 | 5.65  -6.38 | 14.16  2.52 | **<0.01**  0.39 |  | Ref.  1.58  -0.30 | 0.87  -1.05 | 2.29  0.43 | **<0.01**  0.41 |  |
| Age | 10.2 (5 – 15.5) | | 75 | 198 | -0.43 | -1.25 | 0.39 | 0.30 |  | -0.07 | -0.23 | 0.03 | 0.25 |  |
| Sample size | 238.3 (18 – 2,119) | | 75 | 198 | -0.005 | -0.01 | 0.001 | 0.09 |  | -0.0009 | -0.001 | 0.0004 | 0.09 |  |
| Global Region  All  Oceania  Europe  North America |  | | 11  28  32 | 23  90  72 | Ref.  6.35  -0.64  -5.78 | 0.24  -4.83  -10.17 | 12.46  3.53  -1.40 | **0.04**  0.76  **<0.01** |  | Ref.  1.19  -0.17  -0.89 | 0.17  -0.86  -1.62 | 2.20  0.52  -0.16 | **0.02**  0.62  **0.01** |  |
| Study design  All  Cross-sectional |  | | 56 | 119 | Ref.  -1.08 | -5.06 | 2.89 | 0.59 |  | Ref.  -0.18 | -0.84 | 0.47 | 0.58 |  |
| Cutpoint  All  Evenson |  | | 39 | 89 | Ref.  -5.98 | -9.91 | -2.01 | **<0.01** |  | Ref.  -1.08 | -1.78 | -0.43 | **<0.01** |  |
| Weartime | 364.2 (252 – 480) | | 75 | 198 | 0.05 | -0.002 | 0.11 | 0.06 |  | -0.004 | -0.01 | 0.004 | 0.34 |  |
| Risk of bias | 18.7 (12 – 22) | | 75 | 198 | -1.68 | -2.63 | -0.74 | **<0.01** |  | -0.28 | -0.43 | -0.12 | **<0.01** |  |

Electronic supplementary material table 5s. Summary of Afterschool setting results of meta-regression multivariate models by outcomes.

|  | | | | Mean estimate | | | | | Min/hour estimate | | | | |
| --- | --- | --- | --- | --- | --- | --- | --- | --- | --- | --- | --- | --- | --- |
|  |  |  |  | β | 95% CI | | p | Adjusted R-squared (%) | β | 95% CI | | p | Adjusted R-squared (%) |
|  |  |  |  |  | Lower | Upper |  |  |  | Lower | Upper |  |  |
|  | Mean (range) | k | e |  |  |  |  |  |  |  |  |  |  |
| **Sedentary time** |  |  |  |  |  |  | 0.16 | 7.6 |  |  |  | **<0.01** | **10.6** |
| Sex  Boys  Girls |  | 7  8 | 9  10 | Ref.  -2.92  2.80 | -11.15  -1.25 | 5.65  6.87 | 0.48  0.16 |  | Ref.  -1.86  1.78 | -6.01  -0.12 | 2.28  3.70 | 0.36  0.06 |  |
| Age | 8.2 (5.5 – 11.0) | 10 | 28 | -1.46 | -4.91 | 1.99 | 0.39 |  | -0.79 | -2.48 | 0.89 | 0.33 |  |
| Sample size | 317.1 (61 – 789) | 10 | 28 | 0.04 | 0.001 | 0.08 | **0.04** |  | 0.02 | -0.005 | 0.04 | 0.06 |  |
| Weartime | 126.0 (109 – 150.9) | 10 | 28 | -0.43 | -0.88 | 0.03 | 0.06 |  | -0.39 | -0.61 | -0.17 | **<0.01** |  |
| Risk of bias | 21.8 (20 – 22) | 10 | 28 | 2.64 | -3.30 | 8.58 | 0.36 |  | 1.13 | -1.70 | 3.96 | 0.42 |  |
| **Light Physical Activity** |  |  |  |  |  |  | **<0.01** | **52.7** |  |  |  | 0.21 | 11.0 |
| Sex (combined estimates)  Boys  Girls |  | 8  8 | 9  10 | Ref.  0.26  1.24 | -9.74  -8.92 | 10.28  11.40 | 0.95  0.79 |  | Ref.  -0.64  0.65 | -5.43  -4.22 | 4.14  5.52 | 0.77  0.78 |  |
| Age | 8.3 (5.5 – 11.0) | 9 | 21 | 0.55 | -3.08 | 4.19 | 0.75 |  | 0.15 | -1.59 | 1.89 | 0.85 |  |
| Sample size | 343.4 (61 – 1,078) | 9 | 21 | -0.002 | -0.03 | 0.02 | 0.98 |  | -0.008 | -0.01 | 0.01 | 0.90 |  |
| Weartime | 123.1 (103 – 150.9) | 9 | 21 | 0.78 | 0.33 | 1.23 | **<0.01** |  | 0.18 | -0.03 | 0.39 | 0.09 |  |
| Risk of bias | 21.3 (20 -22) | 9 | 21 | -2.51 | -9.83 | 4.80 | 0.47 |  | -1.11 | -4.61 | 2.39 | 0.51 |  |
| **Moderate Physical Activity** |  |  |  |  |  |  | 0.63 | -1.2 |  |  |  | 0.33 | 10.1 |
| Age | 8.4 (7.4 – 10.1) | 6 | 13 | 1.17 | -1.45 | 3.80 | 0.33 |  | 0.74 | -1.02 | 1.82 | 0.26 |  |
| Sample size | 307.8 (61 – 1,078) | 6 | 13 | -0.003 | -0.01 | 0.006 | 0.46 |  | -0.002 | -0.01 | 0.002 | 0.43 |  |
| Weartime | 118.5 (103.7 – 139.1) | 6 | 13 | 0.05 | -0.16 | 0.26 | 0.61 |  | -0.01 | -0.12 | 0.09 | 0.82 |  |
| Risk of bias | 21.3 (20 – 22) | 6 | 13 | 1.54 | -1.02 | 4.11 | 0.20 |  | 0.87 | -0.17 | 2.15 | 0.19 |  |
| **Vigorous Physical Activity** |  |  |  |  |  |  | 0.27 | 6.9 |  |  |  | 0.60 | -6.2 |
| Age | 8.4 (5.5 – 10.1) | 8 | 22 | -1.07 | -2.41 | 0.26 | 0.10 |  | -0.45 | -1.08 | 0.18 | 0.15 |  |
| Sample size | 297.1 (39 – 1,078) | 8 | 22 | 0.0007 | -0.01 | 0.01 | 0.88 |  | -0.0005 | -0.004 | 0.005 | 0.83 |  |
| Weartime | 128.8 (103.7 – 150.9) | 8 | 22 | 0.04 | -0.10 | 0.19 | 0.53 |  | -0.01 | -0.08 | 0.06 | 0.74 |  |
| Risk of bias | 20.5 (17 – 22) | 8 | 22 | 0.0007 | -1.33 | 1.34 | 0.99 |  | -0.007 | -0.64 | 0.62 | 0.98 |  |
| **Moderate to vigorous Physical Activity** | |  |  |  |  |  | 0.20 | 7.3 |  |  |  | **0.11** | **12.1** |
| Sex (combined estimates)  Boys  Girls |  | 7  8 | 9  10 | Ref.  2.50  -5.92 | -1.89  -9.54 | 6.29  -2.30 | 0.25  **<0.01** |  | Ref.  1.25  -3.01 | -0.99  -4.86 | 3.50  -1.16 | 0.26  **<0.01** |  |
| Age | 8.5 (5.5 – 11.0) | 14 | 31 | -1.51 | -3.04 | 0.02 | 0.06 |  | -0.53 | -1.31 | 0.25 | 0.17 |  |
| Sample size | 264.4 (39 – 1,078) | 14 | 31 | -0.005 | -0.01 | 0.005 | 0.33 |  | -0.002 | -0.008 | 0.002 | 0.30 |  |
| Weartime | 128.7 (103 – 180) | 14 | 31 | 0.05 | -0.03 | 0.14 | 0.24 |  | -0.03 | -0.08 | 0.01 | 0.11 |  |
| Risk of bias | 20.4 (17 – 22) | 14 | 31 | 0.83 | -1.51 | 1.34 | 0.90 |  | 0.01 | -0.71 | 0.75 | 0.95 |  |

Electronic supplementary material table 6s. Summary of Sport program /Physical activity results of meta-regression multivariate models by outcomes.

|  |  | | | Mean estimate | | | | | Min/hour estimate | | | | |
| --- | --- | --- | --- | --- | --- | --- | --- | --- | --- | --- | --- | --- | --- |
|  |  |  |  | β | 95% CI | | p | Adjusted R-squared (%) | β | 95% CI | | p | Adjusted R-squared (%) |
|  | Mean (range) | k | e |  | Lower | Upper |  |  |  | Lower | Upper |  |  |
| **Sedentary Time** |  |  |  |  |  |  | **<0.01** | **54.7** |  |  |  | **<0.01** | **26.4** |
| Sex (combined estimates)  Boys  Girls |  | 4  8 | 5  16 | Ref.  -1.46  6.09 | -8.81  1.26 | 5.90  10.92 | 0.69  **0.01** |  | Ref.  0.64  6.07 | -6.64  1.30 | 7.94  10.84 | 0.85  **0.01** |  |
| Age | 11.3 (7.5 – 15.6) | 16 | 38 | -0.42 | -0.89 | 1.74 | 0.51 |  | -0.41 | -1.64 | 0.81 | 0.49 |  |
| Sample size | 94.3 (19 – 555) | 16 | 38 | -0.01 | -0.03 | 0.005 | 0.12 |  | -0.002 | -0.02 | 0.01 | 0.79 |  |
| Weartime | 68.2 (27.5 – 111) | 16 | 38 | 0.28 | 0.16 | 0.39 | **<0.01** |  | -0.01 | -0.11 | 0.08 | 0.74 |  |
| Risk of bias | 18.7 (16 – 22) | 16 | 38 | -1.85 | -3.27 | -0.43 | **0.01** |  | -2.47 | -3.73 | -1.21 | **<0.01** |  |
| **Light Physical Activity** |  |  |  |  |  |  | **<0.01** | **68.1** |  |  |  | **<0.01** | **45.7** |
| Age | 11.7 (7.8 – 15.6) | 13 | 34 | 2.86 | 1.48 | 4.24 | **<0.01** |  | 2.59 | 1.38 | 3.80 | **<0.01** |  |
| Sample size | 90.9 (19 – 555) | 13 | 34 | 0.007 | -0.01 | 0.02 | 0.48 |  | 0.002 | -0.02 | 0.02 | 0.79 |  |
| Weartime | 70.2 (35 – 111) | 13 | 34 | 0.12 | 0.01 | 0.23 | **0.03** |  | -0.20 | -0.30 | -0.10 | **<0.01** |  |
| Risk of bias | 18.8 (16 – 21) | 13 | 34 | 1.77 | 0.26 | 3.28 | **0.02** |  | 1.48 | 0.16 | 2.81 | **0.01** |  |
| **Moderate Physical Activity** |  |  |  |  |  |  | **<0.01** | **67.1** |  |  |  | **<0.01** | **43.2** |
| Age | 11.7 (7.8 – 15.6) | 13 | 33 | -2.00 | -2.77 | -1.24 | **<0.01** |  | -1.73 | -2.49 | -0.98 | **<0.01** |  |
| Sample size | 91.4 (19 – 555) | 12 | 33 | 0.001 | -0.01 | 0.01 | 0.80 |  | 0.003 | -0.01 | 0.01 | 0.59 |  |
| Weartime | 71.2 (35 – 111) | 12 | 33 | 0.24 | 0.18 | 0.31 | **<0.01** |  | 0.03 | -0.03 | 0.09 | 0.33 |  |
| Risk of bias | 18.7 (16 – 21) | 12 | 33 | 0.43 | -0.37 | 1.24 | 0.28 |  | 0.28 | -0.50 | 1.07 | 0.47 |  |
| **Vigorous Physical Activity** |  |  |  |  |  |  | **<0.01** | **46.2** |  |  |  | 0.35 | 2.4 |
| Age | 11.7 (7.8 – 15.6) | 13 | 33 | -0.38 | -1.66 | 0.88 | 0.54 |  | -0.61 | -1.81 | 0.58 | 0.30 |  |
| Sample size | 92.0 (19 – 555) | 13 | 33 | -0.01 | -0.03 | 0.008 | 0.25 |  | -0.003 | -0.02 | 0.01 | 0.72 |  |
| Weartime | 68.8 (35 – 111) | 13 | 33 | 0.26 | 0.14 | 0.38 | **<0.01** |  | 0.10 | -0.001 | 0.21 | 0.06 |  |
| Risk of bias | 18.7 (16 – 21) | 13 | 33 | 0.15 | -1.14 | 1.46 | 0.80 |  | 0.28 | -0.94 | 1.51 | 0.63 |  |
| **Moderate to vigorous Physical Activity** | |  |  |  |  |  | **<0.01** | **44.1** |  |  |  | 0.26 | 3.7 |
| Sex (combined estimates)  Boys  Girls |  | 5  8 | 6  16 | Ref.  9.49  0.04 | 2.16  -5.99 | 16.51  6.08 | **<0.01**  0.98 |  | Ref.  7.50  -0.85 | 1.63  -5.84 | 13.37  4.13 | **0.01**  0.72 |  |
| Age | 11.3 (7.6 – 15.6) | 18 | 40 | -0.86 | -2.38 | 0.64 | 0.25 |  | -1.13 | -2.38 | 0.12 | 0.07 |  |
| Sample size | 99.6 (19 – 555) | 18 | 40 | -0.01 | -0.03 | 0.01 | 0.29 |  | -0.005 | -0.02 | 0.01 | 0.56 |  |
| Weartime | 67.8 (27.5 – 111) | 18 | 40 | 0.34 | 0.20 | 0.47 | <0.01 |  | 0.02 | -0.08 | 0.14 | 0.58 |  |
| Cutpoint  All  Evenson  Sirard |  | 9  7 | 16  18 | Ref.  5.02  -0.98 | -0.63  -6.91 | 10.67  4.94 | 0.07  0.73 |  | Ref.  3.19  0.45 | -1.57  -4.45 | 7.96  5.37 | 0.18  0.85 |  |
| Risk of bias | 18.7 (16 – 22) | 18 | 40 | 0.74 | -0.90 | 2.39 | 0.36 |  | 0.77 | -0.58 | 2.14 | 0.25 |  |
